# *t*-BuOK mediated oxidative coupling amination of 1,4-naphthoquinone and related 3-indolylnaphthoquinones with amines†

**DOI:** 10.1039/d1ra00193k

**Published:** 2021-02-08

**Authors:** Yu Dong, Ting Mei, Qi-Qi Luo, Qiang Feng, Bo Chang, Fan Yang, Hong-wei Zhou, Zhi-Chuan Shi, Ji-Yu Wang, Bing He

**Affiliations:** College of Chemistry and Life Science, Institute of Functional Molecules, Chengdu Normal University Chengdu 611130 P. R. China; Southwest Minzu University Chengdu 610041 P. R. China; Chengdu Institute of Organic Chemistry, Chinese Academy of Sciences Chengdu 610041 P. R. China

## Abstract

The transition-metal free amination of 1,4-naphthoquinone and related 3-indolylnaphthoquinones with amines, such as various (hetero)aromatic amine and aliphatic amine *via t*-BuOK-mediated oxidative coupling at room temperature has been developed. This reaction provides efficient access to the biologically important and synthetically useful 2-amino-1,4-naphthoquinones and 2-amino-3-indolylnaphthoquinones with good yields under mild conditions. The present protocol is simple, practical and shows good functional group tolerance. In addition, the obtained 2-amino-3-indolylnaphthoquinones were further transformed to synthesize polycyclic N-heterocycles.

## Abstract 2

The 2-amino-1,4-naphthoquinones and 2-amino-3-indolylnaphthoquinones by the *t*-BuOK mediated oxidative coupling amination has been developed. The 2-amino-3-indolylnaphthoquinones were conducted further transformation to polycyclic N-heterocycles.

## Graphical abstract

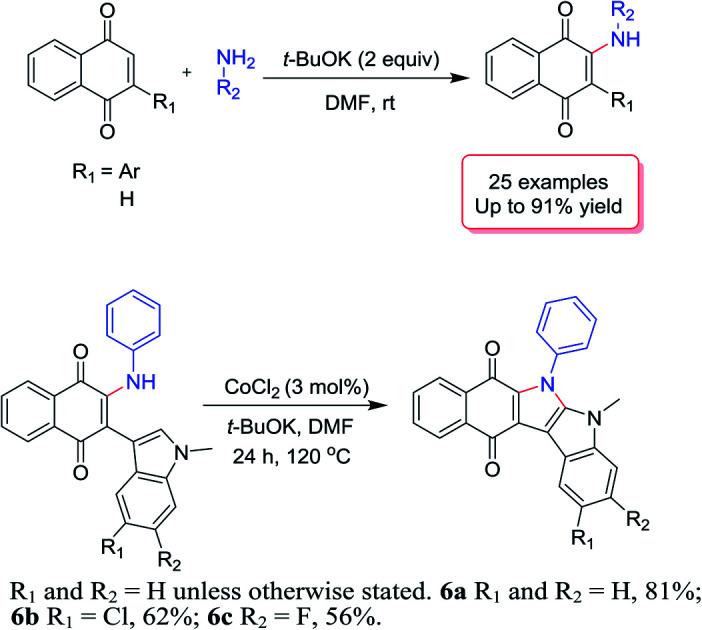

## Introduction

**Fig. 1:**
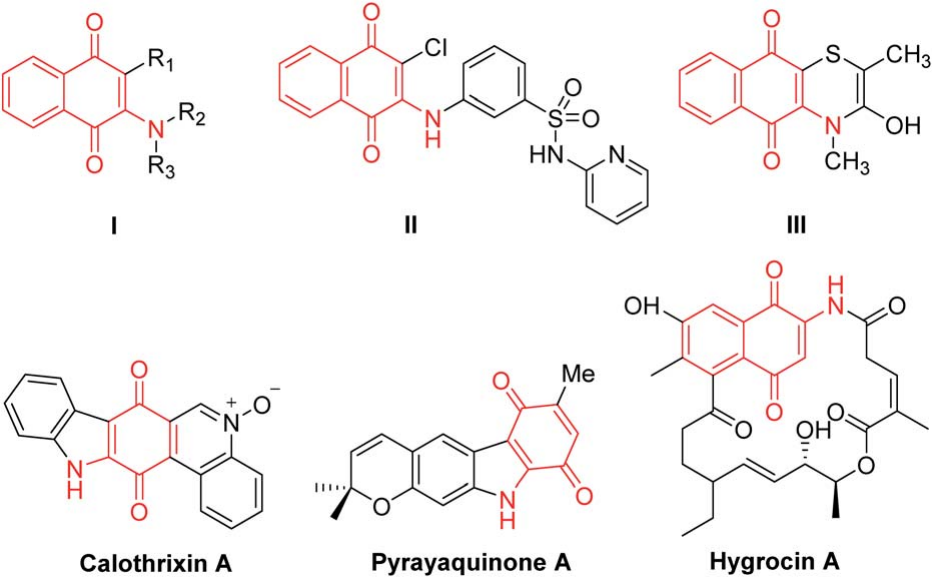
Representative biological compounds containing 2-amino-quinones skeleton.

The quinone scaffold can be found not only in various natural products and pharmaceutical compounds^1^ but it is also well-known as a versatile building block extensively applied in organic synthesis and functional materials.^2^ Among the derivatives of quinone, the 2-amino-1,4-napthoquinone (I) moiety is found in a considerable number of natural product antibiotics (Fig. 1).^3^ The representative compounds include the quinone-fused polycyclic N-heterocycles calothrixin A (antiproliferative and potent antimalarial)^4^ and hygrocins A, isolated during the purification of the immunosuppressive agent rapamycin from *Streptomyces hygroscopicus* ATC25293.^5^ Additionally, 2-amino-1,4-napthoquinone is also important as an intermediate for the synthesis of biologically active compounds.^6^

2-Amino-1,4-napthoquinone has demonstrated that the amino group in the naphthoquinone structure can change the electron-accepting capacity and therefore result in increased biological activities.^7^ What's more, this type of compound possesses several interesting biological properties such as antibacterial,^8^ antifungal,^9^ and anticancer activities.^10^ Therefore, much effort has been devoted to developing synthetic methods for the construction of 2-amino-1,4-napthoquinone derivatives. The reaction of amines with 1,4-naphthoquinone derivatives to give 2-amino-1,4-naphthoquinone have been developed with two general methods. On the one hand, 2-amino-1,4-naphthoquinones are prepared by oxidative addition coupling of amines to naphthoquinones in the presence of catalysts such as CeCl_3_·7H_2_O,^11^ FeCl_3_,^12^ Cu(OAc)_2_,^13^ I_2_,^14^ Au^15^ and HClO_4_–SiO_2_.^16^ Wang reported that 2-amino-1,4-naphthoquinones were obtained by combine the nitro reduction with the 1,4-nucleophilic addition of amines to 1,4-naphthoquinones.^17^ On the other hand, nucleophilic substitution reactions of 2-halonaphthoquinones,^18^ or 2-methoxynaphthoquinone derivatives^19^ also can afford 2-amino-1,4-naphthoquinones. The studies reported that the use of water was beneficial, resulting in nucleophilic substitution and addition reactions with quinones.^20^ The use of a bentonitic clay and ultrasonic irradiation were reported to give moderate to excellent yields of 2-amino-1,4-naphthoquinones.^21^

With the objective of studying concise routes into natural products, their analogues, and polyheteroaromatic systems with the 2-amino-1,4-naphthoquinone moiety, we initially investigated a synthetic protocol. To the best of our knowledge, an efficient synthesis of 2-amino-1,4-naphthoquinones *via* a *t*-BuOK mediated direct amination has not yet been reported. As always, we have been interested in the synthesis of indolylnaphthoquinones and related derivatives. In consideration of the important pharmaceutical applications of the unique 2-amino-1,4-naphthoquinones structural motif, we report herein a simple and practical method for the synthesis of 2-amino-1,4-naphthoquinones and 2-amino-3-indolylnaphthoquinones by the *t*-BuOK mediated oxidative coupling amination of 1,4-naphthoquinone and related 3-indolylnaphthoquinones with amines.

## Results and discussion

Our investigation to explore amination began with the reaction of indolylnaphthoquinone (1a) and aniline (2a) (see Table 1, as well as Tables S1–S4 in the ESI†). The reaction of *t*-BuOK (1.5 equiv.), and DMF (2 mL), at room temperature under air atmosphere, for 6 h (Table 1, entry 1), afforded the desired product 3a in 53% isolated yield. Encouraged by this result, subsequently, variation of K_2_CO_3_ to NaHCO_3_, KOH, NaOH, CH_3_ONa, Cs_2_CO_3_, Et_3_N, or DMAP did not show any improvement (entries 2–7; see ESI†). The use of DMF as a solvent was crucial, as the reaction gave poor results in other solvents such as DMAC, HFIP, dioxane, DCE, PhCF_3_ or DMSO, (entries 8–11; see ESI†). The reaction performed under an air atmosphere in the absence of base afforded no product (entry 12), which showed that the base played a pivotal role in obtaining the desired product. Afterward, the amount of *t*-BuOK or 2a were further screened (entries 13–14; see ESI†). It was found that the yields of the product increased with the improved in the amount of *t*-BuOK or 2a. Therefore, the optimal conditions for the preparation of 2-amino-1,4-naphthoquinones and 2-amino-3-indolylnaphthoquinones were obtained: 2a (2 equiv.), *t*-BuOK (2 equiv.), in DMF (2 mL) at room temperature for 2 h (Table 1, entry 15).

**Table 1:** Optimization of the reaction conditions^a^

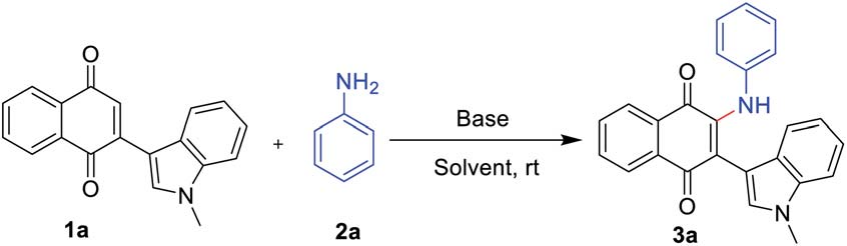				
Entry	Base (equiv.)	Solvent (2 mL)	Time (h)	Yield^b^ (%)
1	*t*-BuOK (1.5)	DMF	6	53
2	K_2_CO_3_ (1.5)	DMF	6	NR
3	NaOH (1.5)	DMF	6	33
4	CH_3_ONa (1.5)	DMF	6	NR
5	Cs_2_CO_3_ (1.5)	DMF	6	NR
6	Et_3_N (1.5)	DMF	6	NR
7	DMAP (1.5)	DMF	6	NR
8	*t*-BuOK (1.5)	DMAC	6	41
9	*t*-BuOK (1.5)	HFIP	6	NR
10	*t*-BuOK (1.5)	Dioxane	6	NR
11	*t*-BuOK (1.5)	DMSO	6	35
12	No	DMF	6	NR
13	*t*-BuOK (2)	DMF	6	71
14^c^	*t*-BuOK (2)	DMF	6	78
15^c^	*t*-BuOK (2)	DMF	2	86

a Reaction conditions: 1a (0.3 mmol), 2a (0.45 mmol, 1.5 equiv.), base (1.5–2.0 mmol), solvent (2.0 mL), 2–6 h, air, at room temperature.

b Isolated yield.

c 2a (0.6 mmol, 2 equiv.), DMF = *N*,*N*-dimethylformamide; DMAP = 4-dimethylaminopyridine; DMSO = dimethyl sulfoxide; HFIP = 1,1,1,3,3,3-hexafluoroisopropanol; NR = no reaction.

With the optimized reaction conditions in hand, subsequently, a series of substituted indolylnaphthoquinones and amines were tested for the amination (Table 2). To find the substrate scope leading to 3, a variety of substituted anilines were sequentially coupled with 3-indolylnaphthoquinone (1a) afforded the corresponding products 3 in good yields in spite of the electronic nature of aniline (3a–h, Table 2). In a 5 mmol scale reaction, 3a could be obtained in 83% yield, which indicates this transformation could be conducted in a larger scale. It is noteworthy that the valuable groups (F, Cl, Br) could be readily tolerated, which provides an opportunity for further elaboration. Even heteroanilines are well tolerated in this reaction. The use of aminopyridine provides moderate yields of the desired products (3i). The strongly coordinating groups (pyridine), which were employed as reagents for direct C–H functionalization, were fully tolerated with high chemoselectivity and regioselectivity. Next, a variety of indolylnaphthoquinones were also examined as substrates for the reaction strategy. The reaction conditions are mild and notably compatible with chloro, fluoro, and methyl on the aryl ring (3j–l, Table 2). In addition, phenylnaphthoquinone was treated with 2a under the optimized reaction conditions for the synthesis of 3m with moderate yields.

**Table 2:** Scopes with respect to 3-arylnaphthoquinones with amines^a^

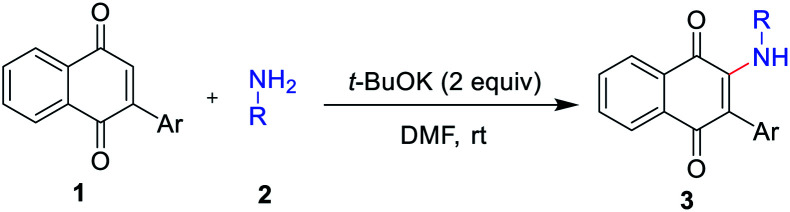
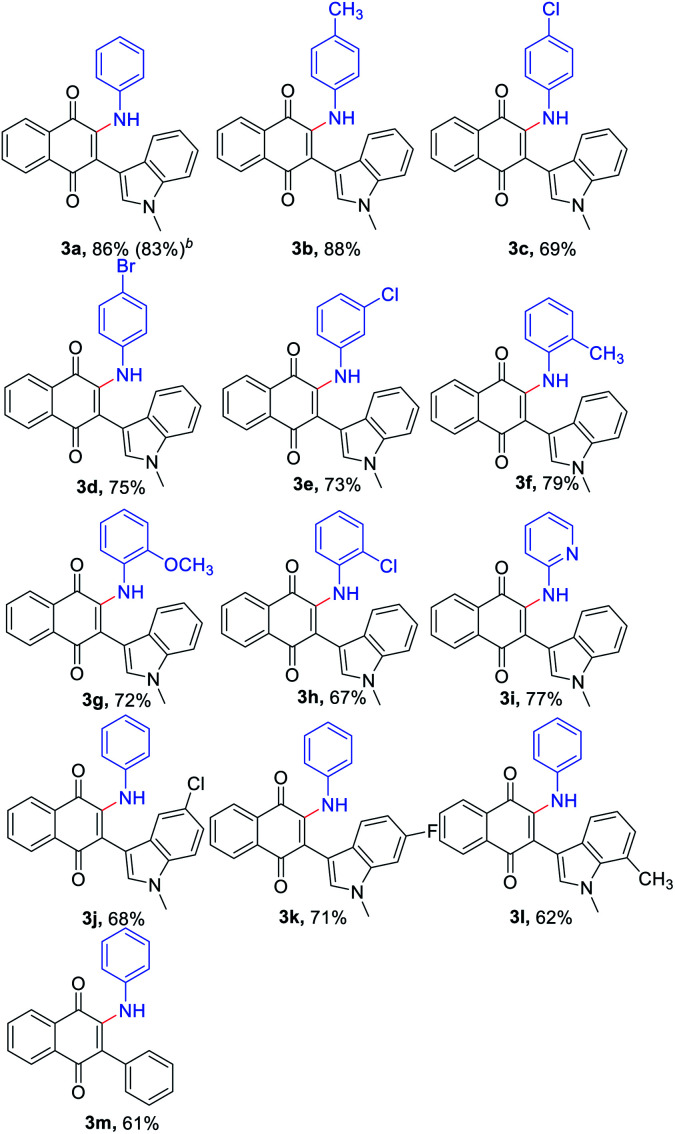

a Reaction conditions: 1 (0.3 mmol), 2 (0.6 mmol), *t*-BuOK (2.0 equiv.), DMF (2.0 mL), 2 h, air, rt. Isolated yield.

b In a 5 mmol scale.

The reaction scope was next examined by using different substituted anilines with 1,4-naphthoquinone 4a as a model substrate (Table 3). Various anilines with different electronic and steric nature were tolerated under the reaction conditions to afford 2-amino-1,4-naphthoquinones 5a–5h in moderate to high yields. In a 5 mmol scale reaction, 5a could be obtained in 89% yield, which indicates this transformation could be conducted in a larger scale. Naphthylamine and disubstituted aniline were also suitable reaction partners to give corresponding products 5i and 5j. Especially, heteroanilines and aliphatic amine were amenable under our reaction conditions and provided the expected coupling product 5k and 5l.

**Table 3:** Scopes with respect to 1,4-naphthoquinone with amines^a^

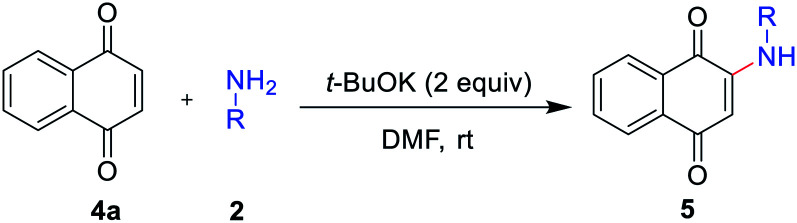
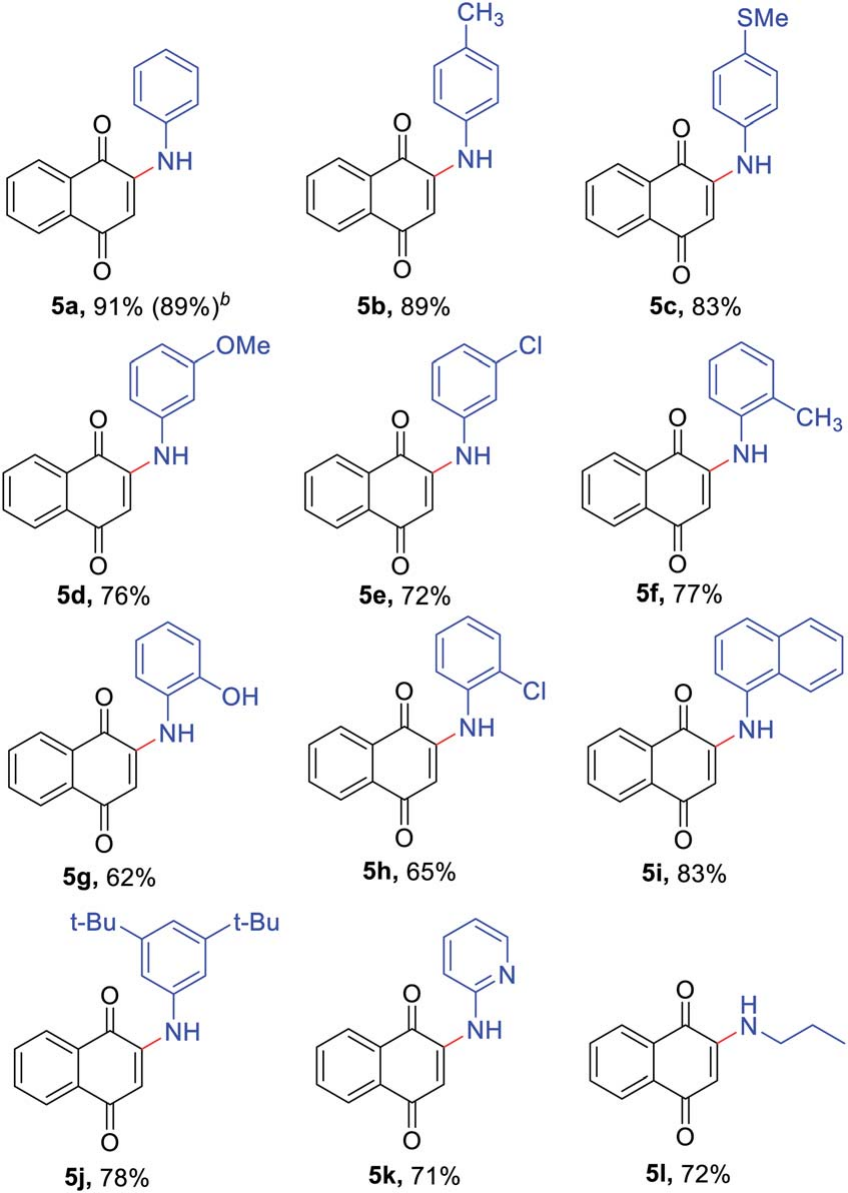

a Reaction conditions: 1,4-naphthoquinone (0.3 mmol), 2 (0.6 mmol), *t*-BuOK (2.0 equiv.), DMF (2.0 mL), 2 h, air, rt. Isolated yield.

b In a 5 mmol scale.

**Scheme 1:**
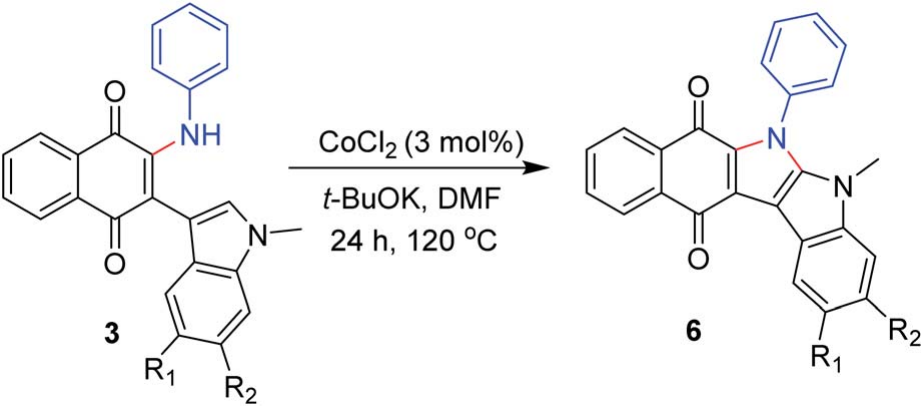
R_1_ and R_2_ = H unless otherwise stated. 6a R_1_ and R_2_ = H, 81%; 6b R_1_ = Cl, 62%; 6c R_2_ = F, 56%.

Polycyclic N-heterocycles are the key structural element of natural products, drugs and functional materials.^22^ Therefore, a Co-catalyzed intramolecular cyclization reaction of some of the 2-amino-3-indolylnaphthoquinones derivatives 3 allowed the generation of polycyclic N-heterocycles derivatives 6 in moderate yields (Scheme 1).

**Scheme 2:**
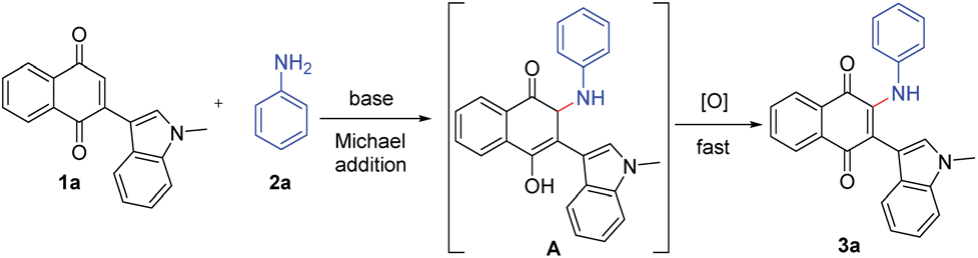
Plausible reaction mechanism.

On the basis of our and previous reports,^23^ a possible reaction mechanism was proposed (Scheme 2). Initially, the Michael addition of indolylnaphthoquinone (1a) and aniline (2a) in the presence of base gave the intermediate A, which was immediately oxidized to the product 3a by O_2_ or the oxidative naphthoquinone.

## Conclusions

In conclusion, we have developed a practical and efficient strategy for *t*-BuOK-mediated oxidative coupling amination of 1,4-naphthoquinone and related 3-indolylnaphthoquinones with amines at room temperature. A series of 2-amino-1,4-naphthoquinones and 2-amino-3-indolylnaphthoquinones were conveniently synthesized in good yields under air conditions. The reaction took place under mild conditions, displayed excellent functional group compatibility, and did not use metals. In addition, the obtained 2-amino-3-indolylnaphthoquinones derivatives were conducted further transformation to synthesize polycyclic N-heterocycles.

## Conflicts of interest

There are no conflicts to declare.

## Supplementary Material

RA-011-D1RA00193K-s001
