# Supplementary material for: t-BuOK mediated oxidative coupling amination of 1,4-naphthoquinone and related 3-indolylnaphthoquinones with amines
Source: RSC Adv. 2021 Feb 8;11(12):6776–80. doi: 10.1039/d1ra00193k (PMC8694889; doi:10.1039/d1ra00193k)
Supplement: RA-011-D1RA00193K-s001 [file RA-011-D1RA00193K-s001.pdf]

Electronic Supplementary Material (ESI) for RSC Advances.
This journal is © The Royal Society of Chemistry 2021

t-BuOK Mediated Oxidative Coupling Amination of 1,4-Naphthoquinone and Related 3-Indolylnaphthoquinones with Amines

Yu Dong,[a] Ting Mei,[a] Qi-Qi Luo,[a] Qiang Feng,[a] Bo Chang,[a] Fan Yang,[a] Hong-wei Zhou,[a] Zhi-Chuan Shi,[b] Ji-Yu Wang,*[c] and Bing He*[a]

[a] College of Chemistry and Life Science, Institute of Functional Molecules, Chengdu Normal University, Chengdu, 611130, PR China.
[b] Southwest Minzu University, Chengdu 610041, P. R. China.
[c] Chengdu Institute of Organic Chemistry, Chinese Academy of Sciences, Chengdu 610041, P. R. China.

Table of Content

1. General information S2

2. Preparation of the starting materials S2

3. General procedure for the synthesis of compounds S3

4. Optimization of reaction conditions S4

5. Procedure for gram-scale reaction S6

6. Characterization data of products S7

7. References S18

8. Copies of ^{1}H, ^{13}C and ^{19}F NMR spectra for all compounds S18

1. General information

Chemicals and analytical grade solvents were purchased from commercial suppliers and used without further purification unless otherwise stated. All reagents were weighed and handled in air at room temperature. Analytical thin–layer chromatography was performed on glass plates of Silica Gel GF–254 with detection by UV light (254 and 365 nm). Column chromatography was carried out on silica gel (200-300 mesh). ^{1}H NMR spectra were recorded at 400 MHz and ^{13}C NMR spectra were recorded at 101 MHz by using Agilent 400 MHz NMR spectrometer. Chemical shifts were calibrated using residual undeuterated solvent as an internal reference (^{1}H NMR: $CDCl_3$ 7.26 ppm, DMSO-d_6 2.50 ppm, ^{13}C NMR: $CDCl_3$ 77.16 ppm, DMSO-d_6 39.52 ppm). Data are reported as follows: chemical shift, multiplicity (s = singlet, br s = broad singlet, d = doublet, t = triplet, q = quartet, m = multiplet), Coupling constants (*J*) were reported in Hertz (Hz). HRMS were performed on a Thermo Scientific LTQ Orbitrap XL instrument. Melting points were measured with micro melting point apparatus.

2. Preparation of the starting materials

For this study, *N*-Protected indoles were prepared from substituted indoles with iodomethane.[1] Indoles-substituted 1,4-naphthoquinones (**1**) were prepared from *N*-Protected indoles with 1,4-naphthoquinones.[2]

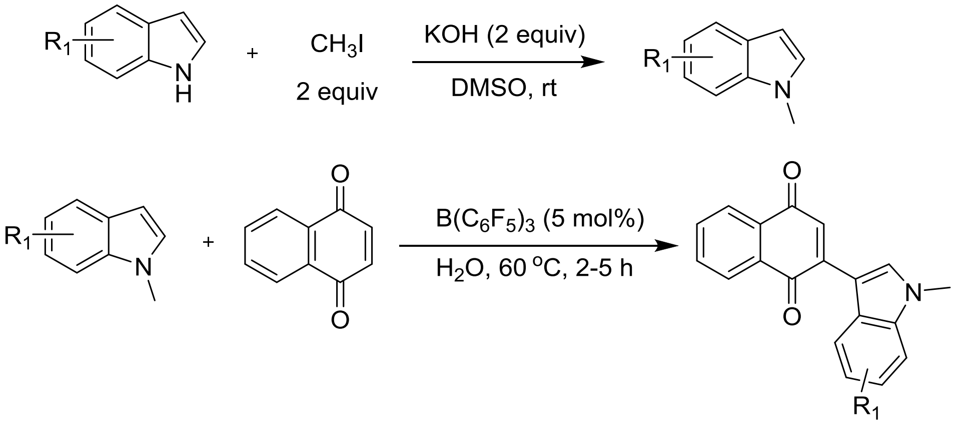

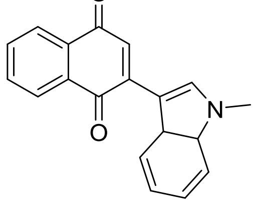

1a

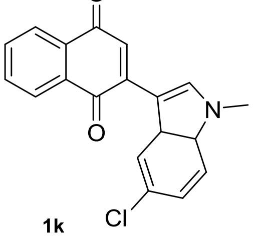

1k

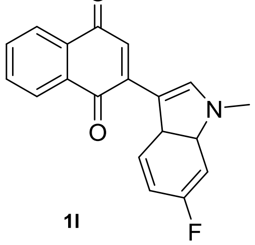

1l

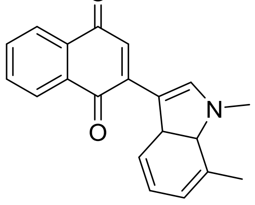

1m

3. General procedure for the synthesis of the compounds

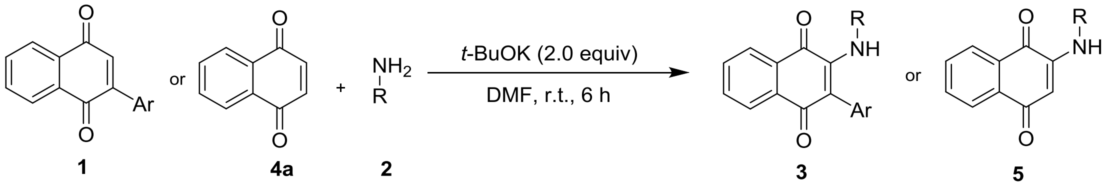

To a solution of indolylnaphthoquinon 、 phenylnaphthoquinones or 1,4-naphthoquinone (0.3 mmol), *t*-BuOK (0.6 mmol, 2 equiv) in DMF (2 mL) was added amines **2** (0.6 mmol, 2 equiv). The reaction mixture was stirred at room temperature under air atmosphere for 2 h. After the completion of the reaction (monitored by TLC). The reaction was quenched with saturated salt water (8 ml) and the mixture was extracted with EtOAc (3 × 3 mL). The organic extracts were washed with brine, dried over Na_2SO_4, filtered and the solvent was removed in vacuo. The crude product was purified by silica gel column chromatography to give **3** or **5**.

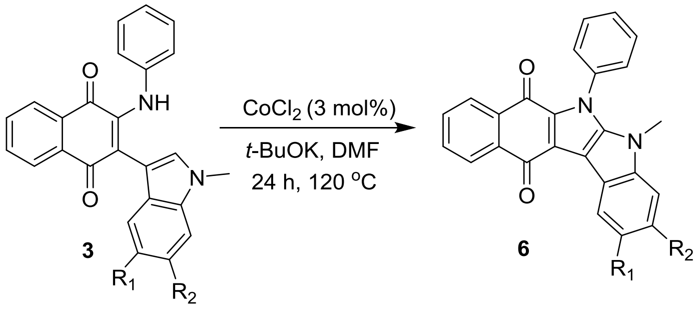

To a solution of the 2-amino-3-indolylnaphthoquinones derivatives **3** (0.3 mmol), *t*-BuOK (0.45 mmol, 1.5 equiv) in DMF (2 mL) was added $CoCl_2$ (0.009 mmol, 3 mol %), The reaction mixture was stirred at 120 °C under air atmosphere for 24 h. After the completion of the reaction (monitored by TLC), and cooled down to room temperature. The reaction was quenched with saturated salt water (8 ml) and the mixture was extracted with EtOAc (3 × 3 mL). The organic extracts were washed with brine, dried over Na_2SO_4, filtered and the solvent was removed in vacuo. The crude product was purified by silica gel column chromatography to give **6**.

4. Optimization of reaction conditions

Table S1. Optimization of base[a]

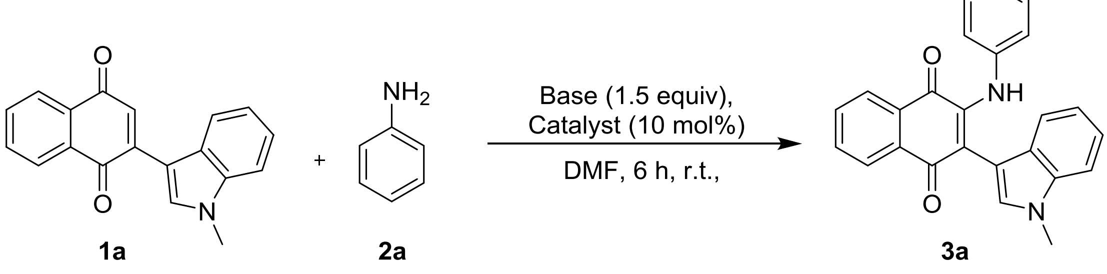

Entry	Base	Catalyst	Yield[b]
1	*t*-BuOK	No	53
2	K_2CO_3	No	NR
3	$NaHCO_3$	No	NR
4	KOH	No	31
5	NaOH	No	33
6	CH_3ONa	No	NR
7	Cs_2CO_3	No	NR
8	Et_3N	No	NR
9	DMAP	No	NR
10	No	No	NR
11	*t*-BuOK	$CoCl_2$	51
12	*t*-BuOK	$Cu(OAc)_2.H_2O$	0
13	*t*-BuOK	CuBr	0
14	*t*-BuOK	$Zn(OAc)_2$	0

[a]Reaction conditions: **1a** (0.3 mmol), **2a** (0.45 mmol), Catalyst (10 mol %), Base (1.5 equiv), DMF (2.0 mL), 6 h, at room temperature. [b]Isolated yield. N.R. = no reaction.

Table S2. Optimization of solvents[a]

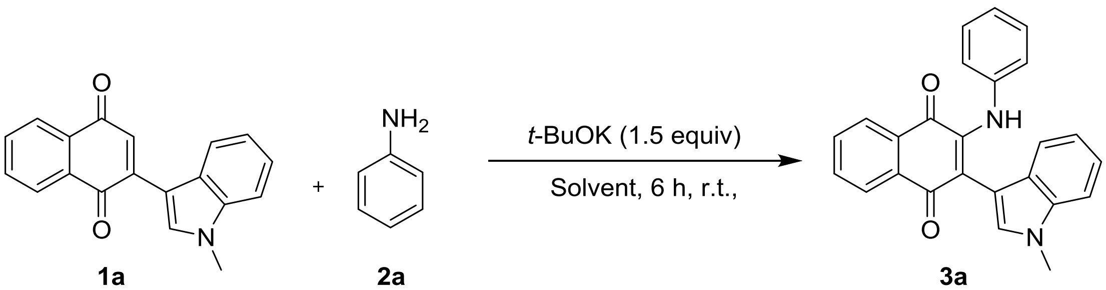

Entry	Solvent	Yield[b]
1	DMF	53
2	DMAC	41
4	EtOH	NR
5	HFIP	NR
6	Dioxane	NR
7	DCE	NR
8	CH_3CN	NR
9	Toluene	NR
10	$PhCF_3$	NR
11	DMSO	35

[a]Reaction conditions: **1a** (0.3 mmol), **2a** (0.45 mmol), *t*-BuOK (1.5 equiv), Solvent (2.0 mL), 6 h, at room temperature. [b]Isolated yield. DMF = *N*, *N*-Dimethylformamide; DMAC = *N*,*N*-Dimethylacetamide; HFIP = 1,1,1,3,3,3-Hexafluoroisopropanol; DMSO = Dimethyl sulfoxide; N.R. = no reaction.

Table S3. Optimization of dosages[a]

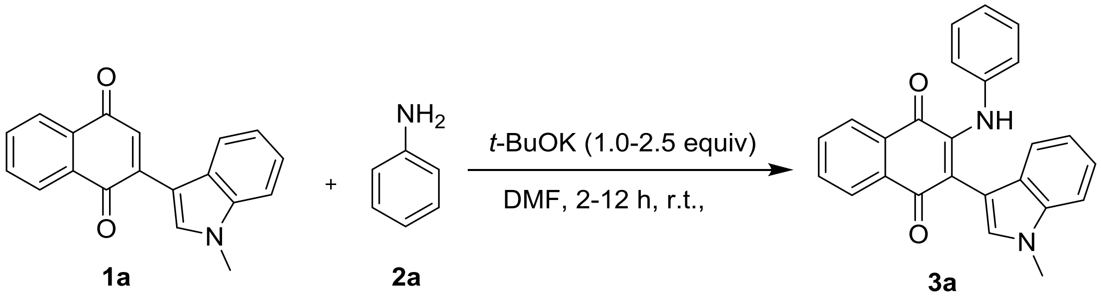

Entry	2a (equiv)	*t*-BuOK (equiv)	t (h)	Yield[b]
1	1.5	1.5	6	53
2	1.5	1.0	6	41
3	1.5	2.0	6	71
4	1.5	2.5	6	69
5	1.0	2.0	6	51
6	2.0	2.0	6	78
7	2.5	2.0	6	76
8	2.0	2.0	2	86
9	2.0	2.0	4	80
10	2.0	2.0	12	63
11[c]	2.0	2.0	2	75
12[d]	2.0	2.0	2	83

[a]Reaction conditions: **1a** (0.3 mmol), **2a** (0.3-0.75 mmol), *t*-BuOK (1.0-2.5 equiv), DMF (1.0 -3.0 ml), 2-12 h, at room temperature. [b]Isolated yield. [c]DMF (1.0 mL). [d]DMF (3.0 mL).

Table S4. Optimization of temperature[a]

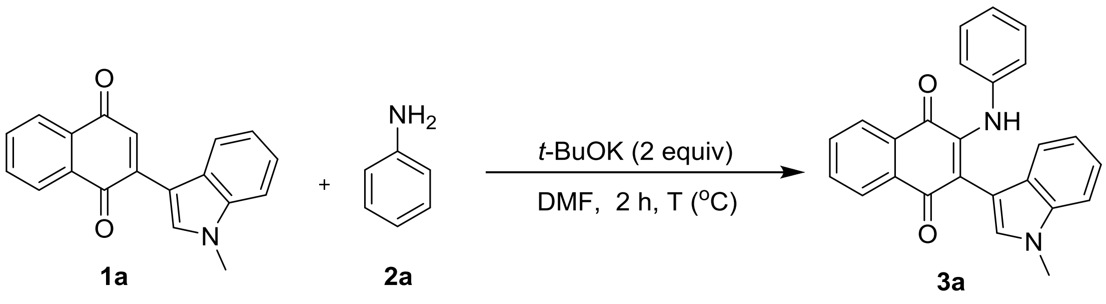

Entry	T (°C)	Yield[b]
1	25	86
2	40	79
3	60	65

[a]Reaction conditions: **1a** (0.3 mmol), **2a** (0.6 mmol), *t*-BuOK (2 equiv), DMF (2.0 mL), 2 h, 25-60 ℃. [b]Isolated yield.

5. Procedure for gram-scale reaction

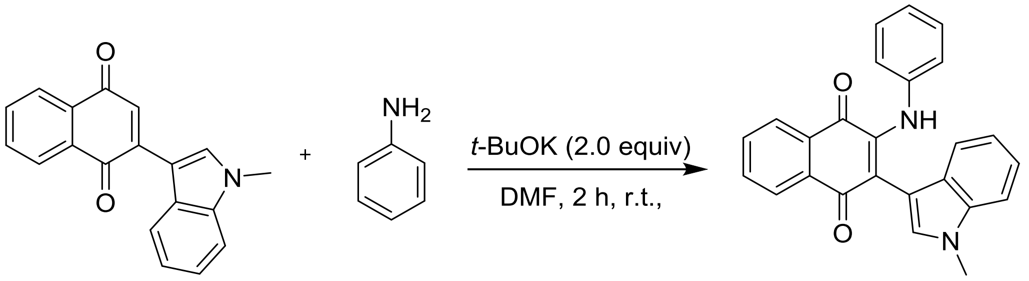

1a, 5 mmol **2a**, 10 mmol **3a**, 1.57 g, 83%

To a solution of indolylnaphthoquinone **1a** (1.43 g), *t*-BuOK (1.12 g, 2.0 equiv) in DMF (33 mL) was added aniline **2a** (0.93 g, 2.0 equiv). The reaction mixture was stirred at room temperature under air atmosphere for 2 h. After the completion of the reaction (monitored by TLC), The reaction was quenched with saturated salt water (120 ml) and the mixture was extracted with EtOAc (3 × 60 mL). The organic extracts were washed with brine, dried over Na_2SO_4, filtered and the solvent was removed in vacuo. The crude product was purified by silica gel column chromatography to give **3a** in 83% yield.

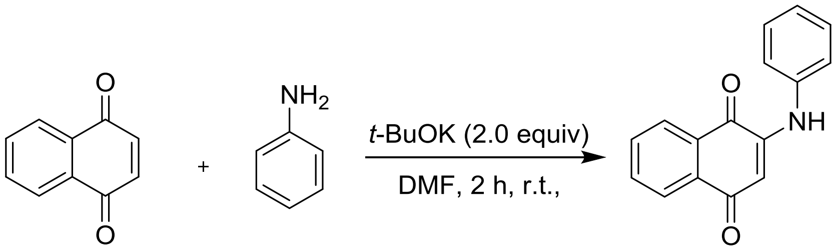

4a, 5 mmol **2a**, 10 mmol **5a**, 1.11 g, 89%

To a solution of naphthoquinone **4a** (0.79 g), *t*-BuOK (1.12 g, 2.0 equiv) in DMF (33 mL) was added aniline **2a** (0.93 g, 2.0 equiv). The reaction mixture was stirred at room temperature under air atmosphere for 2 h. After the completion of the reaction (monitored by TLC), The reaction was quenched with saturated salt water (120 ml) and the mixture was extracted with EtOAc (3 × 60 mL). The organic extracts were washed with brine, dried over Na_2SO_4, filtered and the solvent was removed in vacuo. The crude product was purified by silica gel column chromatography to give **5a** in 83% yield.

6. Characterization data of products

2-(1-methyl-1*H*-indol-3-yl)-3-(phenylamino)naphthalene-1,4-dione (3a)

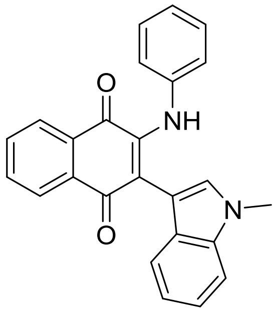

Black solid, 86% yield; mp 218-220 ℃. ^{1}H NMR (400 MHz, DMSO-d_6) δ 8.73 (s, 1H), 8.07 (dd, J = 16.0, 7.5 Hz, 2H), 7.83 (dt, J = 21.7, 7.4 Hz, 2H), 7.25 (s, 1H), 7.21 (d, J = 7.9 Hz, 1H), 7.16 (d, J = 8.1 Hz, 1H), 6.98 (t, J = 7.5 Hz, 1H), 6.89 (t, J = 7.4 Hz, 1H), 6.72 – 6.46 (m, 5H), 3.63 (s, 3H). ^{13}C NMR (101 MHz, DMSO-d_6) δ 182.81, 182.31, 141.09, 138.79, 136.43, 134.84, 133.35, 133.21, 132.44, 131.16, 129.77, 126.71, 126.54, 126.33, 126.11, 122.25, 121.48, 121.13, 120.92, 119.32, 114.79, 109.61, 107.00, 32.76. HRMS calcd. For $C_{25}H_{19}N_2O_2^+$ $(M+H)^+$ 379.1447 found: 379.1441.

2-(1-methyl-1*H*-indol-3-yl)-3-(p-tolylamino)naphthalene-1,4-dione (3b)

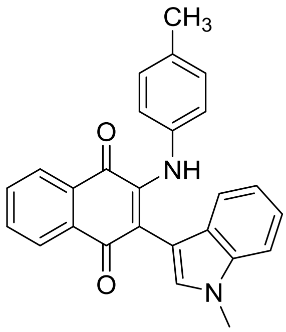

Black solid, 88% yield; mp 225-227 ℃. ^{1}H NMR (400 MHz, $CDCl_3$) δ 8.16 (ddd, J = 14.2, 7.6, 1.0 Hz, 2H), 7.73 (td, J = 7.5, 1.4 Hz, 1H), 7.69 – 7.58 (m, 2H), 7.31 (d, J = 7.9 Hz, 1H), 7.09 – 6.96 (m, 4H), 6.47 (d, J = 8.2 Hz, 2H), 6.40 (d, J = 8.3 Hz, 2H), 3.60 (s, 3H), 2.03 (s, 3H). ^{13}C NMR (101 MHz, $CDCl_3$) δ 184.36, 183.00, 140.43, 135.74, 134.95, 134.37, 132.73, 132.30, 131.22, 130.80, 127.78, 127.13, 126.64, 126.06, 121.60, 121.32, 120.89, 119.48, 113.03, 108.71, 32.62, 20.60. HRMS calcd. For $C_{26}H_{21}N_2O_2^+$ $(M+H)^+$ 393.1602 found: 393.1603.

2-((4-chlorophenyl)amino)-3-(1-methyl-1*H*-indol-3-yl)naphthalene-1,4-dione (3c)

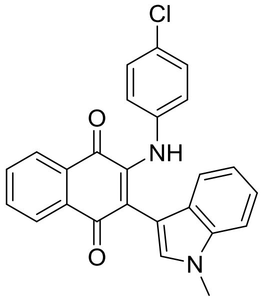

Black solid, 69% yield; mp 221-223 ℃. ^{1}H NMR (400 MHz, $CDCl_3$) δ 8.22 – 8.11 (m, 2H), 7.74 (dd, *J* = 7.5, 1.4 Hz, 1H), 7.68 (dd, *J* = 7.4, 1.3 Hz, 1H), 7.58 (s, 1H), 7.27 (d, *J* = 6.7 Hz, 2H), 7.08 (d, *J* = 3.6 Hz, 2H), 7.00 – 6.96 (m, 1H), 6.65 – 6.59 (m, 2H), 6.42 (d, *J* = 8.7 Hz, 2H), 3.68 (s, 3H). ^{13}C NMR (101 MHz, $CDCl_3$) δ 182.69, 182.38, 139.46, 139.41, 135.88, 134.46, 132.56, 131.39, 129.69, 126.74, 126.39, 126.31, 126.13, 126.10, 122.34, 122.07, 121.64, 121.61, 120.74, 119.63, 114.47, 109.05, 109.00, 106.73, 32.86. HRMS calcd. For $C_{25}H_{18}ClN_2O_2^+$ $(M+H)^+$ 413.1065 found: 413.1057.

2-((4-bromophenyl)amino)-3-(1-methyl-1*H*-indol-3-yl)naphthalene-1,4-dione (3d)

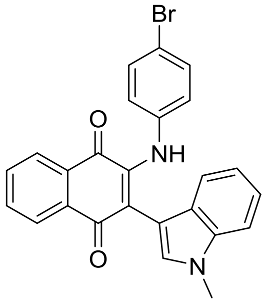

Black solid, 75% yield; mp 227-229 ℃. ^{1}H NMR (400 MHz, $CDCl_3$) δ 8.17 (t, *J* = 8.5 Hz, 2H), 7.77 – 7.67 (m, 2H), 7.57 (s, 1H), 7.25 (dd, *J* = 8.2, 3.6 Hz, 2H), 7.11 – 7.06 (m, 2H), 7.00 – 6.95 (m, 1H), 6.76 (d, *J* = 8.6 Hz, 2H), 6.36 (d, *J* = 8.7 Hz, 2H), 3.69 (s, 3H). ^{13}C NMR (101 MHz, $CDCl_3$) δ 182.69, 182.39, 139.04, 136.32, 134.53, 134.46, 132.57, 131.48, 131.39, 129.33, 129.31, 126.74, 126.14, 122.72, 122.70, 121.66, 120.73, 119.77, 116.81, 116.39, 115.60, 115.11, 109.07, 106.37, 32.87. HRMS calcd. For $C_{25}H_{18}BrN_2O_2^+$ $(M+H)^+$ 457.0558 found: 457.0552.

2-((3-chlorophenyl)amino)-3-(1-methyl-1*H*-indol-3-yl)naphthalene-1,4-dione (3e)

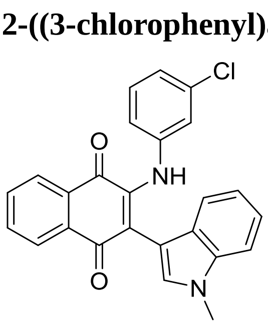

Black solid, 73% yield; mp 211-213 ℃. ^{1}H NMR (400 MHz, $CDCl_3$) δ 8.18 (dd, *J* =

10.9, 7.6 Hz, 2H), 7.77 – 7.66 (m, 3H), 7.35 (s, 1H), 7.22 (d, *J* = 7.9 Hz, 1H), 7.03 (d, *J* = 3.6 Hz, 2H), 6.96 – 6.91 (m, 1H), 6.59 – 6.48 (m, 2H), 6.45 – 6.32 (m, 2H), 3.72 (s, 3H). ^{13}C NMR (101 MHz, $CDCl_3$) δ 183.07, 182.45, 138.51, 137.94, 135.99, 134.46, 132.63, 131.26, 127.25, 126.14, 122.47, 121.56, 120.62, 120.56, 119.69, 119.40, 114.66, 114.38, 108.88, 106.90, 106.62, 32.89. HRMS calcd. For $C_{25}H_{18}ClN_2O_2^+$ $(M+H)^+$ 413.1057 found: 413.1057.

2-(1-methyl-1*H*-indol-3-yl)-3-(o-tolylamino)naphthalene-1,4-dione (3f)

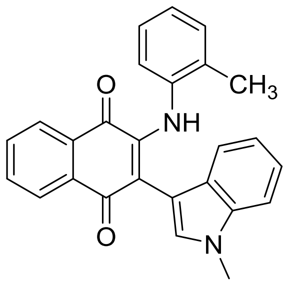

Black solid, 79% yield; mp 202-204 ℃. ^{1}H NMR (400 MHz, $CDCl_3$) δ 8.18 (ddd, *J* = 12.5, 7.7, 1.2 Hz, 2H), 7.75 (td, *J* = 7.5, 1.4 Hz, 1H), 7.68 (td, *J* = 7.5, 1.3 Hz, 1H), 7.46 (s, 1H), 7.29 (d, *J* = 7.8 Hz, 1H), 7.07 – 6.93 (m, 4H), 6.69 (d, *J* = 7.4 Hz, 1H), 6.56 (td, *J* = 7.4, 1.1 Hz, 1H), 6.44 (t, *J* = 7.1 Hz, 1H), 6.37 (d, *J* = 7.5 Hz, 1H), 3.61 (s, 3H), 2.18 (s, 3H). ^{13}C NMR (101 MHz, $CDCl_3$) δ 182.94, 140.33, 136.24, 135.77, 134.44, 133.60, 132.37, 131.01, 130.77, 129.67, 128.91, 126.92, 126.69, 126.09, 123.88, 123.56, 121.73, 121.27, 120.39, 119.22, 113.30, 109.99, 108.75, 106.62, 32.69, 18.15. HRMS calcd. For $C_{26}H_{21}N_2O_2^+$ $(M+H)^+$ 393.1604 found: 393.1603.

2-((2-methoxyphenyl)amino)-3-(1-methyl-1*H*-indol-3-yl)naphthalene-1,4-dione (3g)

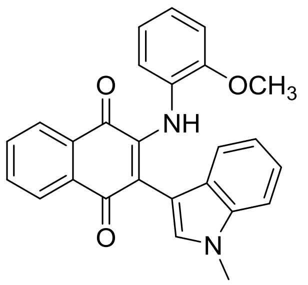

Black solid, 72% yield; mp 210-212 ℃. ^{1}H NMR (400 MHz, $CDCl_3$) δ 8.27 – 8.06 (m, 2H), 7.79 – 7.64 (m, 3H), 7.27 (dd, *J* = 17.9, 8.9 Hz, 2H), 7.02 (d, *J* = 4.1 Hz, 2H), 6.94 – 6.86 (m, 1H), 6.55 (t, *J* = 7.5 Hz, 1H), 6.32 (dd, *J* = 12.2, 8.0 Hz, 2H), 6.19 (t, *J* = 7.6 Hz, 1H), 3.69 (s, 3H), 3.66 (s, 3H). ^{13}C NMR (101 MHz, $CDCl_3$) δ 182.97, 182.84, 139.40, 136.26, 134.26, 133.69, 132.29, 131.31, 130.90, 126.83, 126.60, 126.23, 126.01, 123.43, 121.11, 120.74, 120.61, 118.99, 117.85, 113.09, 109.98, 108.75, 108.58, 106.86, 55.17, 32.81. HRMS calcd. For $C_{26}H_{21}N_2O_3^+$ $(M+H)^+$ 409.1551 found: 409.1552.

2-((2-chlorophenyl)amino)-3-(1-methyl-1*H*-indol-3-yl)naphthalene-1,4-dione (3h)

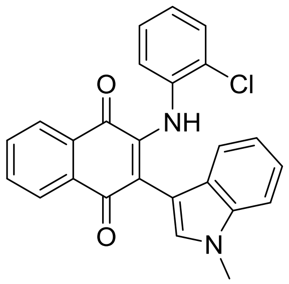

Black solid, 67% yield; mp 207-209 ℃. ^{1}H NMR (400 MHz, $CDCl_3$) δ 8.32 – 7.98 (m, 2H), 7.79 – 7.66 (m, 3H), 7.36 – 7.27 (m, 2H), 7.05 – 6.88 (m, 4H), 6.50 – 6.34 (m, 3H), 3.68 (s, 3H). ^{13}C NMR (101 MHz, $CDCl_3$) δ 183.05, 182.52, 138.50, 138.39, 136.77, 135.83, 134.39, 132.64, 131.52, 127.77, 126.72, 126.16, 124.32, 123.31, 122.55, 121.52, 121.16, 120.76, 119.78, 114.49, 108.69, 106.07, 32.89. HRMS calcd. For $C_{25}H_{18}ClN_2O_2^+$ $(M+H)^+$ 413.1059 found: 413.1057.

2-(1-methyl-1*H*-indol-3-yl)-3-(pyridin-2-ylamino)naphthalene-1,4-dione (3i)

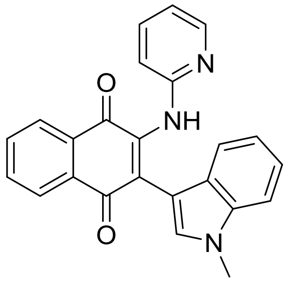

Black solid, 77% yield; mp 231-233 ℃. ^{1}H NMR (400 MHz, $CDCl_3$) δ 8.23 – 8.09 (m, 2H), 8.00 (s, 1H), 7.87 (d, *J* = 4.0 Hz, 1H), 7.75 – 7.67 (m, 2H), 7.42 (s, 1H), 7.38 (d, *J* = 7.8 Hz, 1H), 7.12 – 7.03 (m, 2H), 7.02 – 6.90 (m, 2H), 6.47 (dd, *J* = 6.8, 5.1 Hz, 1H), 6.27 (d, *J* = 8.2 Hz, 1H), 3.73 (s, 3H). ^{13}C NMR (101 MHz, $CDCl_3$) δ 183.46, 182.30, 150.88, 146.81, 138.25, 136.42, 135.20, 134.21, 133.17, 132.82, 132.01, 130.95, 126.71, 126.18, 125.77, 121.70, 121.21, 120.03, 117.15, 113.40, 108.98, 106.98, 33.00. HRMS calcd. For $C_{24}H_{18}N_3O_2^+$ $(M+H)^+$ 380.1394 found: 380.1399.

2-(5-chloro-1-methyl-1*H*-indol-3-yl)-3-(phenylamino)naphthalene-1,4-dione (3j)

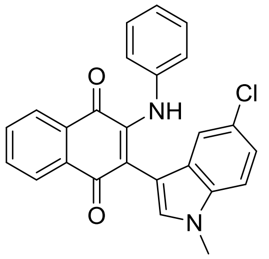

Black solid, 68% yield; mp 225-227 ℃. ^{1}H NMR (400 MHz, $CDCl_3$) δ 8.24 (t, *J* = 7.6 Hz, 2H), 7.83 – 7.72 (m, 3H), 7.34 (dd, *J* = 14.5, 0.9 Hz, 1H), 7.20 (s, 1H), 7.04 (dd, *J* = 8.6, 1.3 Hz, 1H), 6.97 (d, *J* = 8.6 Hz, 1H), 6.76 (t, *J* = 7.6 Hz, 2H), 6.68 (d, *J* = 7.1 Hz, 1H), 6.60 (d, *J* = 7.8 Hz, 2H), 3.66 (s, 3H). ^{13}C NMR (101 MHz, $CDCl_3$) δ 182.81, 182.77, 139.87, 137.23, 134.66, 134.53, 133.43, 132.53, 132.50, 130.63, 127.64, 126.71, 126.58, 126.19, 125.29, 123.24, 121.54, 121.51, 121.49, 120.47, 112.69,

109.84, 106.69, 32.94. HRMS calcd. For $C_{25}H_{18}ClN_2O_2^+$ $(M+H)^+$ 413.1056 found: 413.1057.

2-(6-fluoro-1-methyl-1*H*-indol-3-yl)-3-(phenylamino)naphthalene-1,4-dione (3k)

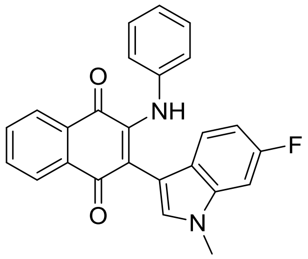

Black solid, 71% yield; mp 229-231 ℃. ^{1}H NMR (400 MHz, $CDCl_3$) δ 8.29 – 8.06 (m, 2H), 7.76 (td, *J* = 7.5, 1.3 Hz, 1H), 7.71 – 7.64 (m, 2H), 7.26 – 7.22 (m, 1H), 7.05 (s, 1H), 6.78 – 6.62 (m, 5H), 6.51 (d, *J* = 7.7 Hz, 2H), 3.55 (s, 3H). ^{13}C NMR (101 MHz, $CDCl_3$) δ 182.90, 182.84, 160.89, 158.41, 140.08, 137.26, 136.28, 136.21, 134.52, 133.48, 132.49, 131.61, 130.64, 126.72, 126.67, 126.16, 123.24, 121.74, 121.64, 121.49, 108.25, 108.01, 95.38, 95.11, 32.80. HRMS calcd. For $C_{25}H_{18}FN_2O_2^+$ $(M+H)^+$ 397.1359 found: 397.1352.

2-(1,7-dimethyl-1*H*-indol-3-yl)-3-(phenylamino)naphthalene-1,4-dione (3l)

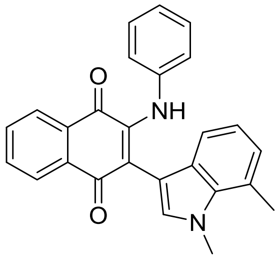

Black solid, 62% yield; mp 223-225 ℃. ^{1}H NMR (400 MHz, $CDCl_3$) δ 8.17 (dd, *J* = 12.4, 7.6 Hz, 2H), 7.78 – 7.71 (m, 1H), 7.70 – 7.60 (m, 2H), 7.10 (d, *J* = 7.9 Hz, 1H), 6.96 (s, 1H), 6.83 (t, *J* = 7.5 Hz, 1H), 6.68 (dt, *J* = 22.4, 7.2 Hz, 4H), 6.51 (d, *J* = 7.5 Hz, 2H), 3.87 (s, 3H), 2.58 (s, 3H). ^{13}C NMR (101 MHz, $CDCl_3$) δ 182.94, 182.86, 137.36, 135.01, 135.00, 134.36, 133.55, 132.77, 132.37, 130.81, 127.55, 126.67, 126.50, 126.07, 124.08, 122.90, 121.52, 120.65, 120.50, 119.76, 119.24, 118.95, 118.89, 109.98, 36.79, 19.56. HRMS calcd. For $C_{26}H_{21}N_2O_2^+$ $(M+H)^+$ 393.1609 found: 393.1603.

2-phenyl-3-(phenylamino)naphthalene-1,4-dione (3m)

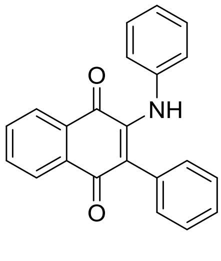

Black solid, 61% yield; mp 161-163 ℃. ^{1}H NMR (400 MHz, $CDCl_3$) δ 8.08 (dd, *J* = 11.2, 7.7 Hz, 2H), 7.68 (t, *J* = 7.4 Hz, 2H), 7.60 (d, *J* = 7.5 Hz, 1H), 7.16 (s, 1H), 6.91 (dd, *J* = 9.2, 4.1 Hz, 4H), 6.79 (t, *J* = 7.6 Hz, 2H), 6.72 (d, *J* = 7.2 Hz, 1H), 6.51 (d, *J* = 7.8 Hz, 2H). ^{13}C NMR (101 MHz, $CDCl_3$) δ 182.83, 182.48, 137.27, 134.86, 132.76, 132.39, 131.55, 130.45, 127.82, 127.45, 127.19, 127.10, 126.80, 126.50, 126.15, 125.90, 124.02, 123.82, 122.48, 121.74, 109.98. HRMS calcd. For $C_{22}H_{16}NO_2^+$ $(M+H)^+$ 326.1185 found: 326.1181.

2-(phenylamino)naphthalene-1,4-dione (5a)[3]

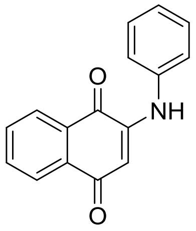

Red solid, 91% yield; mp 182-184 °C. ^{1}H NMR (400 MHz, DMSO-*d*$_6$) δ 9.21 (s, 1H), 8.03 (d, *J* = 7.5 Hz, 1H), 7.92 (d, *J* = 7.5 Hz, 1H), 7.83 (t, *J* = 7.0 Hz, 1H), 7.75 (t, *J* = 7.0 Hz, 1H), 7.45 – 7.35 (m, 4H), 7.20 (t, *J* = 7.2 Hz, 1H), 6.08 (s, 1H). ^{13}C NMR (101 MHz, DMSO-*d*$_6$) δ 183.01, 181.99, 146.61, 138.48, 135.32, 133.06, 132.99, 130.84, 129.73, 126.55, 125.70, 124.13, 102.35.

2-(p-tolylamino)naphthalene-1,4-dione (5b)[4]

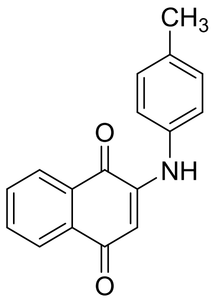

Red solid, 89% yield; mp 197-199 °C.^{1}H NMR (400 MHz, $CDCl_3$) δ 8.07 – 8.02 (m, 2H), 7.69 (t, *J* = 7.5 Hz, 1H), 7.61 (d, *J* = 7.7 Hz, 1H), 7.16 (d, *J* = 8.2 Hz, 3H), 7.10 (d, *J* = 8.2 Hz, 2H), 6.29 (s, 1H), 2.30 (s, 3H). ^{13}C NMR (101 MHz, $CDCl_3$) δ 182.10, 181.44, 144.90, 135.46, 135.17, 134.87, 132.24, 130.22, 128.72, 126.47, 125.81, 123.85, 122.71, 122.52, 103.01, 20.98.

2-((4-(methylthio)phenyl)amino)naphthalene-1,4-dione (5c)[5]

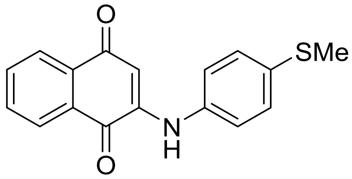

Red solid, 83% yield; mp 166-168 °C. ^{1}H NMR (400 MHz, $CDCl_3$) δ 7.95 (d, *J* = 7.7 Hz, 2H), 7.60 (t, *J* = 7.6 Hz, 1H), 7.51 (t, *J* = 7.5 Hz, 1H), 7.40 (s, 1H), 7.14 (t, *J* = 6.8

Hz, 2H), 7.05 (d, *J* = 8.5 Hz, 2H), 6.20 (s, 1H), 2.35 (s, 3H). ^{13}C NMR (101 MHz, $CDCl_3$) δ 183.75, 181.95, 144.62, 135.79, 134.90, 134.61, 134.27, 133.19, 132.31, 130.30, 127.85, 126.49, 126.14, 125.95, 123.09, 103.34, 16.16.

2-((3-methoxyphenyl)amino)naphthalene-1,4-dione (5d)[6]

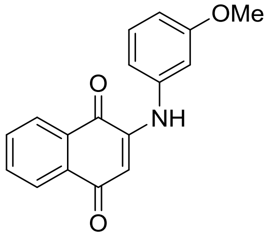

Red solid, 76% yield; mp 163-165 °C. ^{1}H NMR (400 MHz, $CDCl_3$) δ 8.11 (ddd, *J* = 7.5, 4.2, 1.1 Hz, 2H), 7.75 (dt, *J* = 7.6, 3.7 Hz, 1H), 7.67 (dt, *J* = 7.5, 3.7 Hz, 1H), 7.55 (s, 1H), 7.31 (t, *J* = 8.1 Hz, 1H), 6.87 (dd, *J* = 7.9, 1.8 Hz, 1H), 6.80 (t, *J* = 2.2 Hz, 1H), 6.75 (dd, *J* = 8.3, 2.3 Hz, 1H), 6.45 (s, 1H), 3.82 (s, 3H). ^{13}C NMR (101 MHz, $CDCl_3$) δ 183.92, 182.01, 160.60, 144.55, 138.59, 134.90, 133.17, 132.34, 130.44, 130.32, 126.52, 126.15, 114.75, 110.97, 108.46, 103.80, 55.43.

2-((3-chlorophenyl)amino)naphthalene-1,4-dione (5e)[4]

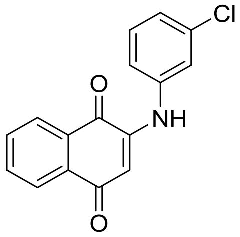

Red solid, 72% yield; mp 151-153 °C. ^{1}H NMR (400 MHz, DMSO-d_6) δ 9.25 (s, 1H), 8.04 (d, *J* = 7.4 Hz, 1H), 7.94 (d, *J* = 7.4 Hz, 1H), 7.82 (t, *J* = 7.3 Hz, 1H), 7.75 (t, *J* = 7.3 Hz, 1H), 7.45 – 7.33 (m, 3H), 7.21 (d, *J* = 7.8 Hz, 1H), 6.16 (s, 1H). ^{13}C NMR (101 MHz, DMSO-d_6) δ 183.29, 181.54, 145.93, 140.26, 135.22, 133.07, 131.16, 130.29, 126.54, 125.73, 125.14, 124.58, 123.53, 122.14, 103.56.

2-(o-tolylamino)naphthalene-1,4-dione (5f)[7]

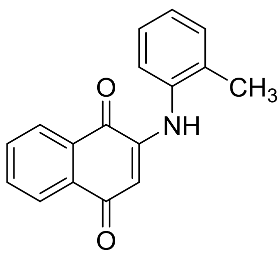

Red solid, 77% yield; mp 141-143 °C. ^{1}H NMR (400 MHz, DMSO-d_6) δ 8.98 (s, 1H), 8.00 (dd, *J* = 7.6, 0.9 Hz, 1H), 7.88 (dd, *J* = 7.6, 0.9 Hz, 1H), 7.79 (td, *J* = 7.5, 1.3 Hz, 1H), 7.71 (td, *J* = 7.5, 1.3 Hz, 1H), 7.36 – 7.19 (m, 4H), 5.31 (s, 1H), 2.18 (s, 3H). ^{13}C NMR (101 MHz, DMSO-d_6)) δ 182.47, 181.84, 148.13, 136.55, 135.23, 134.96,

133.20, 132.84, 131.46, 130.85, 127.58, 127.31, 127.18, 126.40, 125.72, 101.72, 17.85.

2-((2-hydroxyphenyl)amino)naphthalene-1,4-dione (5g)[8]

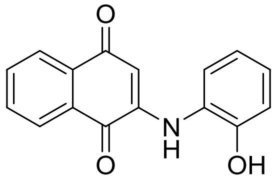

Red solid, 62% yield; mp 193-195 °C. ^{1}H NMR (400 MHz, DMSO-d_6) δ 9.31 (s, 1H), 8.02 (d, *J* = 7.4 Hz, 1H), 7.90 (dd, *J* = 11.6, 8.8 Hz, 3H), 7.82 (t, *J* = 7.2 Hz, 1H), 7.75 (t, *J* = 7.3 Hz, 1H), 7.43 (d, *J* = 8.4 Hz, 2H), 7.32 (s, 1H), 6.23 (s, 1H). ^{13}C NMR (101 MHz, DMSO-d_6) δ 183.29, 181.86, 167.65, 145.80, 141.40, 135.35, 133.22, 132.85, 132.63, 130.83, 130.68, 129.12, 126.63, 125.74, 122.75, 109.98.

2-((2-chlorophenyl)amino)naphthalene-1,4-dione (5h)[9]

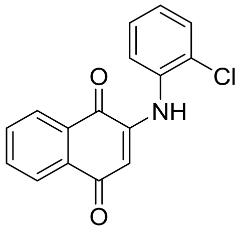

Red solid, 65% yield; mp 143-145 °C. ^{1}H NMR (400 MHz, DMSO-d_6) δ 9.03 (s, 1H), 8.02 (d, *J* = 7.5 Hz, 1H), 7.90 (d, *J* = 7.5 Hz, 1H), 7.82 (td, *J* = 7.5, 1.2 Hz, 1H), 7.75 (td, *J* = 7.5, 1.2 Hz, 1H), 7.60 (d, *J* = 7.9 Hz, 1H), 7.51 – 7.38 (m, 2H), 7.38 – 7.29 (m, 1H), 5.46 (s, 1H). ^{13}C NMR (101 MHz, DMSO-d_6) δ 182.81, 181.57, 146.91, 135.41, 135.24, 133.18, 132.90, 130.72, 130.68, 130.12, 128.81, 128.72, 128.32, 126.54, 125.83, 103.41.

2-(naphthalen-1-ylamino)naphthalene-1,4-dione (5i)[4]

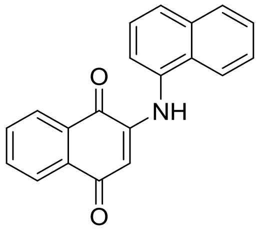

Red solid, 83% yield; mp 157-159 °C. ^{1}H NMR (400 MHz, DMSO-d_6) δ 9.50 (s, 1H), 8.08 (dd, *J* = 7.5, 1.1 Hz, 1H), 8.03 – 7.98 (m, 1H), 7.94 (d, *J* = 8.2 Hz, 1H), 7.91 – 7.86 (m, 2H), 7.79 (dtd, *J* = 21.3, 7.4, 1.4 Hz, 2H), 7.61 – 7.48 (m, 4H), 5.22 (s, 1H). ^{13}C NMR (101 MHz, DMSO-d_6) δ 182.57, 181.79, 149.31, 135.21, 134.47, 134.33, 133.17, 132.96, 131.02, 129.35, 128.84, 127.95, 127.06, 127.00, 126.49, 126.37, 125.73, 125.03, 123.63, 102.60.

2-((3,5-di-tert-butylphenyl)amino)naphthalene-1,4-dione (5j)

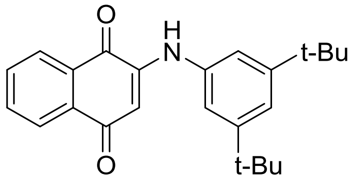

Red solid, 78% yield; mp 142-144 °C. ^{1}H NMR (400 MHz, $CDCl_3$) δ 8.11 (d, *J* = 7.8 Hz, 2H), 7.74 (td, *J* = 7.6, 1.1 Hz, 1H), 7.68 – 7.58 (m, 2H), 7.28 (d, *J* = 1.5 Hz, 1H), 7.10 (d, *J* = 1.5 Hz, 2H), 6.33 (s, 1H), 1.34 (s, 19H). ^{13}C NMR (101 MHz, $CDCl_3$) δ 183.77, 182.23, 152.57, 145.21, 136.73, 134.83, 133.37, 132.17, 130.37, 126.44, 126.13, 120.04, 117.28, 103.01, 35.02, 31.37. HRMS calcd. For C22H16NO2$^+$ $(M+H)^+$ 362.2117 found: 362.2120.

2-(pyridin-2-ylamino)naphthalene-1,4-dione (5k)[10]

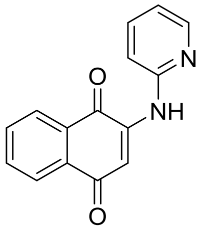

Red solid, 71% yield; mp 201-203 °C. ^{1}H NMR (400 MHz, DMSO-d_6) δ 9.45 (s, 1H), 8.38 (dd, *J* = 4.8, 1.2 Hz, 1H), 8.04 (dd, *J* = 7.6, 6.8 Hz, 2H), 7.94 (d, *J* = 7.4 Hz, 1H), 7.84 (td, *J* = 7.4, 1.1 Hz, 1H), 7.80 – 7.72 (m, 2H), 7.55 (d, *J* = 8.3 Hz, 1H), 7.03 (dd, *J* = 6.7, 5.2 Hz, 1H). ^{13}C NMR (101 MHz, DMSO-d_6) δ 184.73, 181.84, 154.22, 147.73, 143.13, 138.30, 135.23, 133.40, 132.46, 130.80, 126.70, 125.69, 118.47, 115.68, 110.51.

2-(propylamino)naphthalene-1,4-dione (5l)

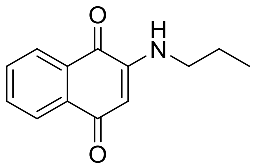

Yellow solid, 72% yield; mp 165-167 °C. ^{1}H NMR (400 MHz, DMSO-d_6) δ 7.93 (dd, *J* = 15.0, 7.6 Hz, 2H), 7.80 (dd, *J* = 10.7, 4.3 Hz, 1H), 7.72 – 7.65 (m, 1H), 7.54 (t, *J* = 5.8 Hz, 1H), 5.64 (s, 1H), 3.11 (dd, *J* = 13.9, 6.6 Hz, 2H), 1.57 (dd, *J* = 14.5, 7.3 Hz, 2H), 0.88 (t, *J* = 7.4 Hz, 3H). ^{13}C NMR (101 MHz, DMSO-d_6) δ 181.72, 181.63, 148.99, 135.24, 132.52, 132.25, 130.35, 126.30, 125.73, 99.62, 43.97, 21.14, 11.84. HRMS calcd. For $C_{22}H_{16}NO_2^+$ $(M+H)^+$ 216.1019 found: 216.1025.

5-methyl-6-phenyl-5,6-dihydrobenzo[f]indolo[2,3-b]indole-7,12-dione (6a)

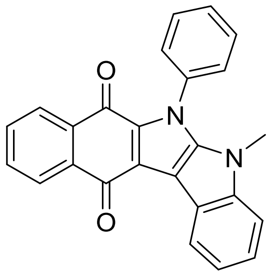

Red solid, 81 % yield; mp 275-277 °C. ^{1}H NMR (400 MHz, $CDCl_3$) δ 8.35 (d, *J* = 7.3 Hz, 1H), 8.14 (d, *J* = 5.9 Hz, 1H), 7.94 (d, *J* = 5.5 Hz, 1H), 7.60 (d, *J* = 15.9 Hz, 7H), 7.36 – 7.23 (m, 3H), 7.17 (d, *J* = 7.6 Hz, 1H), 3.24 (s, 3H). ^{13}C NMR (101 MHz, $CDCl_3$) δ 181.88, 174.08, 143.50, 136.44, 134.64, 133.08, 132.28, 129.81, 129.49, 127.94, 125.99, 124.18, 122.63, 121.09, 119.90, 109.28, 30.09. HRMS calcd. For $C_{25}H_{16}N_2O_2Na^+$ $(M+Na)^+$ 399.1104 found 399.1101.

2-chloro-5-methyl-6-phenyl-5,6-dihydrobenzo[f]indolo[2,3-b]indole-7,12-dione (6b)

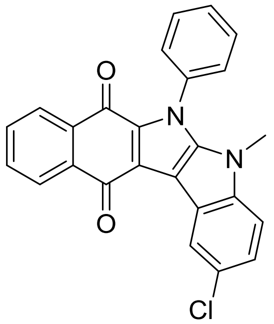

Red solid, 62 % yield; mp 261-263 °C. ^{1}H NMR (400 MHz, $CDCl_3$) δ 8.29 – 8.28 (m, 1H), 8.14 – 8.12 (m, 1H), 7.92 (dd, *J* = 6.3, 1.2 Hz, 1H), 7.63 – 7.56 (m, 7H), 7.22 (d, *J* = 2.2 Hz, 1H), 7.08 (s, 1H), 3.25 (s, 3H). ^{13}C NMR (101 MHz, $CDCl_3$) δ 181.53, 174.30, 141.68, 136.21, 134.43, 133.12, 132.98, 132.47, 129.91, 129.52, 127.93, 126.55, 126.06, 126.03, 123.98, 122.02, 120.92, 110.18, 77.29, 76.97, 76.65, 30.23. HRMS calcd. For $C_{25}H_{15}ClN_2NaO_2^+$ $(M+Na)^+$ 433.0714 found: 433.0719.

3-fluoro-5-methyl-6-phenyl-5,6-dihydrobenzo[f]indolo[2,3-b]indole-7,12-dione (6c)

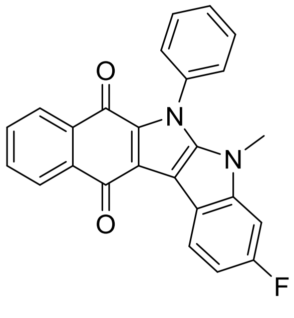

Red solid, 56 % yield; mp 281-283 °C. ^{1}H NMR (400 MHz, $CDCl_3$) δ 8.28 (dd, *J* = 8.6, 5.6 Hz, 1H), 8.17 – 8.14 (m, 1H), 7.99 – 7.94 (m, 1H), 7.63 – 7.57 (m, 7H), 7.03

– 6.98 (m, 1H), 6.90 (dd, *J* = 9.6, 2.2 Hz, 1H), 3.23 (s, 3H). ^{13}C NMR (101 MHz, $CDCl_3$) δ 181.81, 174.18, 136.27, 134.64, 133.13, 133.08, 132.35, 129.89, 129.52, 127.88, 126.07, 126.01, 123.56, 123.46, 109.08, 108.85, 97.03, 96.75, 77.29, 77.17, 76.97, 76.65, 30.31. HRMS calcd. For $C_{25}H_{16}FN_2O_2^+$ $(M+H)^+$ 395.1190 found: 395.1195.

7. References

1. L. Zhang, C. Peng, D. Zhao, Y. Wang, H.-J. Fu, Q. Shen and J.-X. Li, Cu (ii)-catalyzed C–H (SP 3) oxidation and C–N cleavage: base-switched methylenation and formylation using tetramethylethylenediamine as a carbon source, *Chem. Commun*., 2012, **48**, 5928-5930.
2. Y. Dong, H. Zhang, J. Yang, S. He, Z.-C. Shi, X.-M. Zhang and J.-Y. Wang, $B(C_6F_5)_3$-Catalyzed C–C Coupling of 1,4-Naphthoquinones with the C-3 Position of Indole Derivatives in Water, *ACS omega*., 2019, **4**, 21567-21577.
3. I. Sieveking, P. Thomas, J. C. Estévez, N. Quiñones, M. A. Cuéllar, J. Villena, C. Espinosa-Bustos, A. Fierro, R. A. Tapia, J. D. Maya, R. López-Muñoz, B. K. Cassels, R. J. Estévez and C. O. Salas, 2-Phenylaminonaphthoquinones and related compounds: Synthesis, trypanocidal and cytotoxic activities, *Bioorg. Med. Chem.*, 2014, **22**, 4609-4620.
4. L. Huang, J. Zhao, S. Guo, C. Zhang and J. Ma, Bodipy derivatives as organic triplet photosensitizers for aerobic photoorganocatalytic oxidative coupling of amines and photooxidation of dihydroxylnaphthalenes, *J. Org. Chem.*, 2013, **78**, 5627-5637.
5. X.-L. Chen, Y. Dong, S. He, R. Zhang, H. Zhang, L. Tang, X.-M. Zhang and J.-Y. Wang, one-pot approach to 2-(N-substituted amino)-1, 4-naphthoquinones with use of nitro compounds and 1, 4-naphthoquinones in water, *Synlett.*, 2019, **30**, 615-619.
6. C. d. S. Lisboa, V. G. Santos, B. G. Vaz, N. C. de Lucas, M. N. Eberlin and S. J. Garden, C− H Functionalization of 1, 4-Naphthoquinone by Oxidative Coupling with Anilines in the Presence of a Catalytic Quantity of Copper (II) Acetate, *J. Org. Chem.*, 2011, **76**, 5264-5273.
7. U. Sharma, D. Katoch, S. Sood, N. Kumar, B. Singh, A. Thakur and A. Gulati, Synthesis, antibacterial and antifungal activity of 2-amino-1, 4-naphthoquinones using silica-supported perchloric acid ($HClO_4$-SiO_2) as a mild, recyclable and highly efficient heterogeneous catalyst, *Indian, J Chem., Section B*, 2013, **53**,1431-1440.
8. K. R. PR, A. Fernandez, S. P. Laila, B. Arunkumar, C. Sreelakshmi and V. Vishnu, Synthesis, spectral characterization, crystal structure, cytotoxicity and apoptosis—Inducing activity of two derivatives of 2-hydroxy-1, 4-naphthaquinone, *Photodiagn. Photodyn. Ther.*, 2017, **17**, 250-259.
9. B. Liu and S. J. Ji, Facile Synthesis of 2‐Amino‐1, 4‐naphthoquinones catalyzed by Molecular Iodine under Ultrasonic Irradiation, *Syn Commun.*, 2008, **38**, 1201-1211.
10. R. Carceller, J. L. García-Navío, M. L. Izquierdo, J. Alvarez-Builla, M. Fajardo, P. Gómez-Sal and F. Gago, Azinium-N-(2′-azinyl) aminides: synthesis, structure and reactivity, *Tetrahedron.*, 1994, **50**, 4995-5012.

8. Copies of ^{1}H, ^{13}C and ^{19}F NMR spectra for all Compounds

2-(1-methyl-1*H*-indol-3-yl)-3-(phenylamino)naphthalene-1,4-dione (3a)

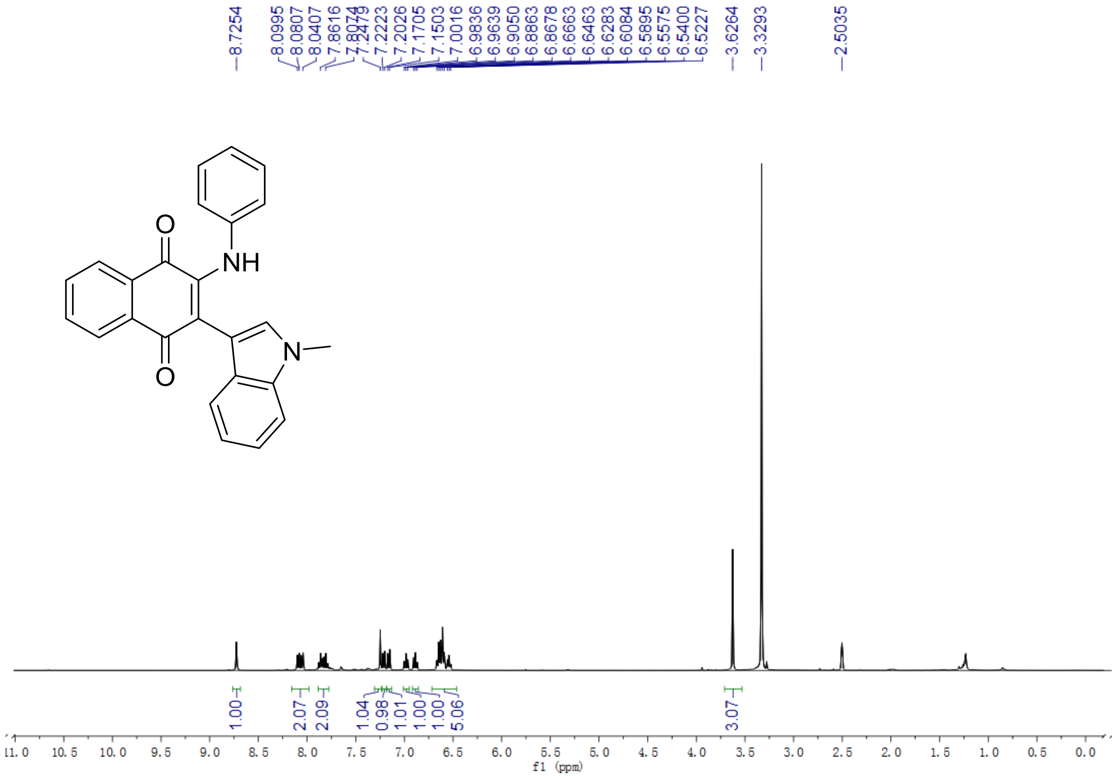

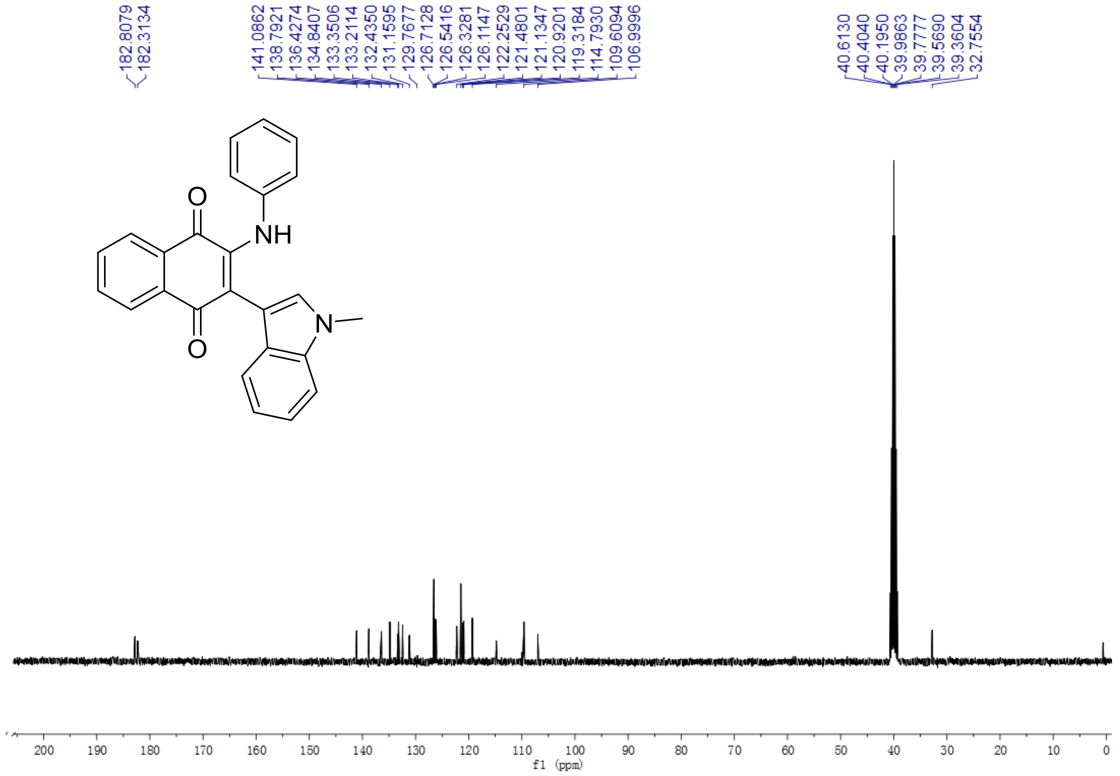

2-(1-methyl-1H-indol-3-yl)-3-(p-tolylamino)naphthalene-1,4-dione (3b)

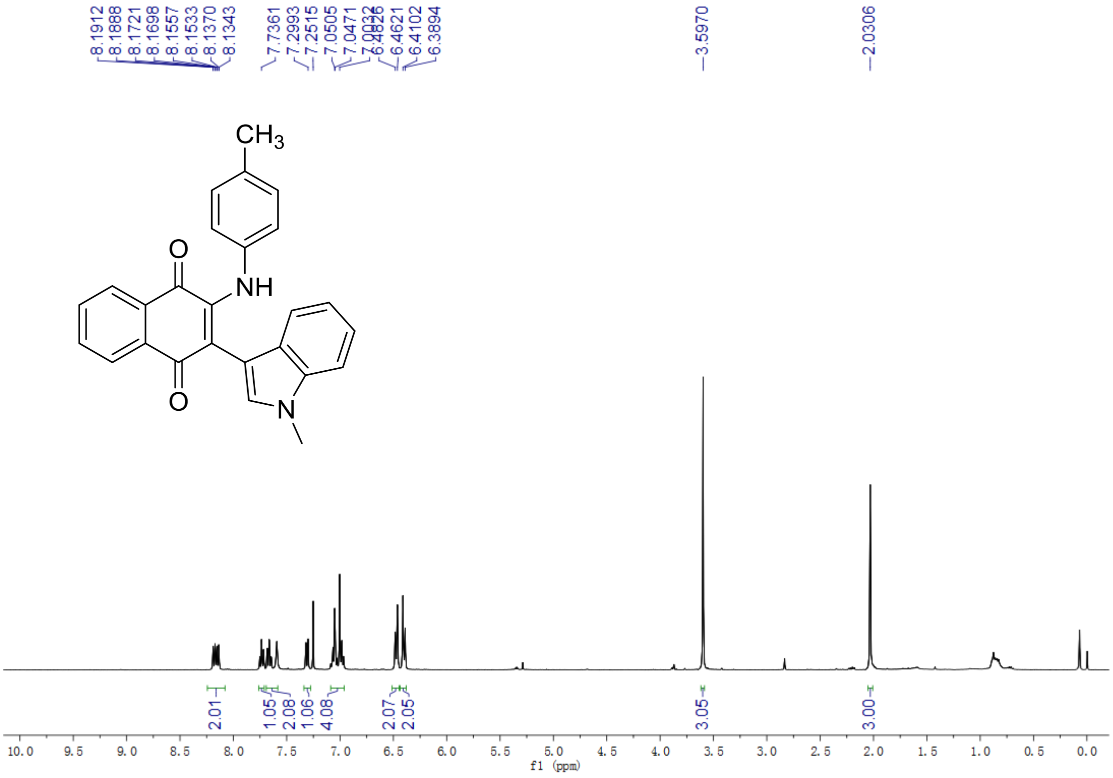

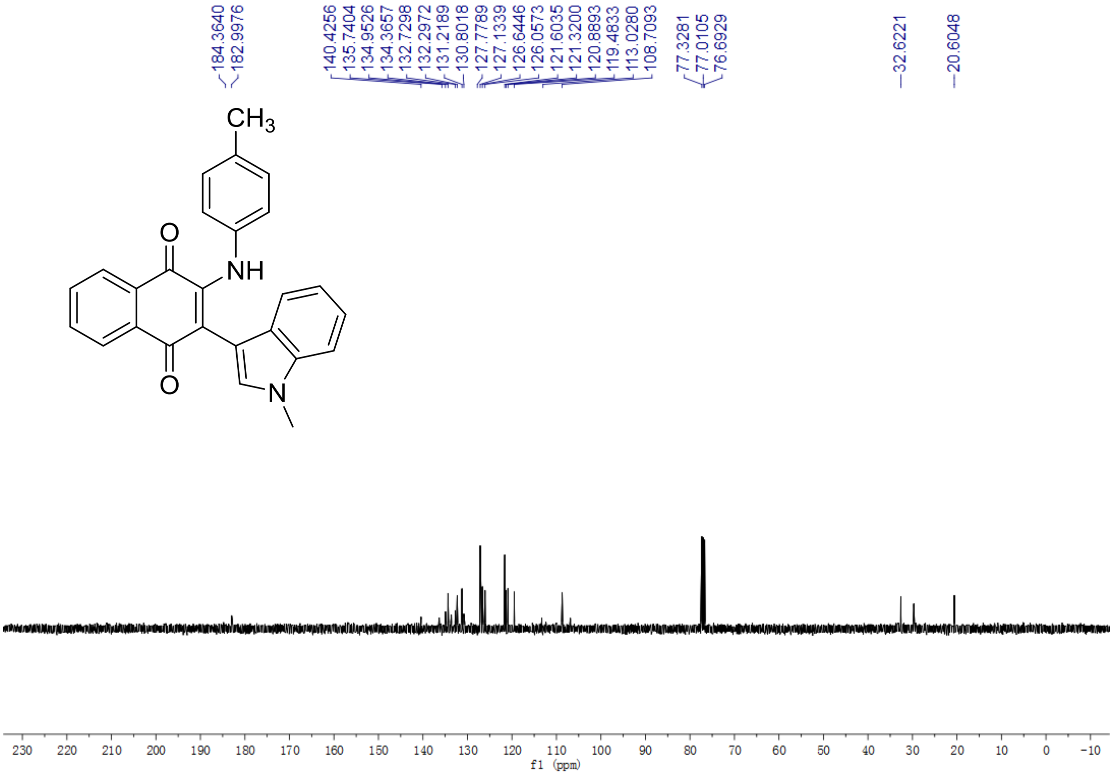

2-((4-chlorophenyl)amino)-3-(1-methyl-1*H*-indol-3-yl)naphthalene-1,4-dione (3c)

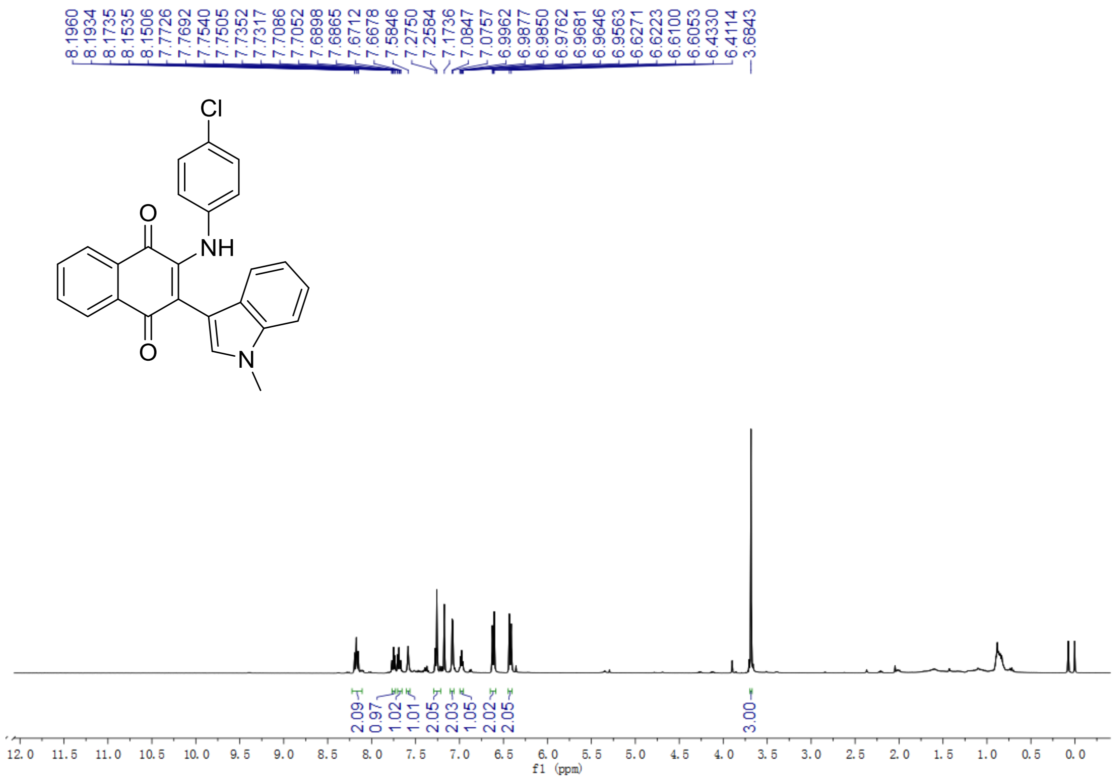

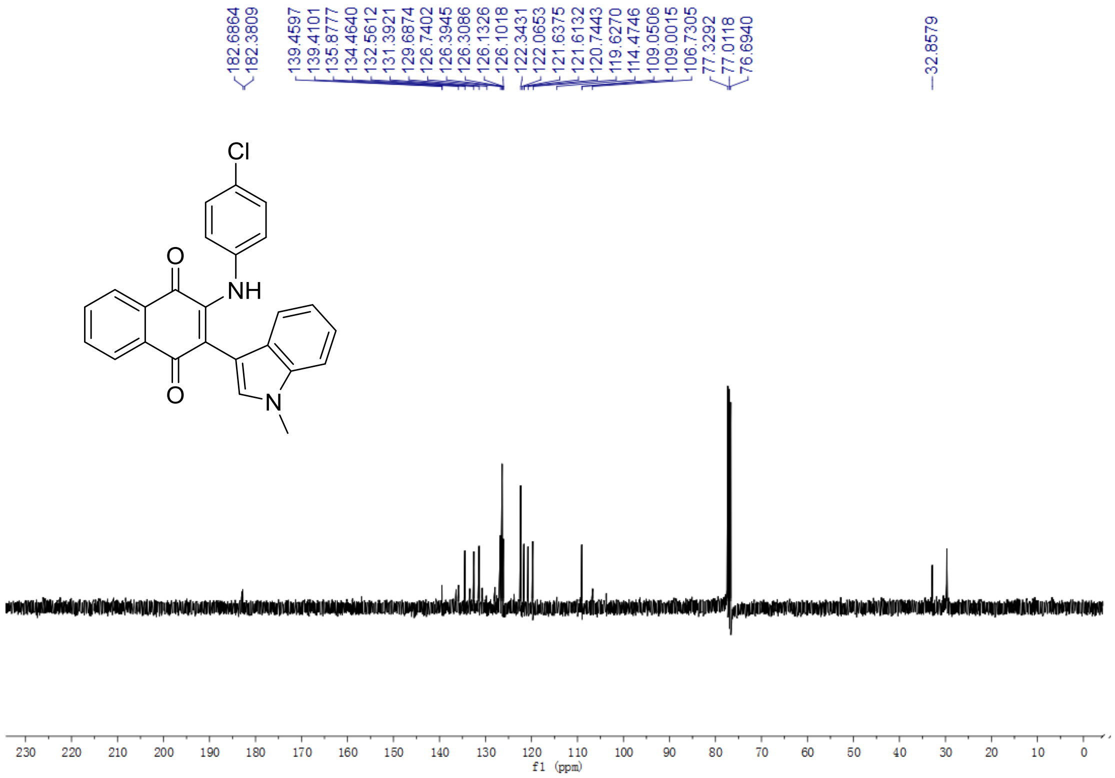

2-((4-bromophenyl)amino)-3-(1-methyl-1*H*-indol-3-yl)naphthalene-1,4-dione (3d)

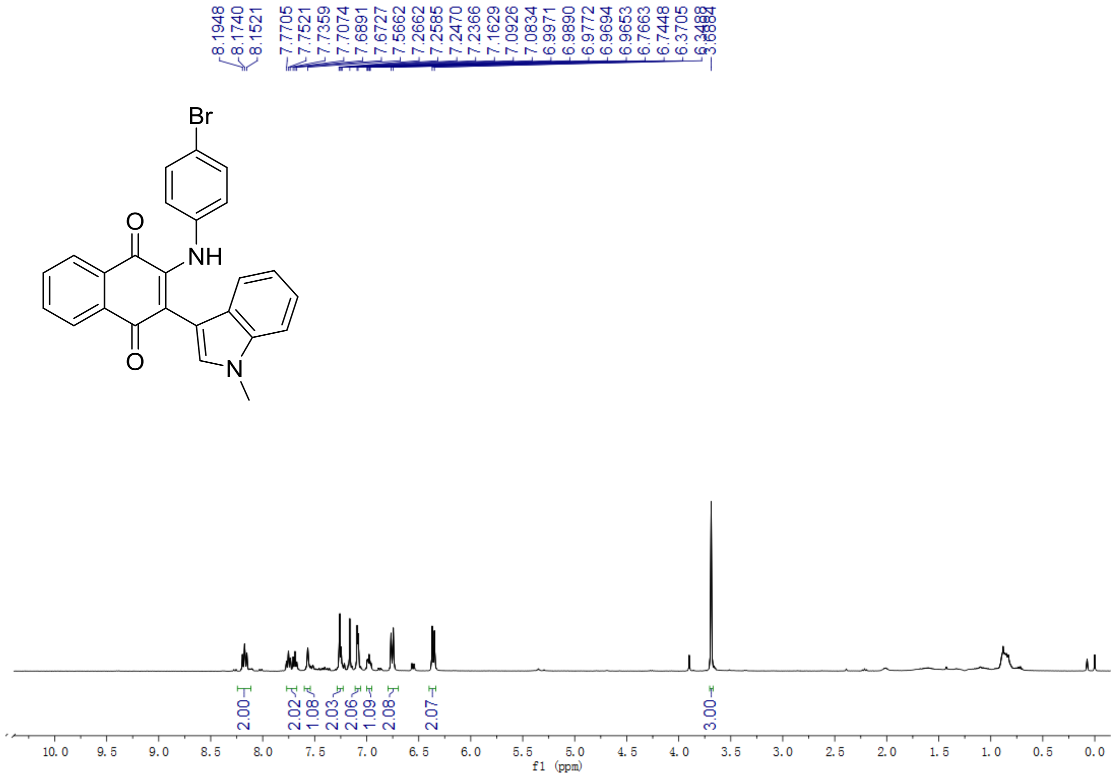

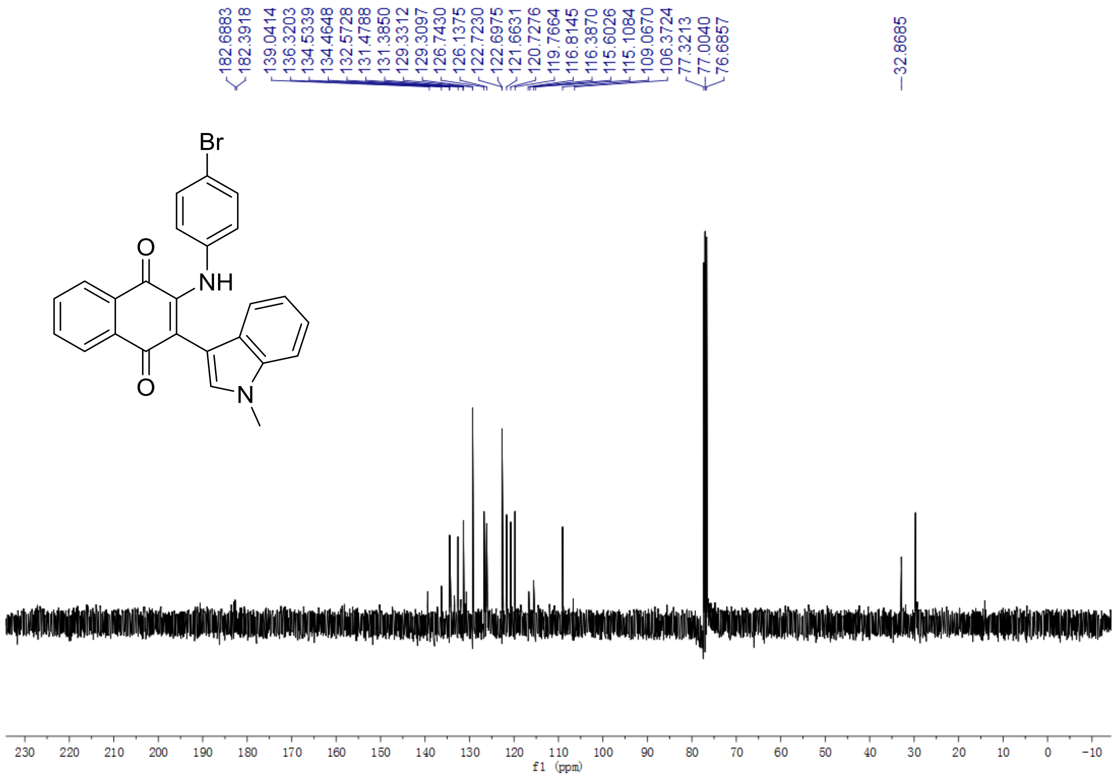

2-((3-chlorophenyl)amino)-3-(1-methyl-1*H*-indol-3-yl)naphthalene-1,4-dione (3e)

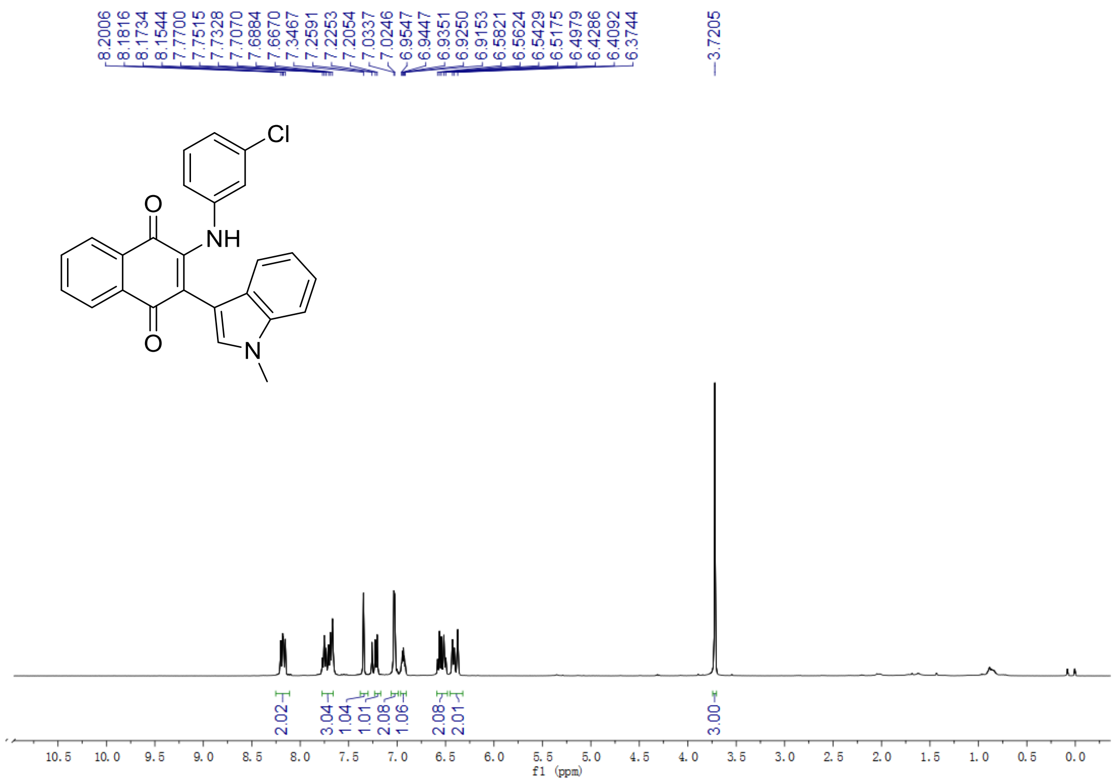

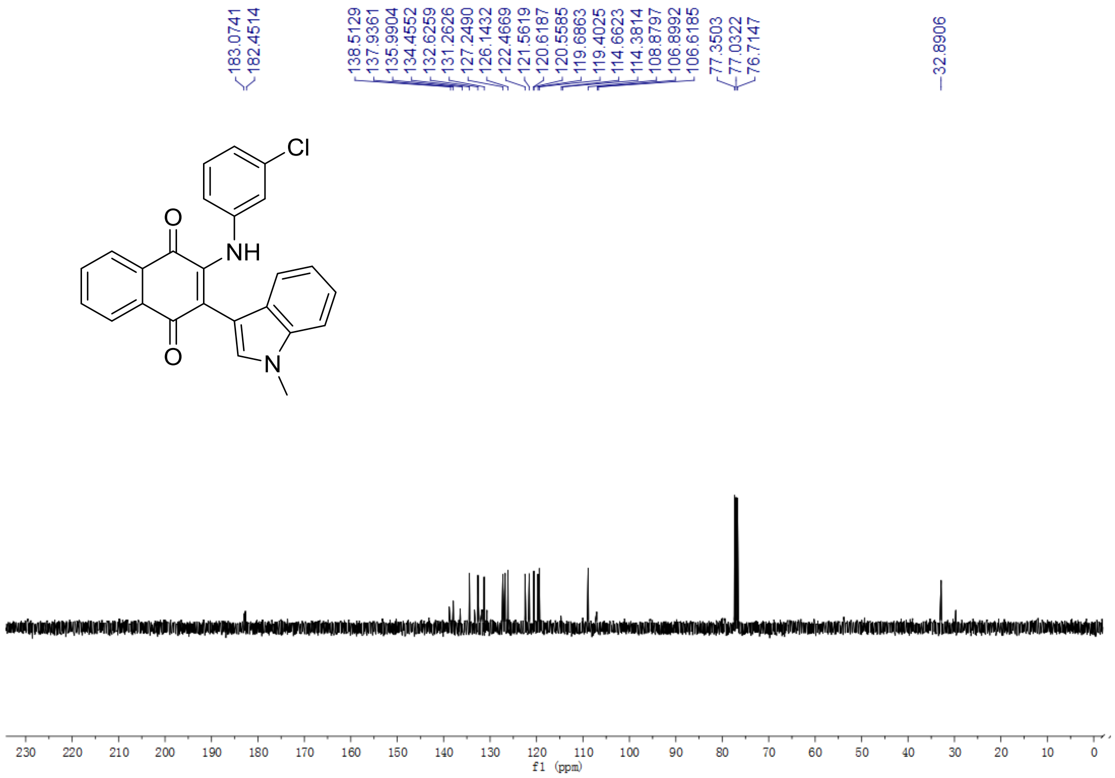

2-(1-methyl-1*H*-indol-3-yl)-3-(o-tolylamino)naphthalene-1,4-dione (3f)

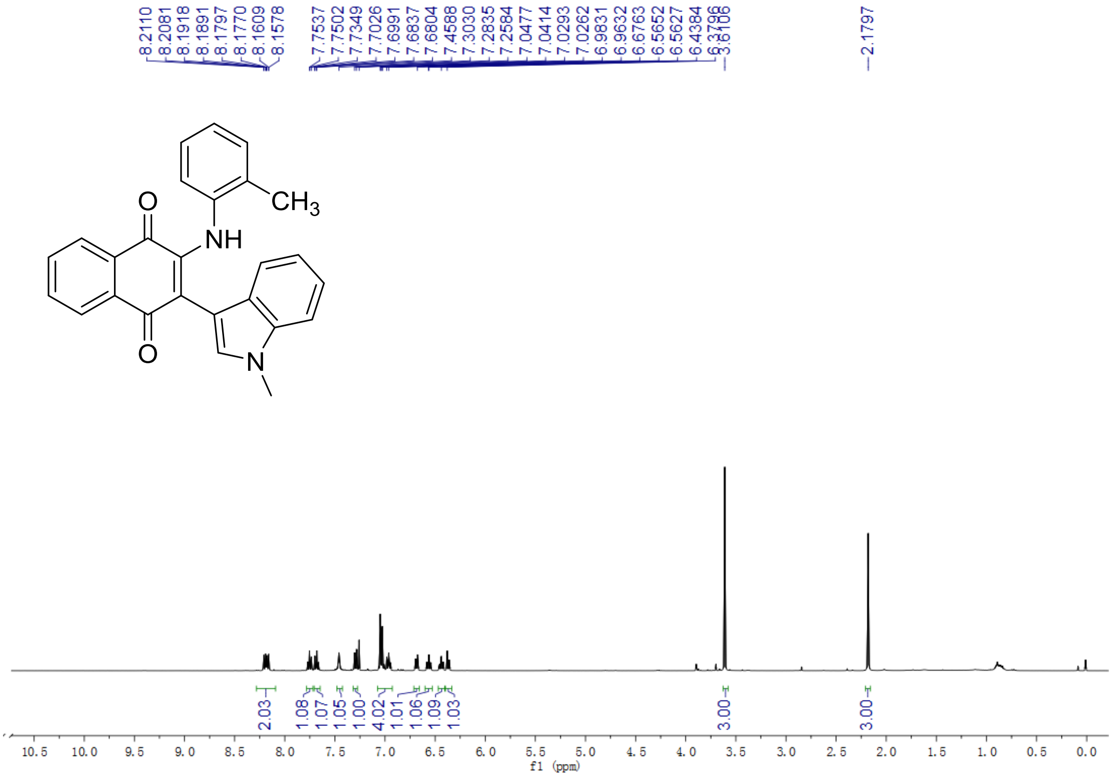

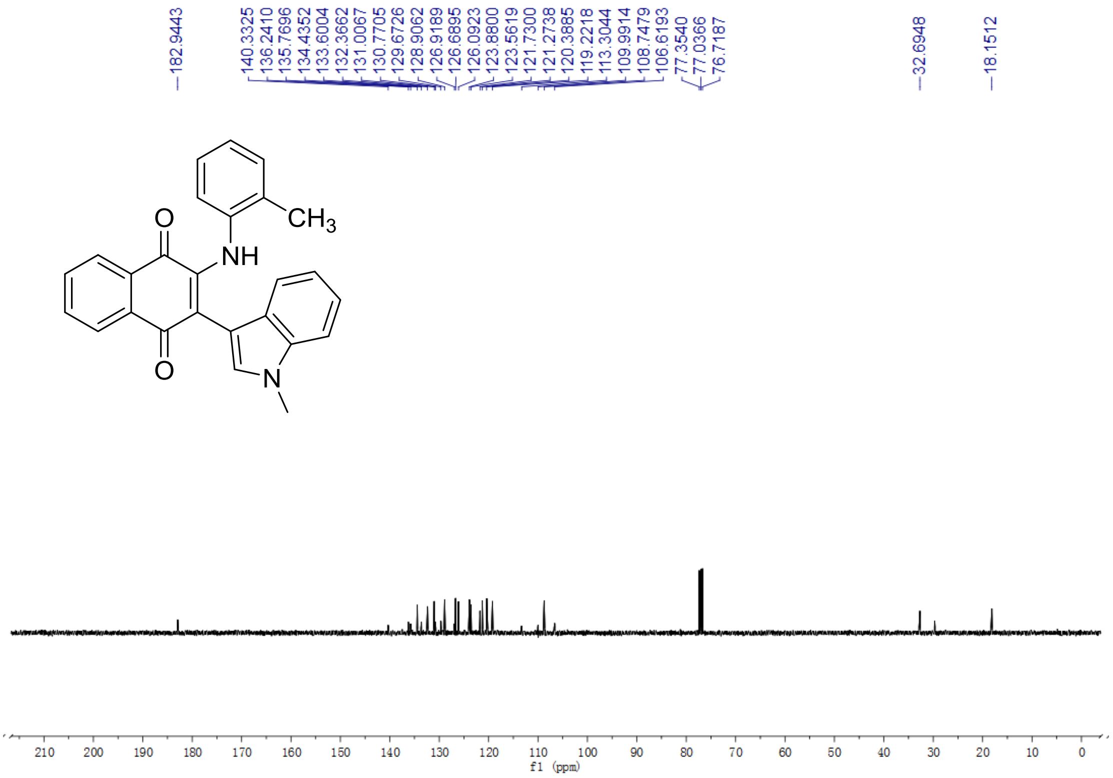

2-((2-methoxyphenyl)amino)-3-(1-methyl-1*H*-indol-3-yl)naphthalene-1,4-dione (3g)

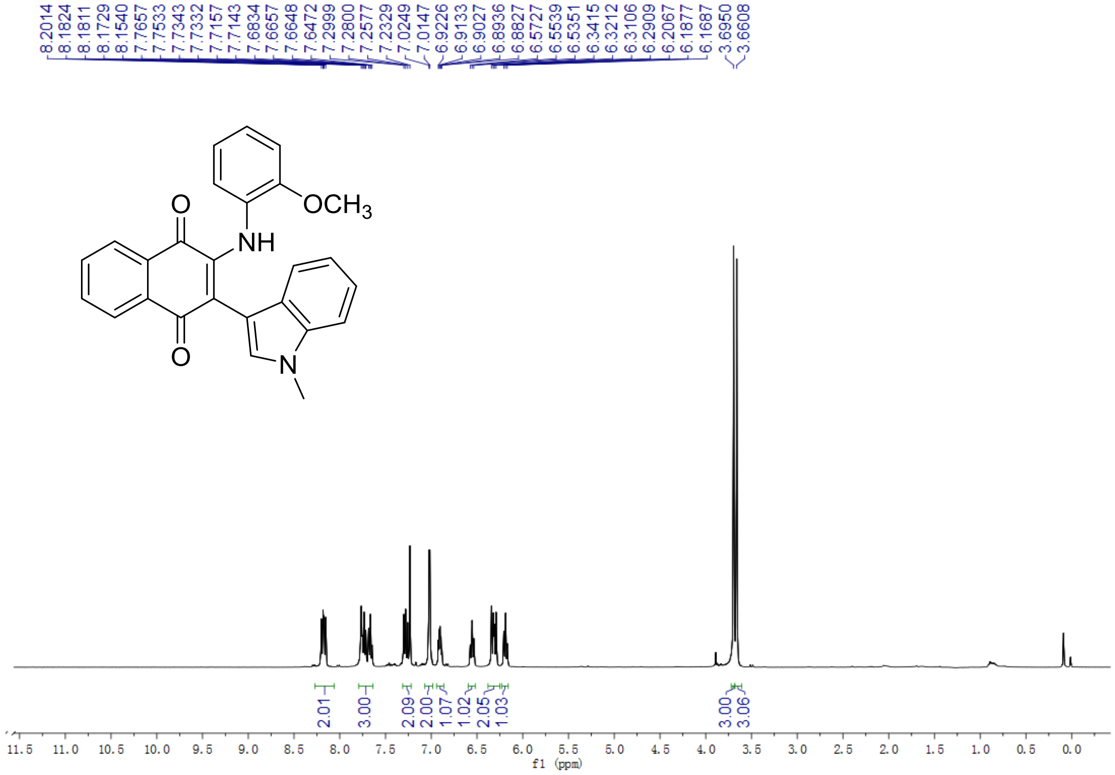

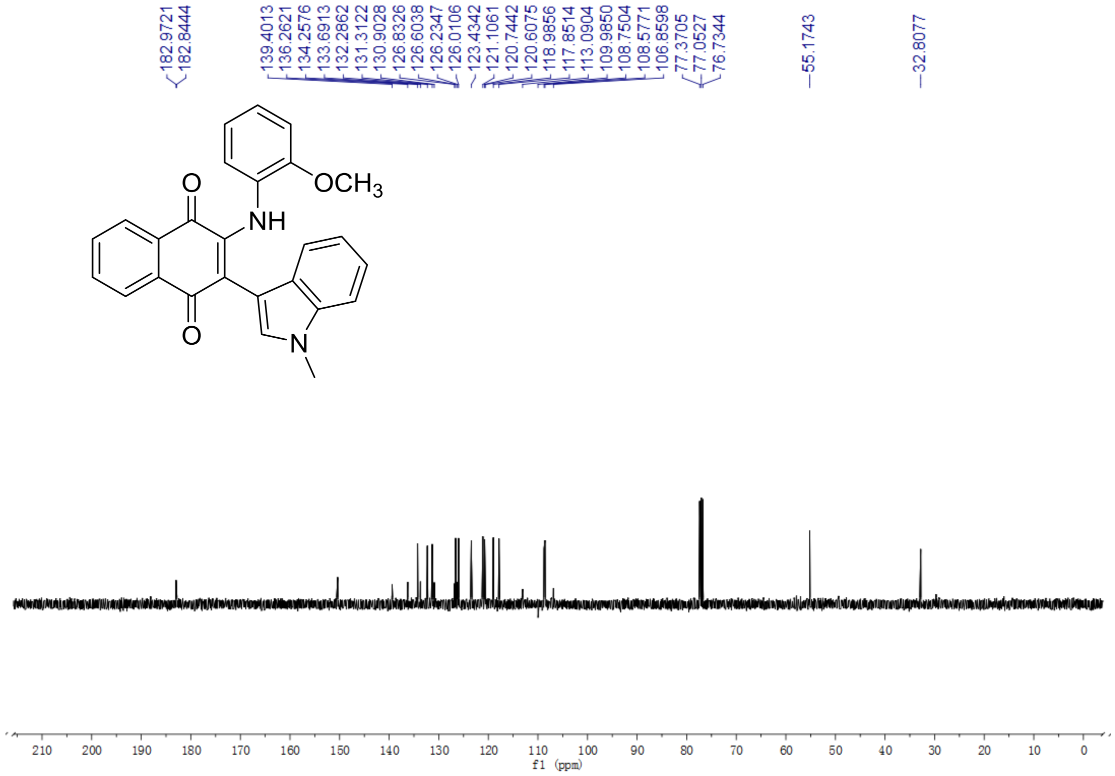

2-((2-chlorophenyl)amino)-3-(1-methyl-1*H*-indol-3-yl)naphthalene-1,4-dione (3h)

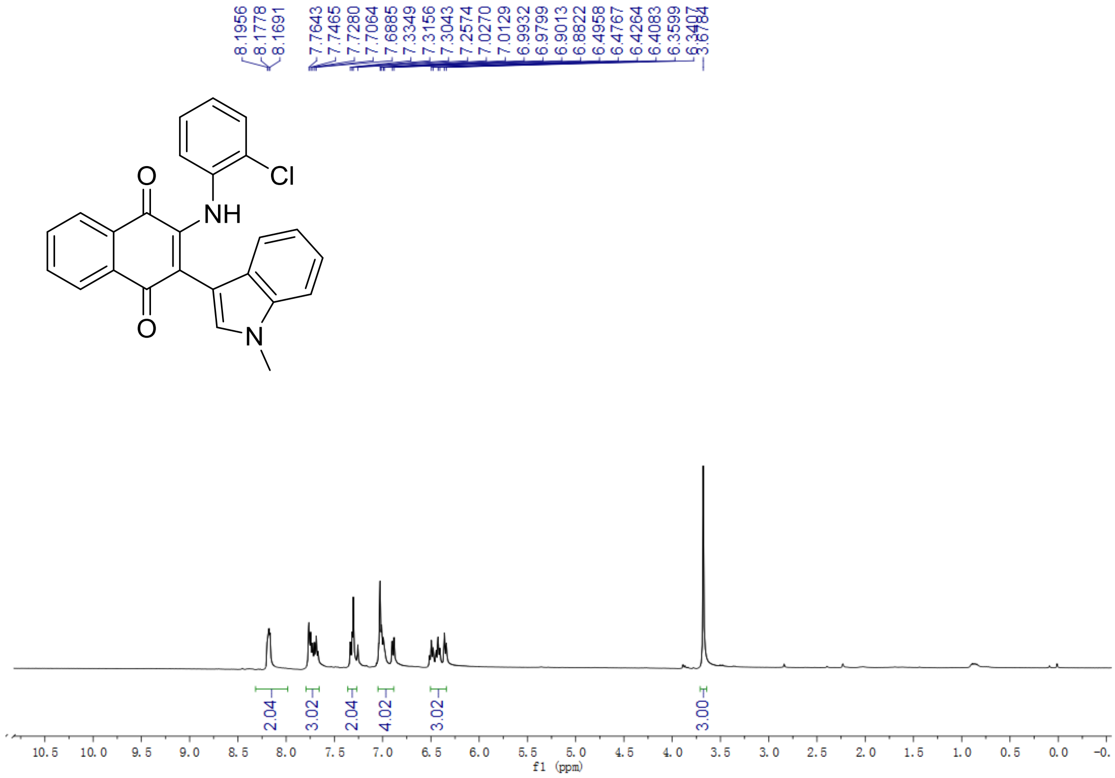

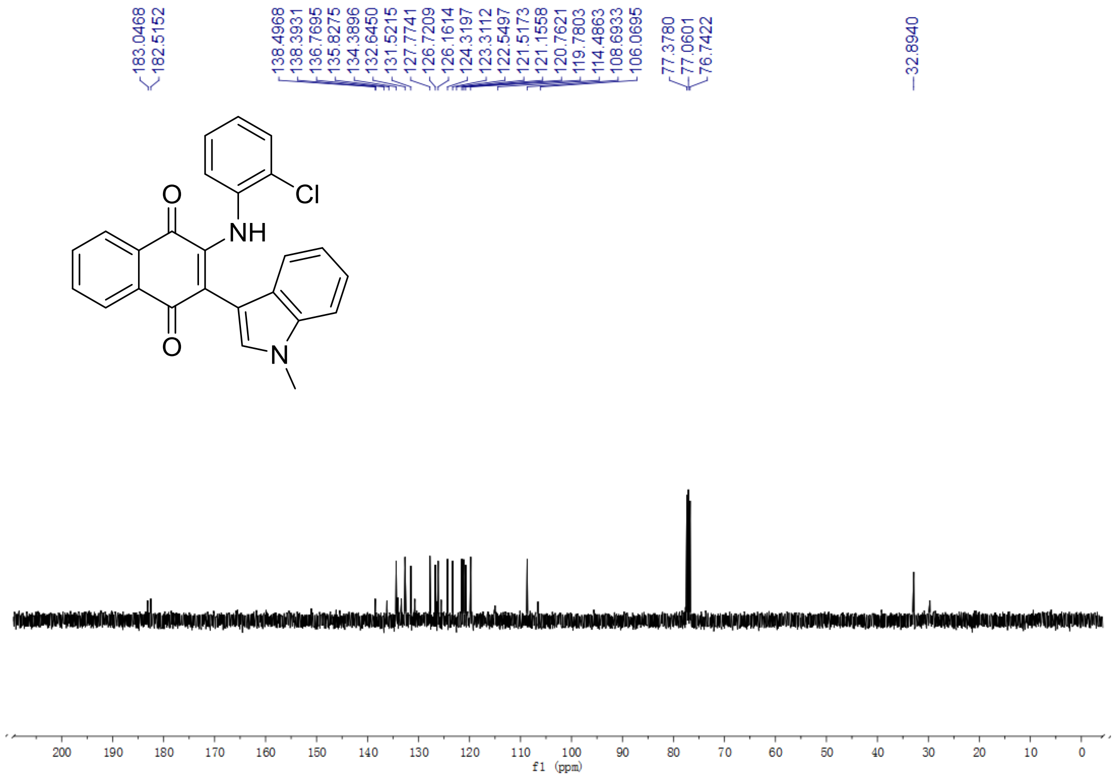

2-(1-methyl-1*H*-indol-3-yl)-3-(pyridin-2-ylamino)naphthalene-1,4-dione (3i)

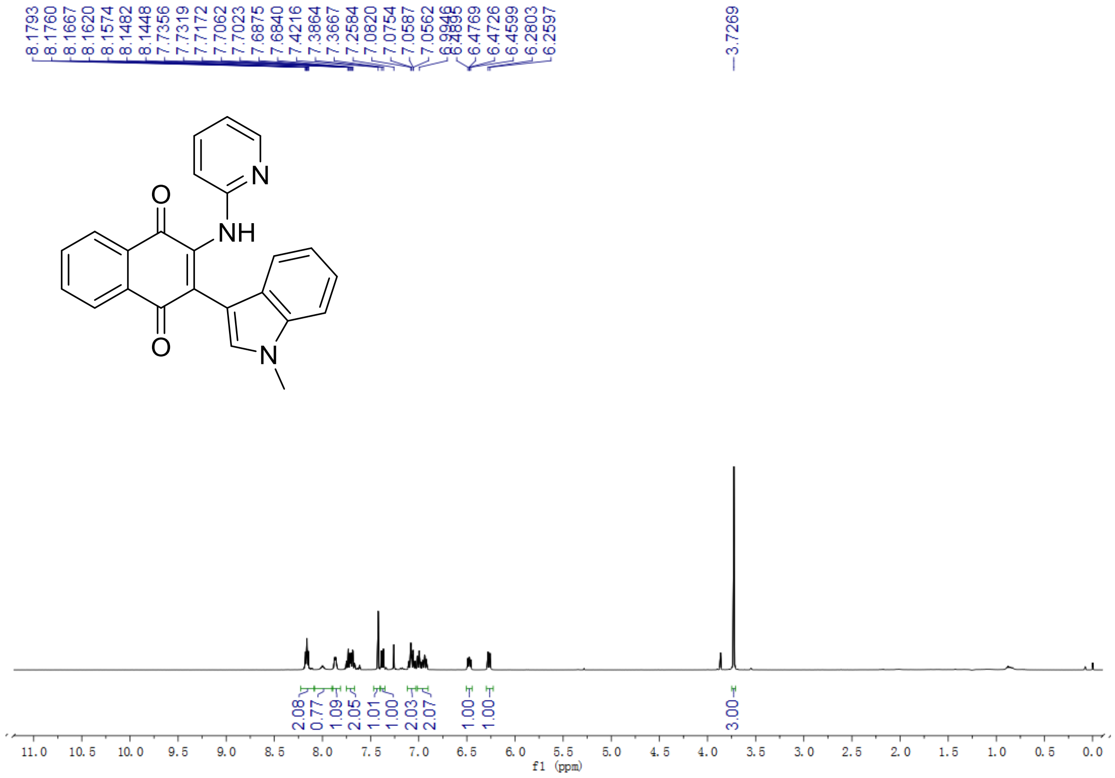

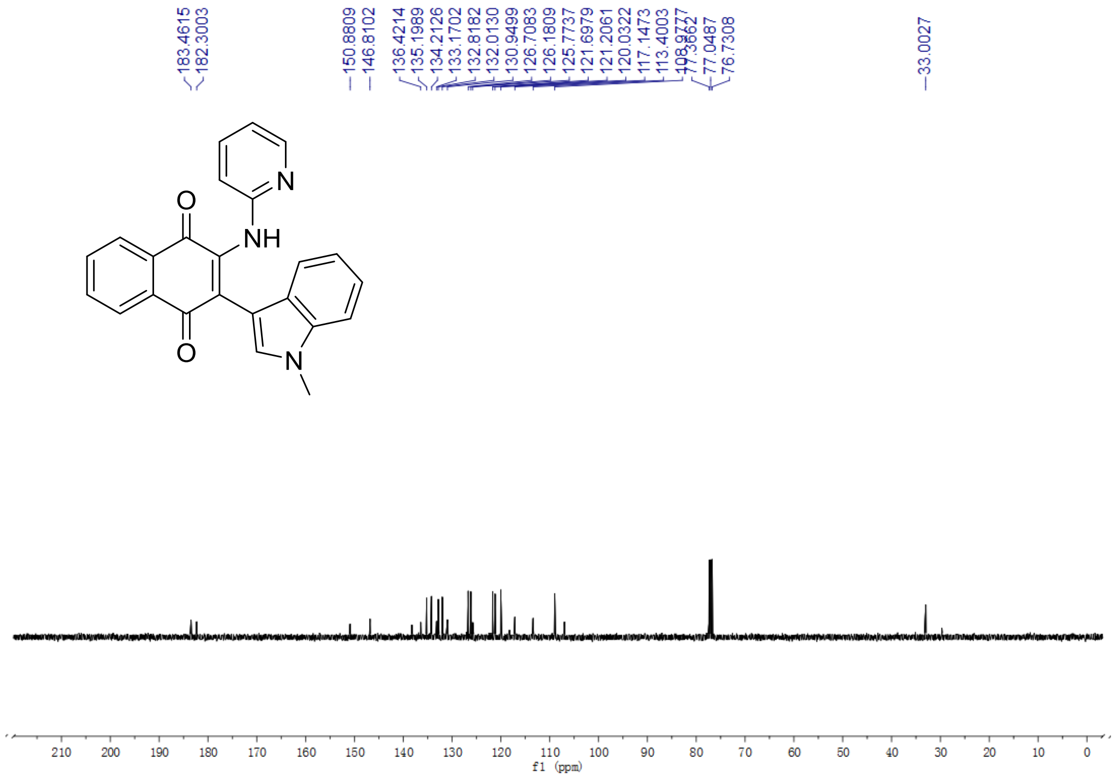

2-(5-chloro-1-methyl-1H-indol-3-yl)-3-(phenylamino)naphthalene-1,4-dione (3j)

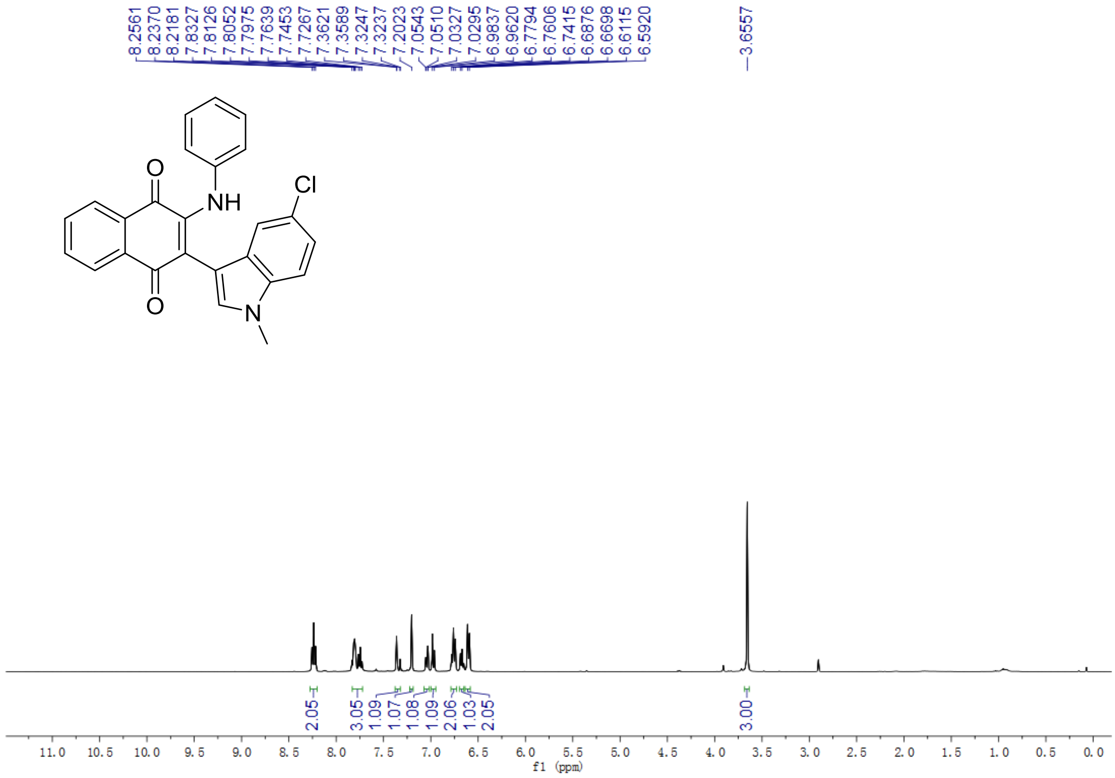

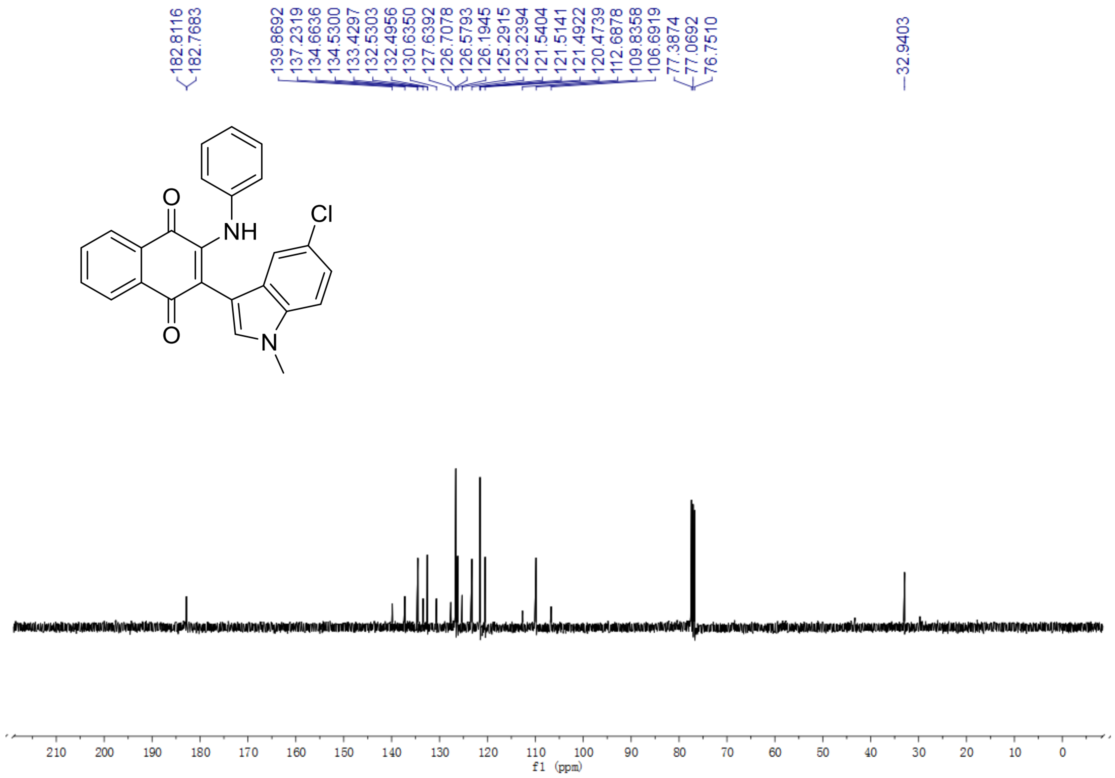

2-(6-fluoro-1-methyl-1H-indol-3-yl)-3-(phenylamino)naphthalene-1,4-dione (3k)

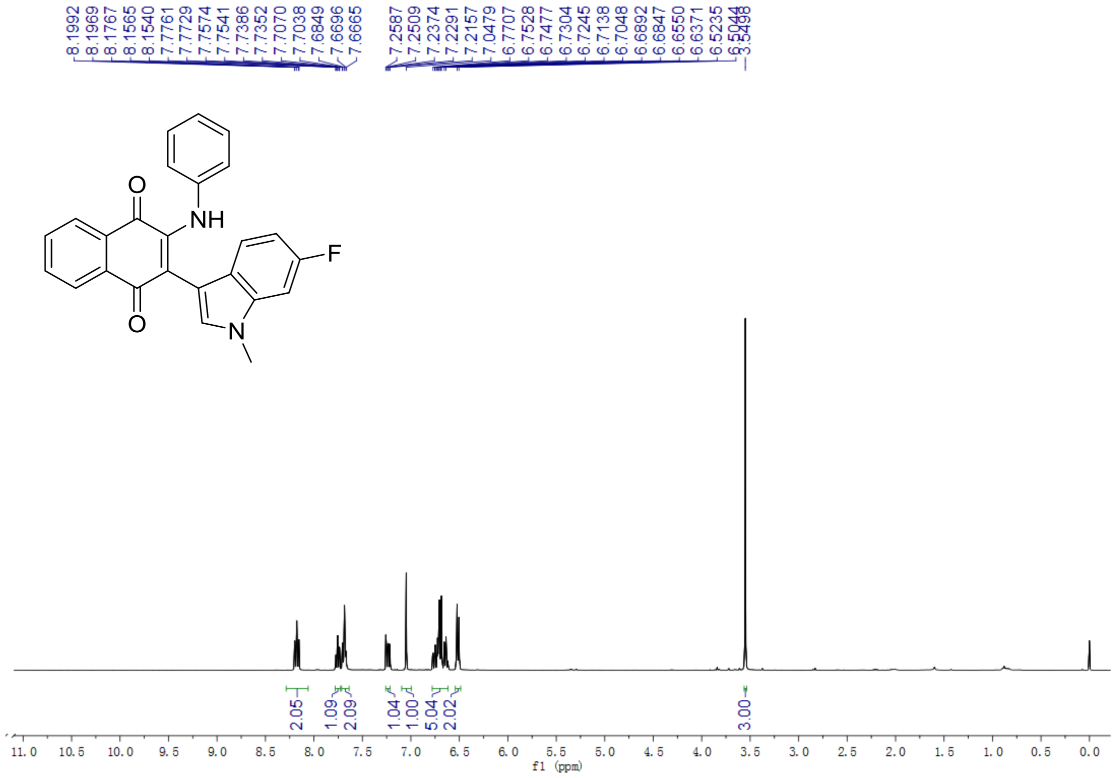

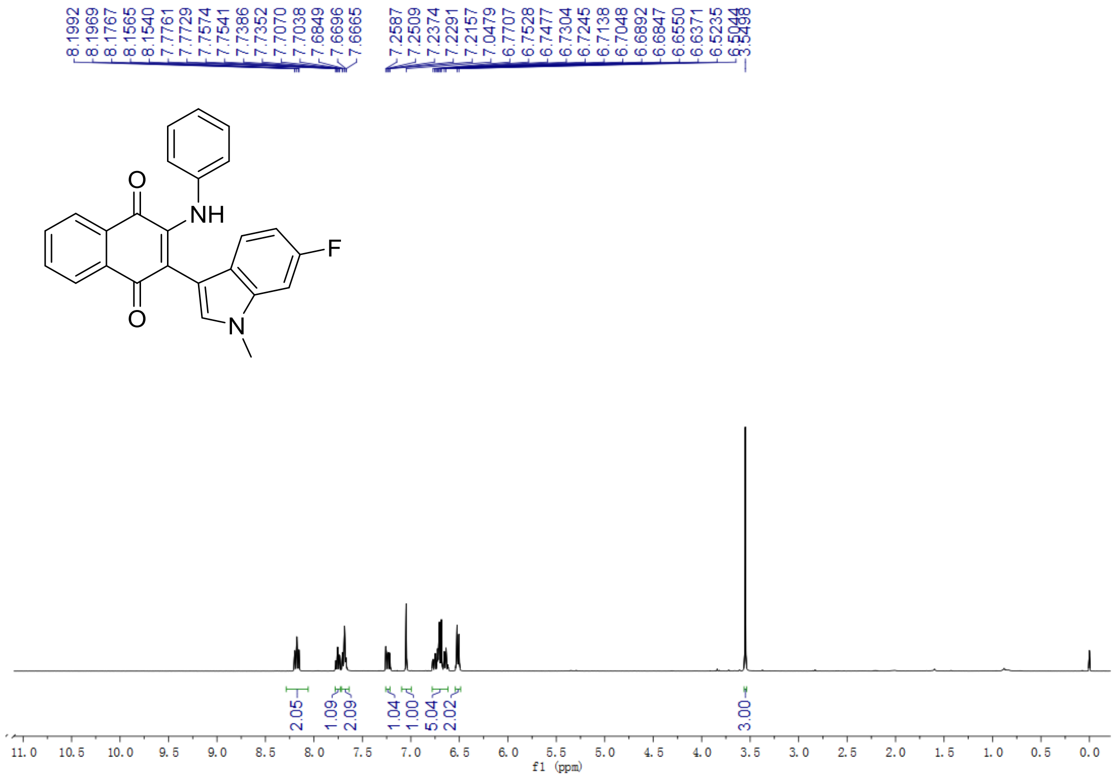

2-(1,7-dimethyl-1H-indol-3-yl)-3-(phenylamino)naphthalene-1,4-dione (3l)

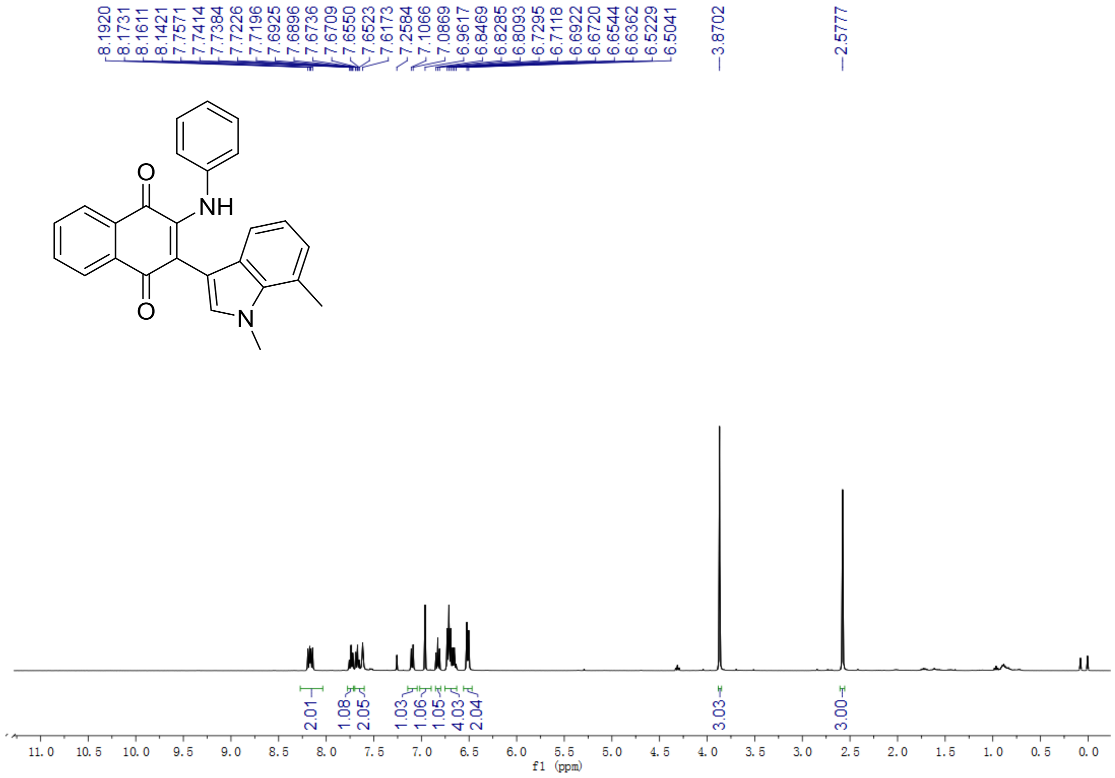

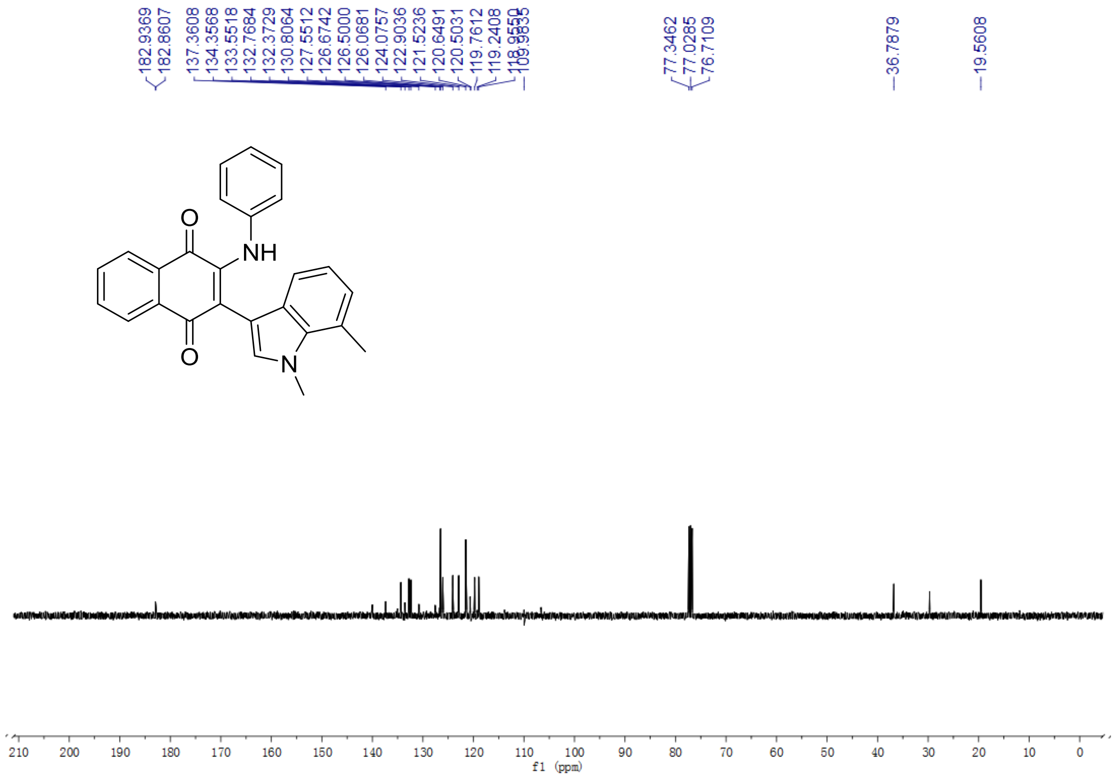

2-phenyl-3-(phenylamino)naphthalene-1,4-dione (3m)

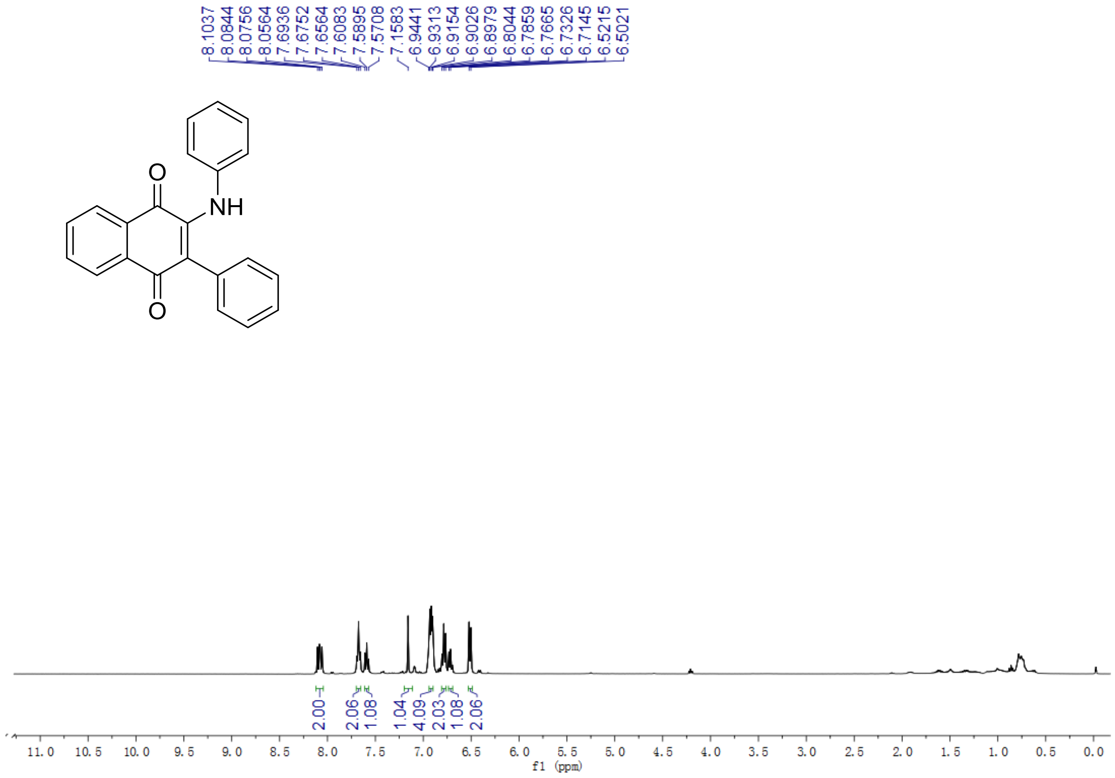

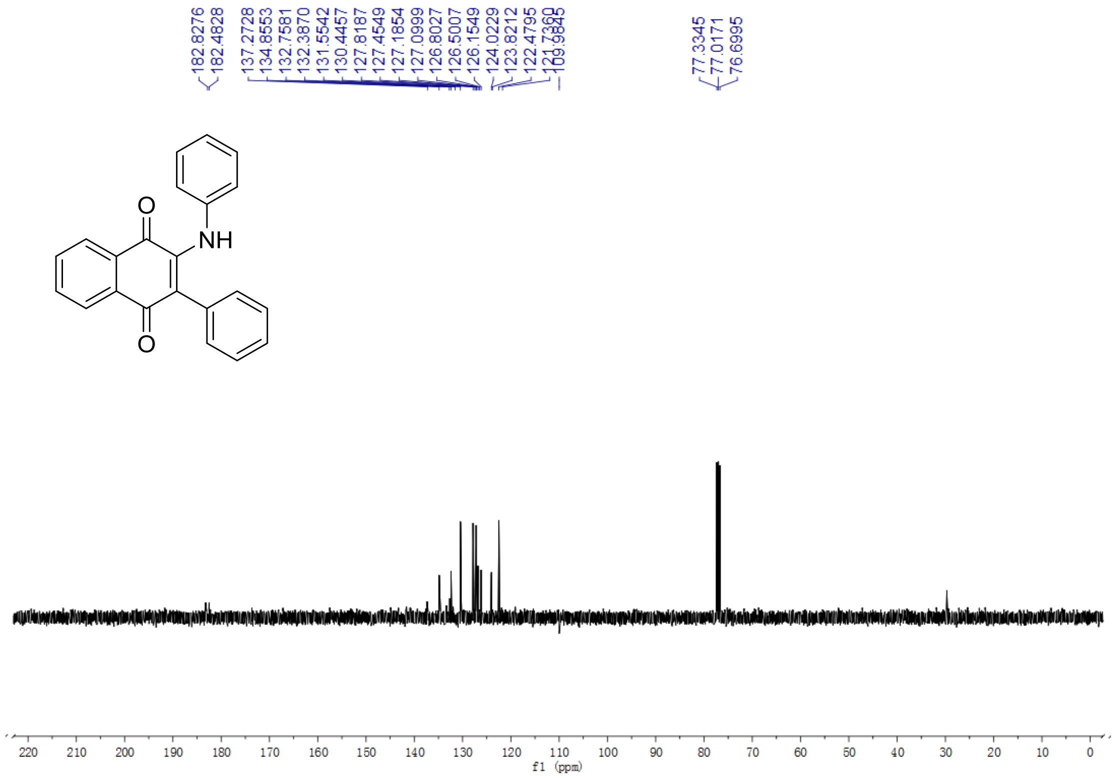

2-(phenylamino)naphthalene-1,4-dione (5a)

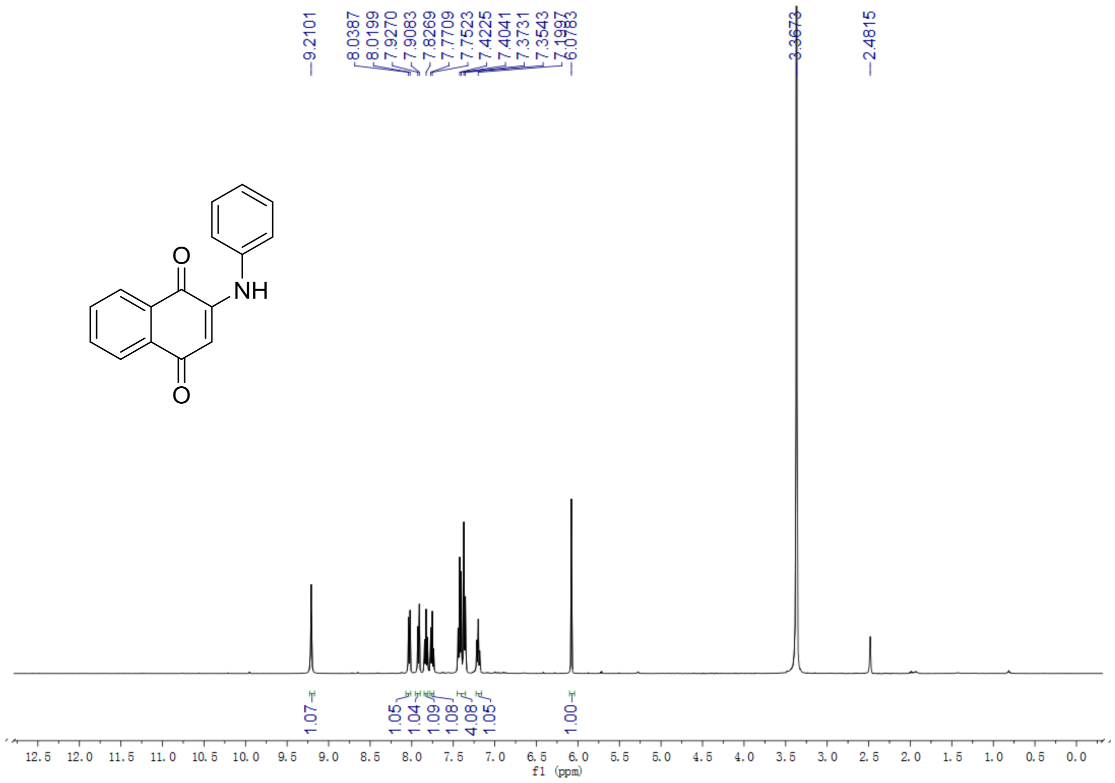

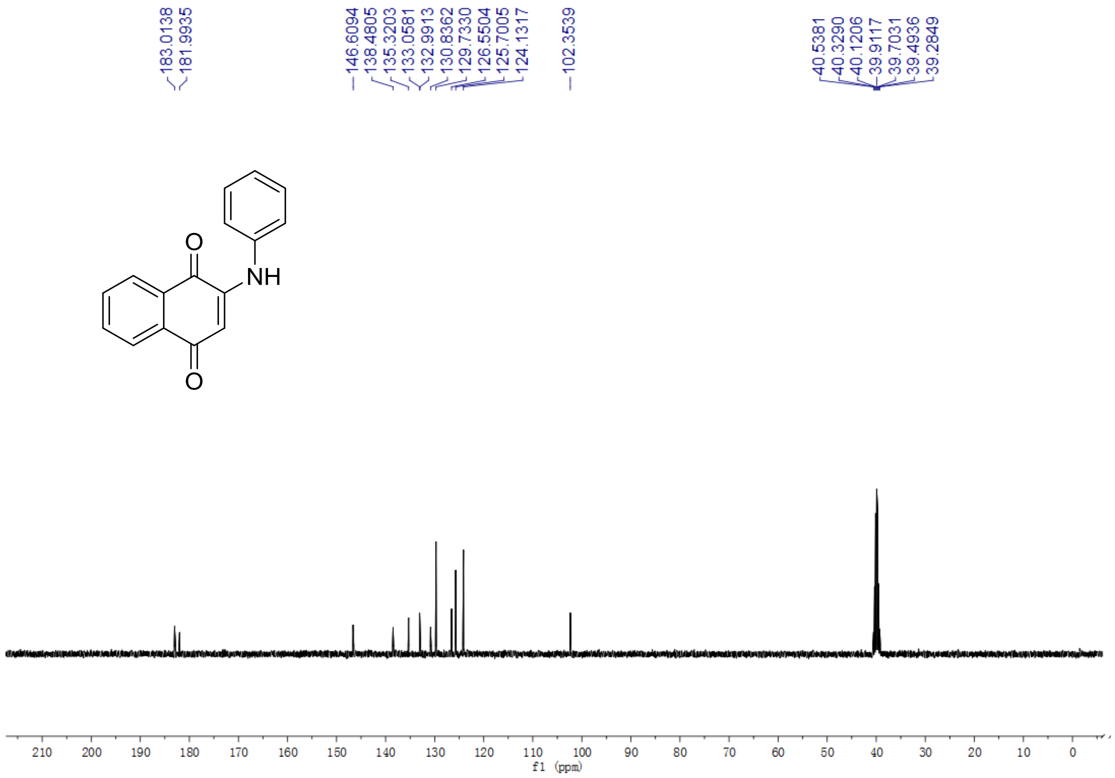

2-(p-tolylamino)naphthalene-1,4-dione (5b)

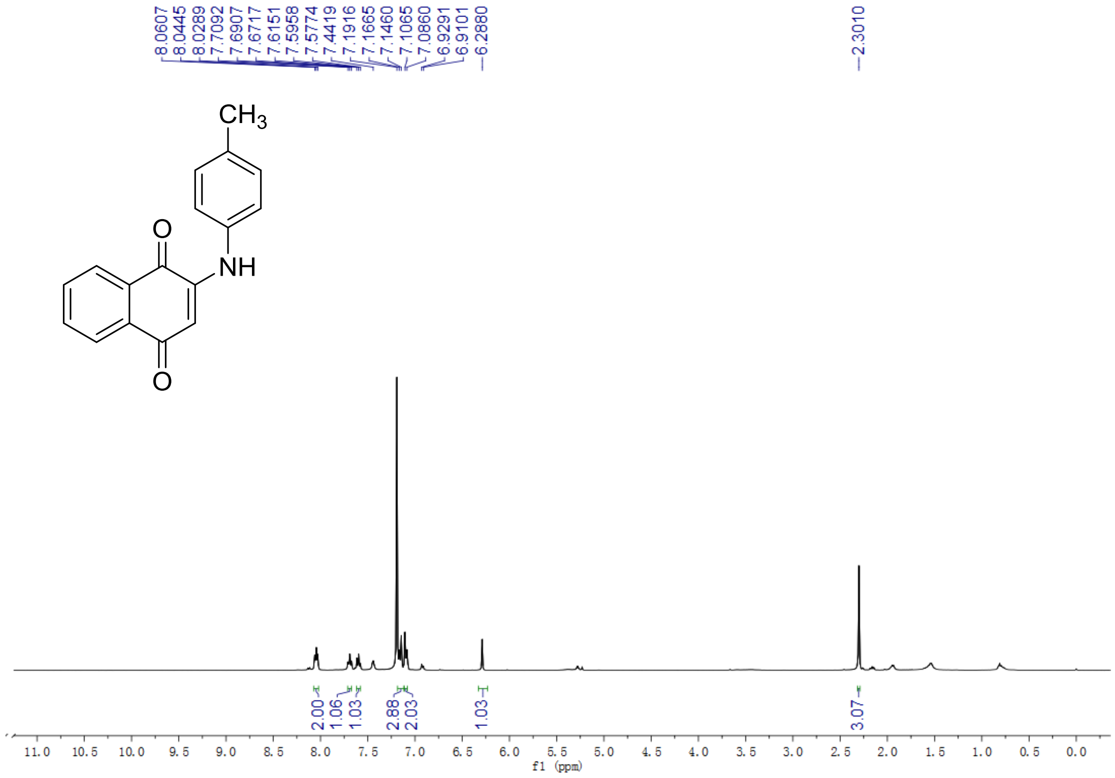

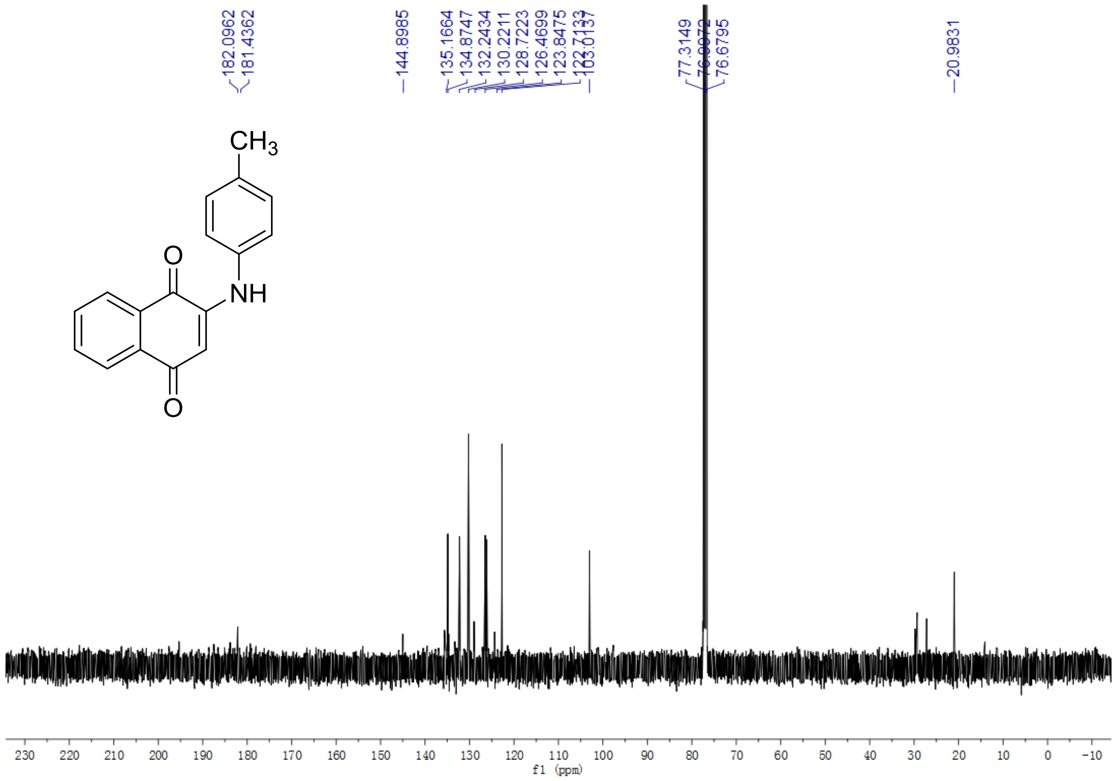

2-((4-(methylthio)phenyl)amino)naphthalene-1,4-dione (5c)

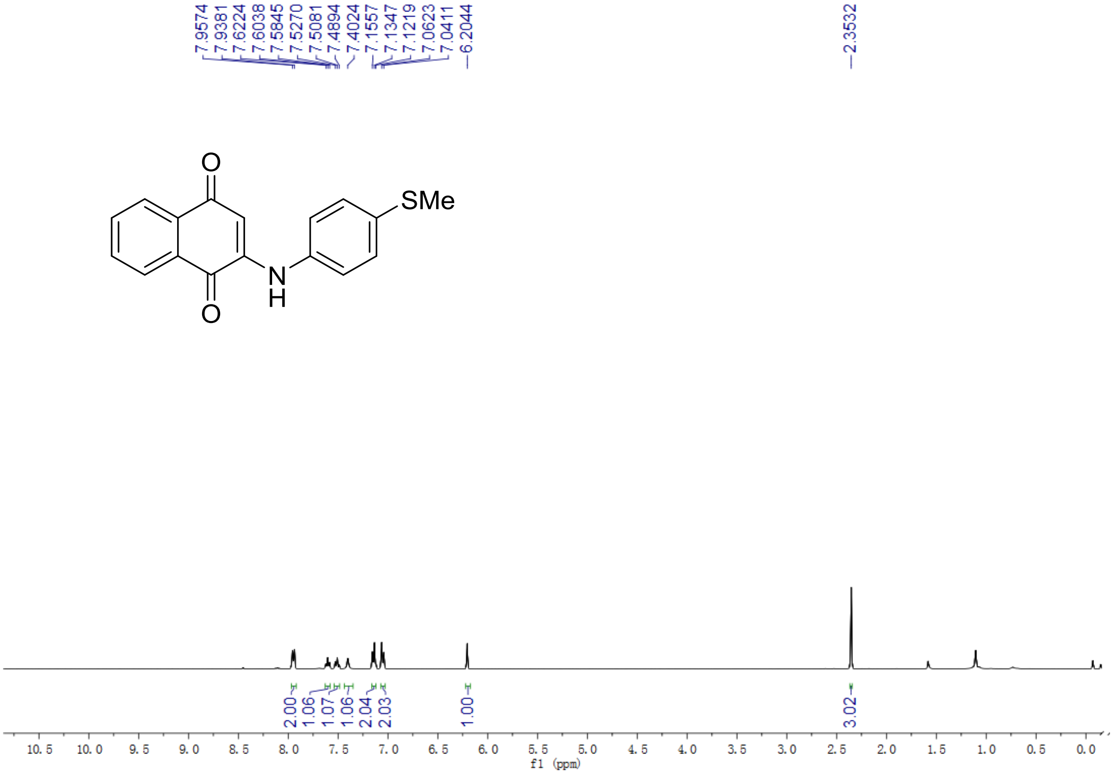

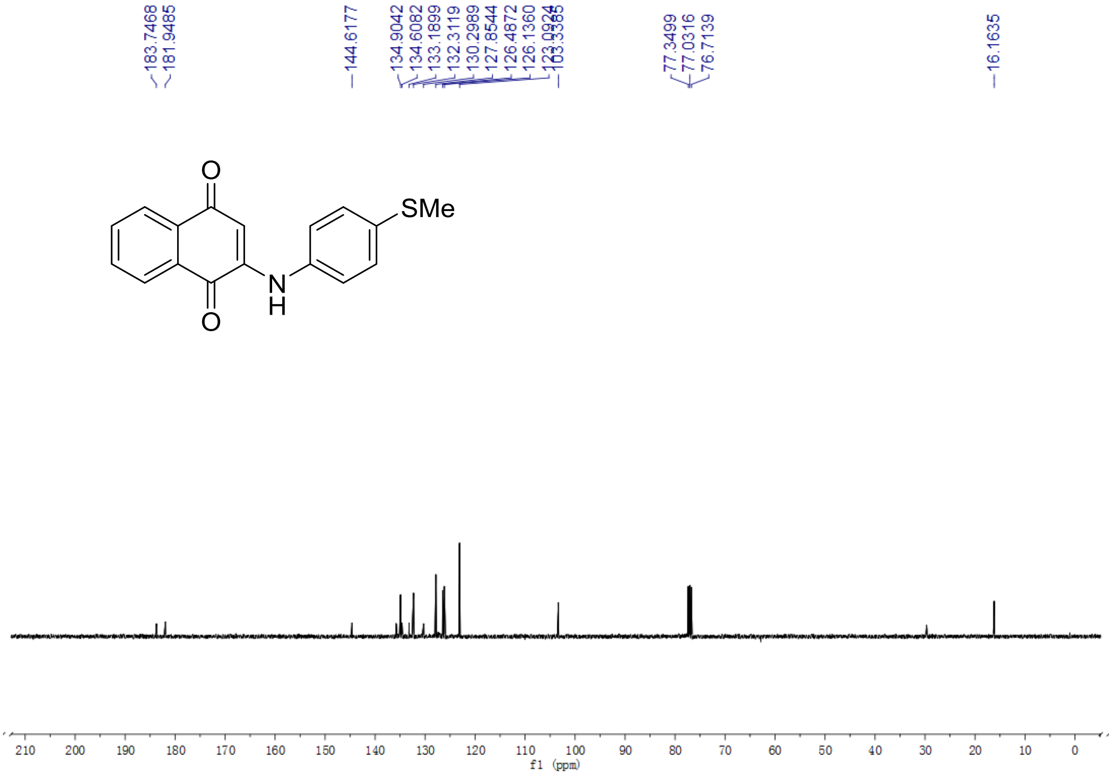

2-((3-methoxyphenyl)amino)naphthalene-1,4-dione (5d)

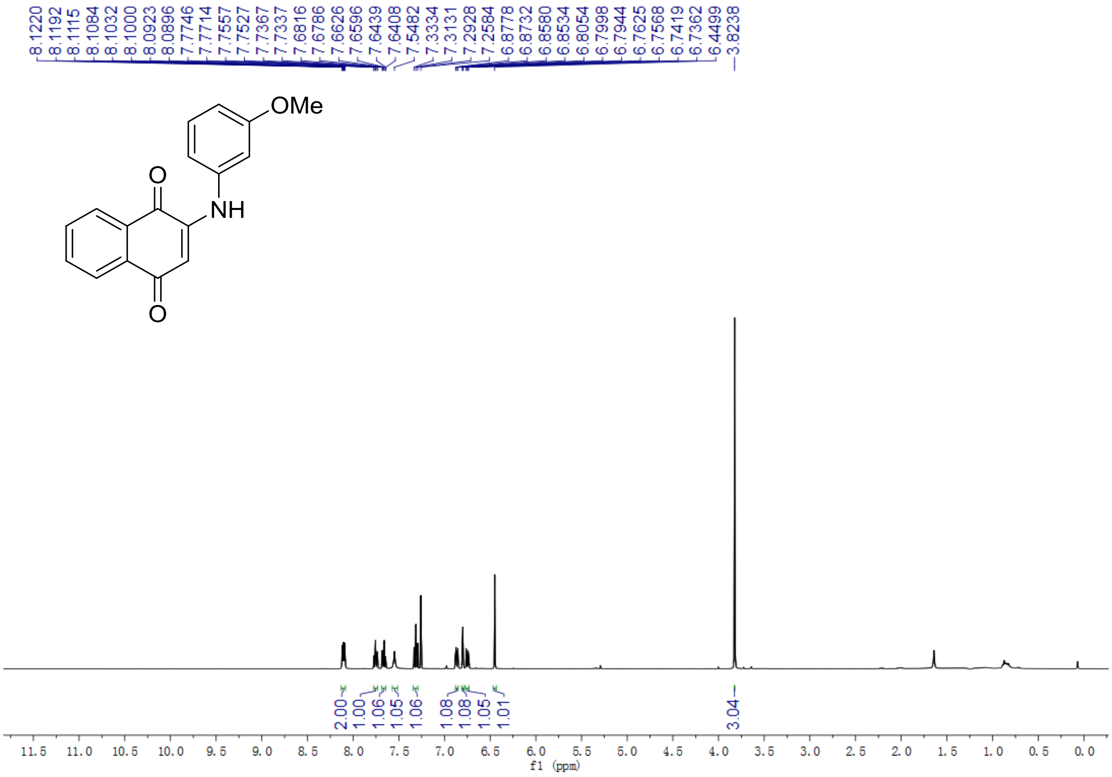

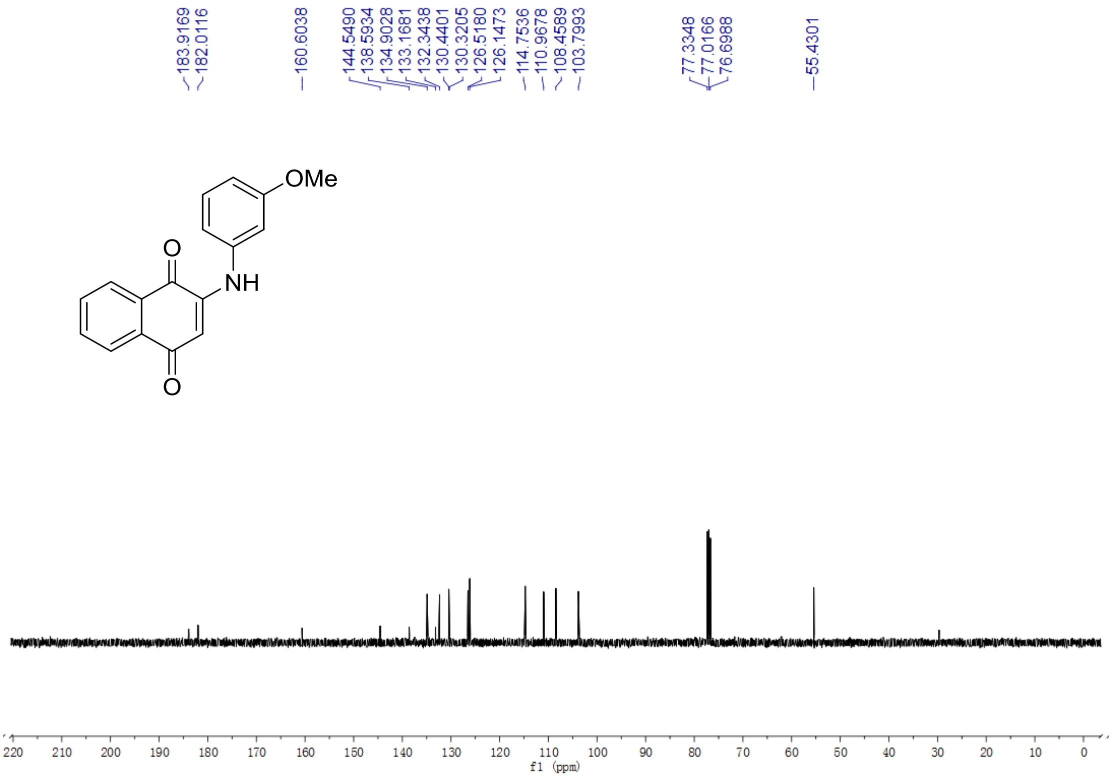

2-((3-chlorophenyl)amino)naphthalene-1,4-dione (5e)

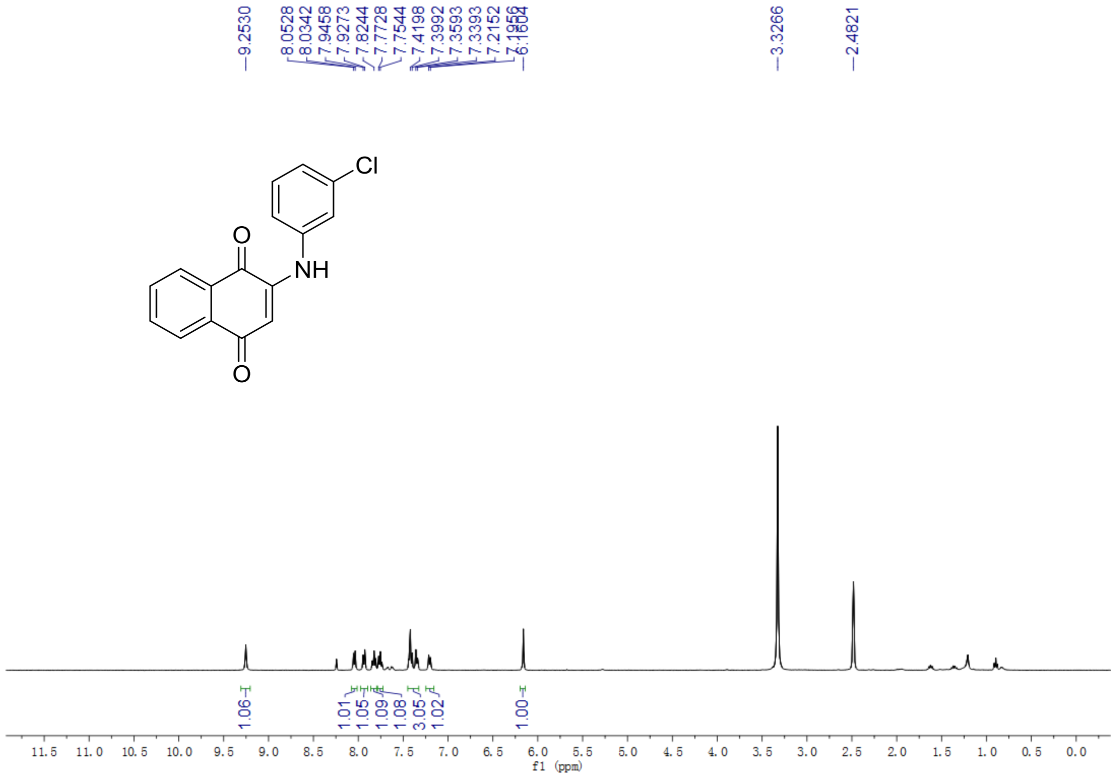

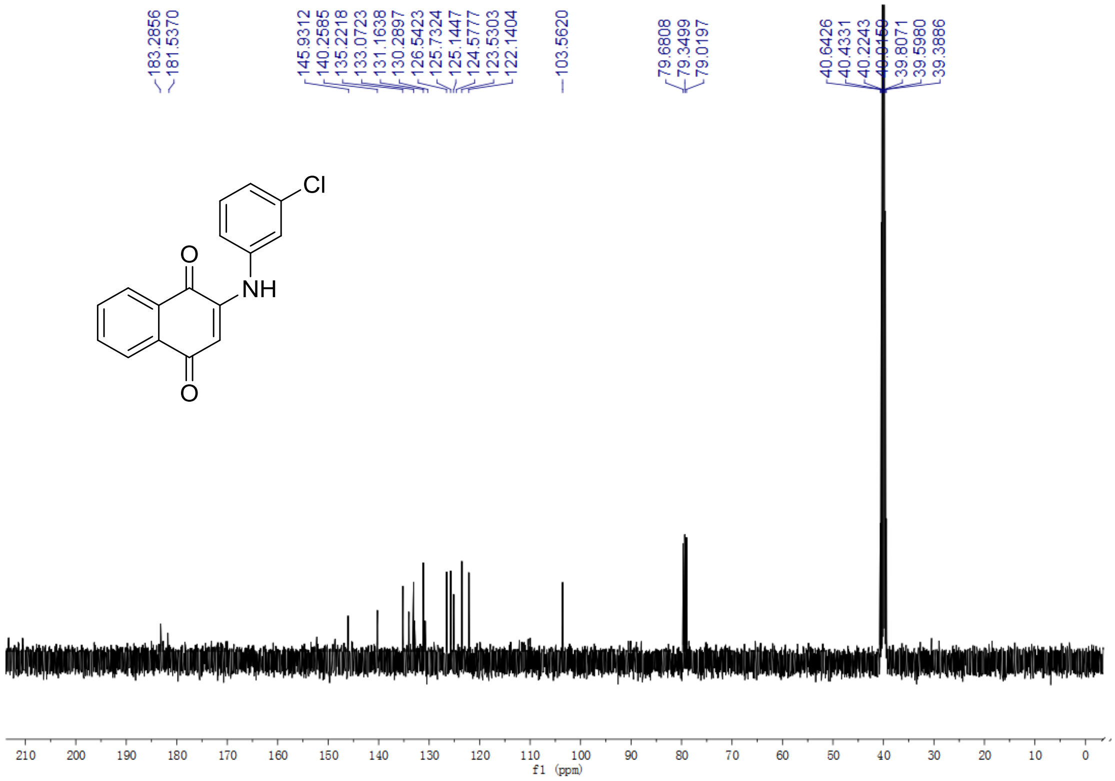

2-(o-tolylamino)naphthalene-1,4-dione (5f)

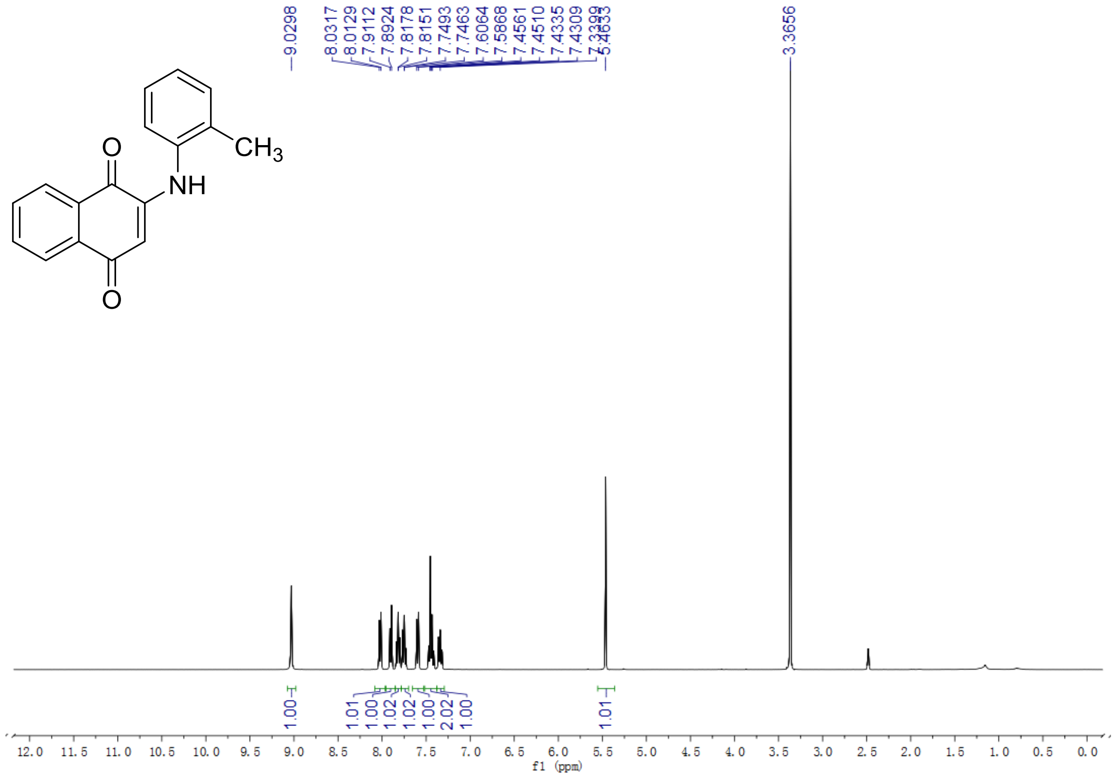

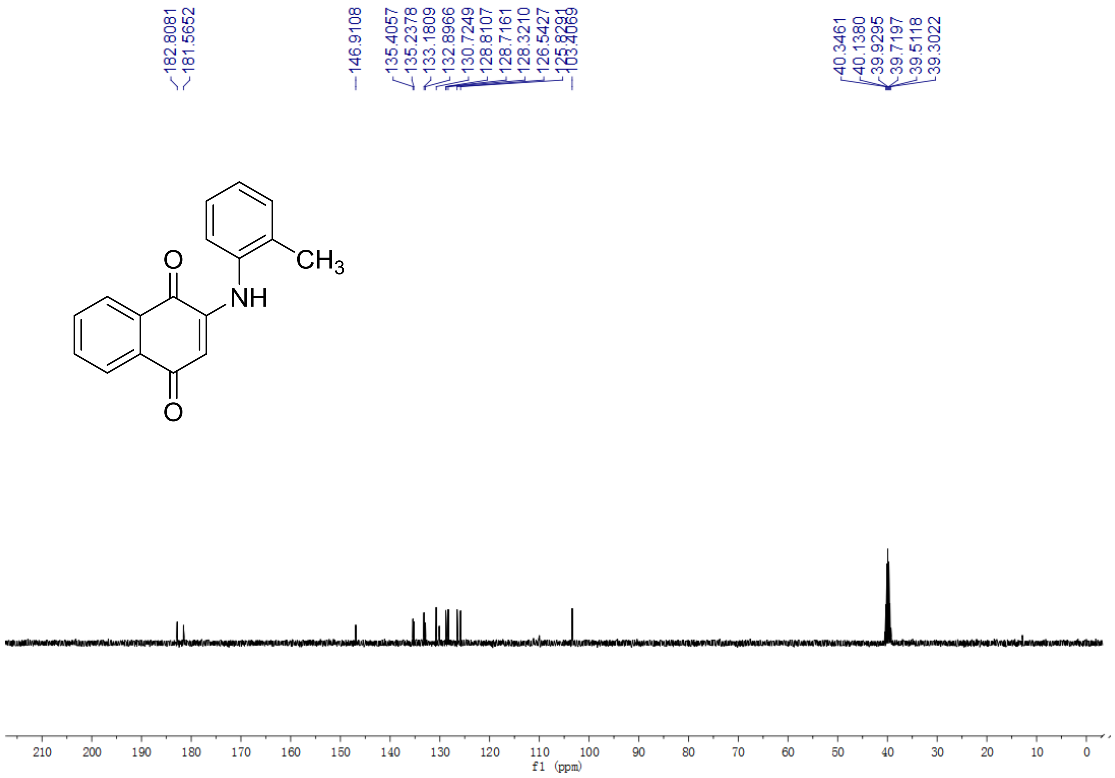

2-((2-hydroxyphenyl)amino)naphthalene-1,4-dione (5g)

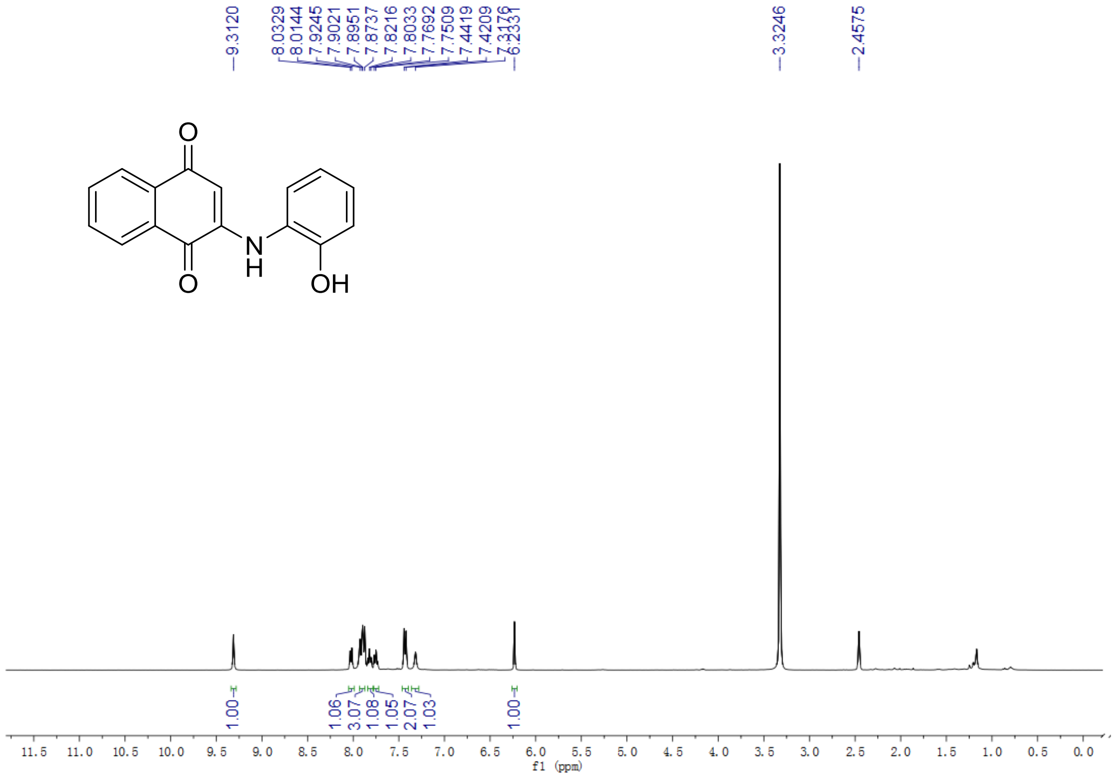

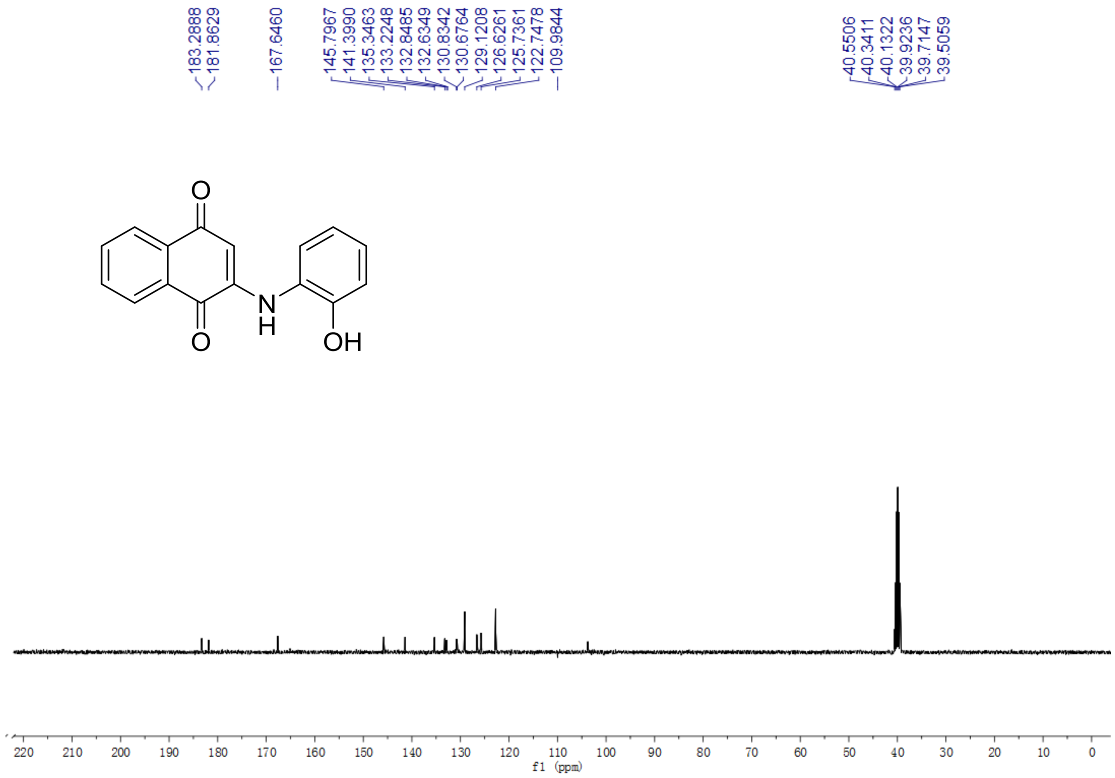

2-((2-chlorophenyl)amino)naphthalene-1,4-dione (5h)

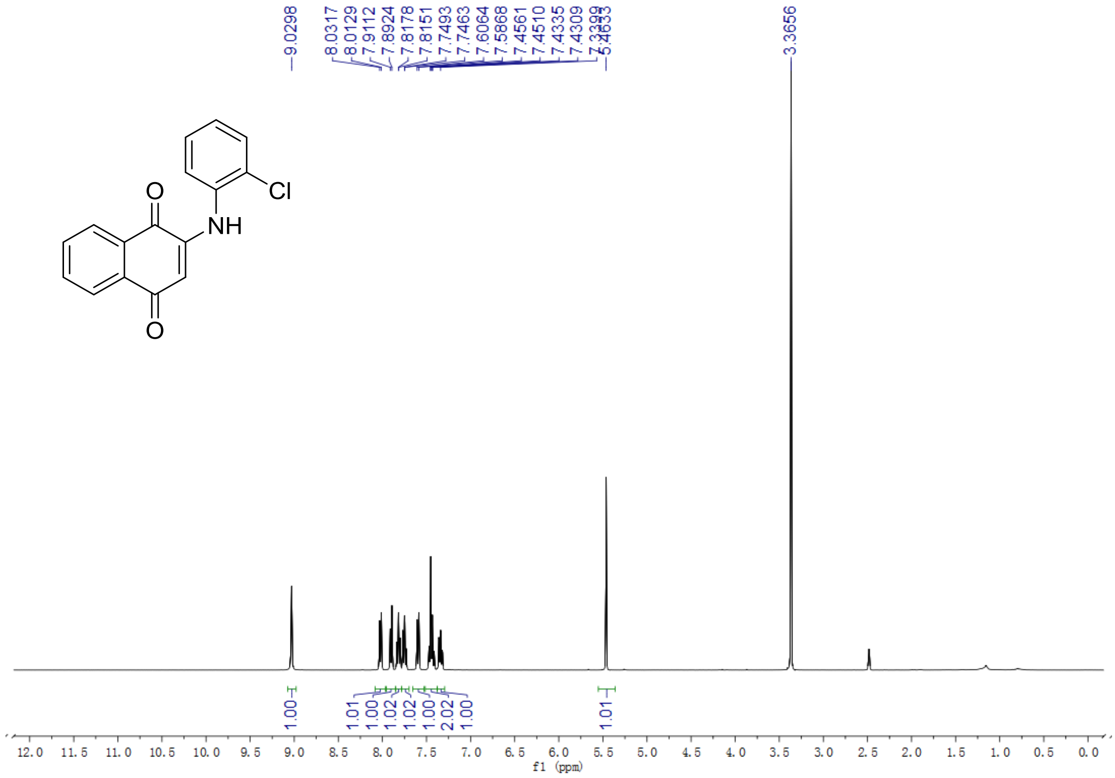

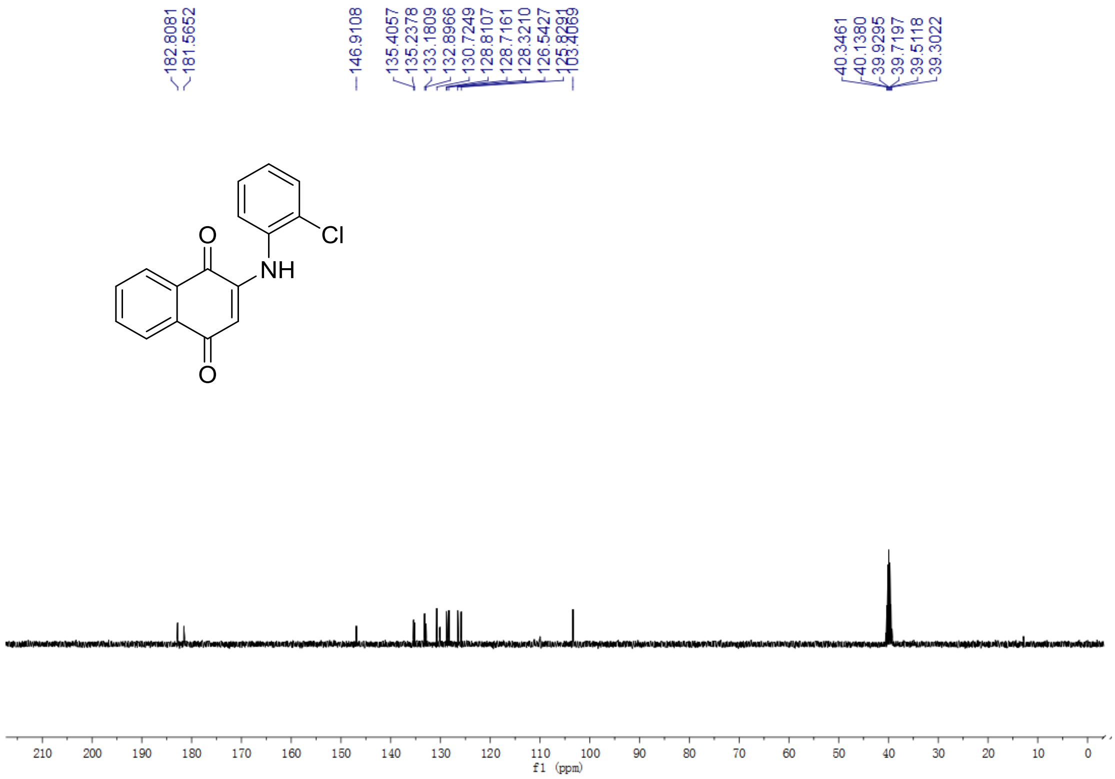

2-(naphthalen-1-ylamino)naphthalene-1,4-dione (5i)

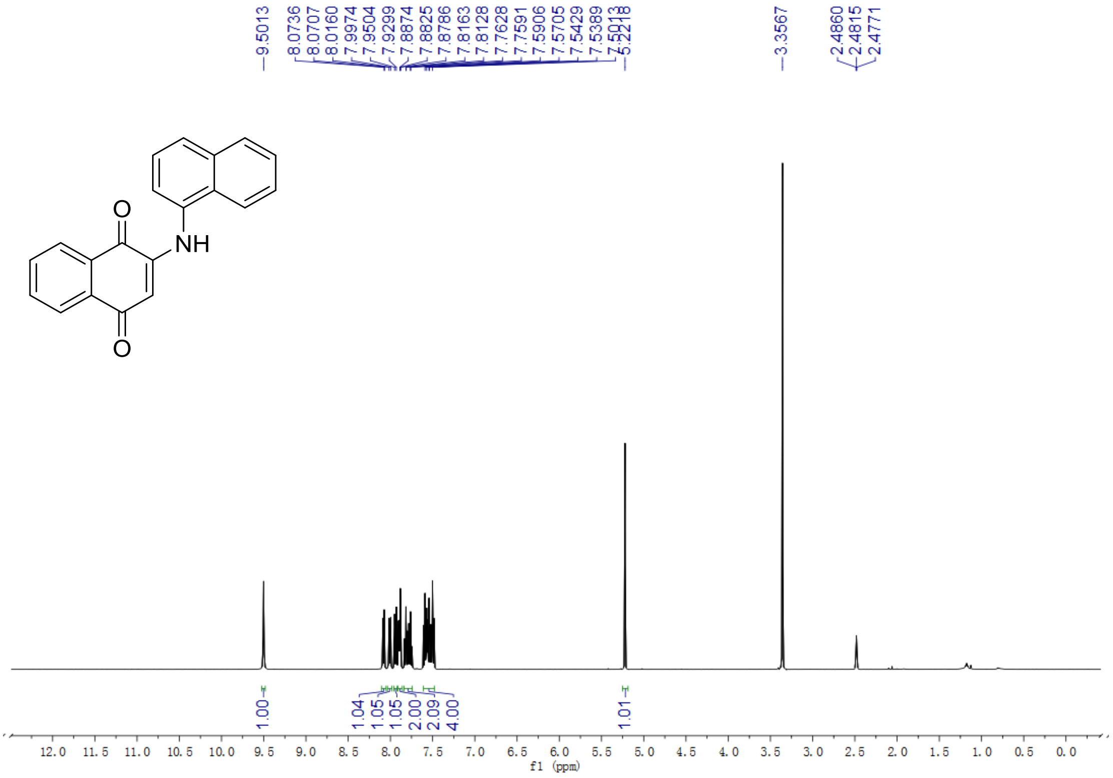

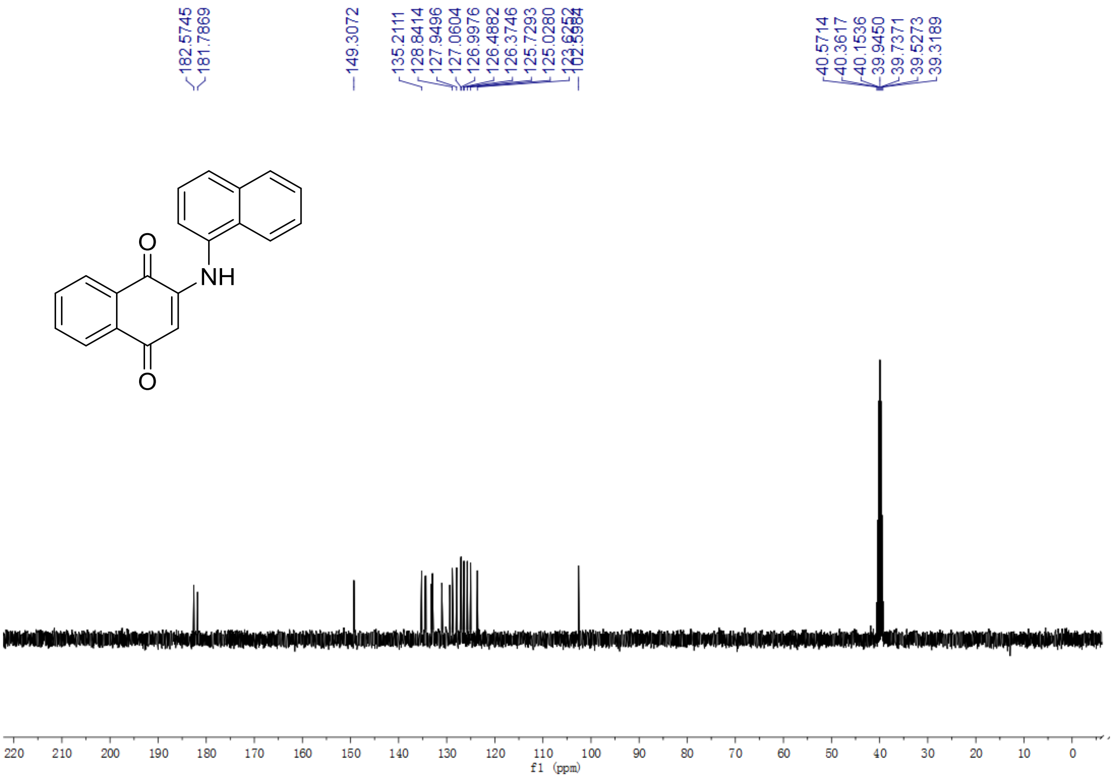

2-((3,5-di-tert-butylphenyl)amino)naphthalene-1,4-dione (5j)

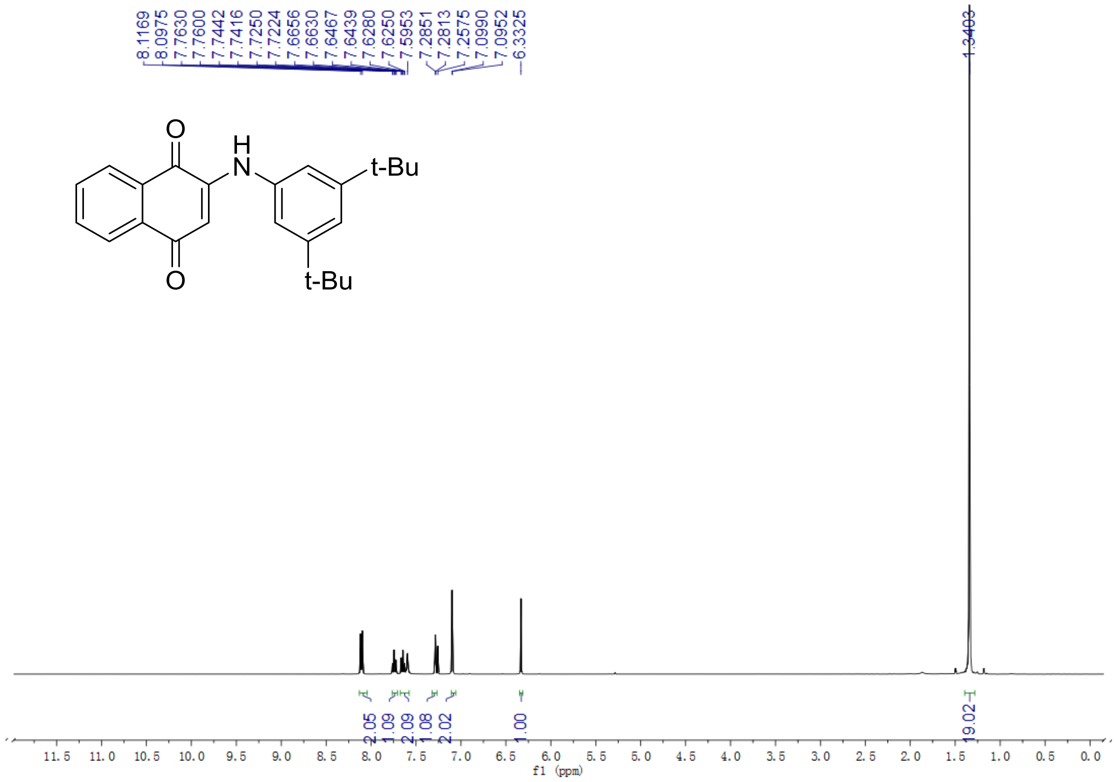

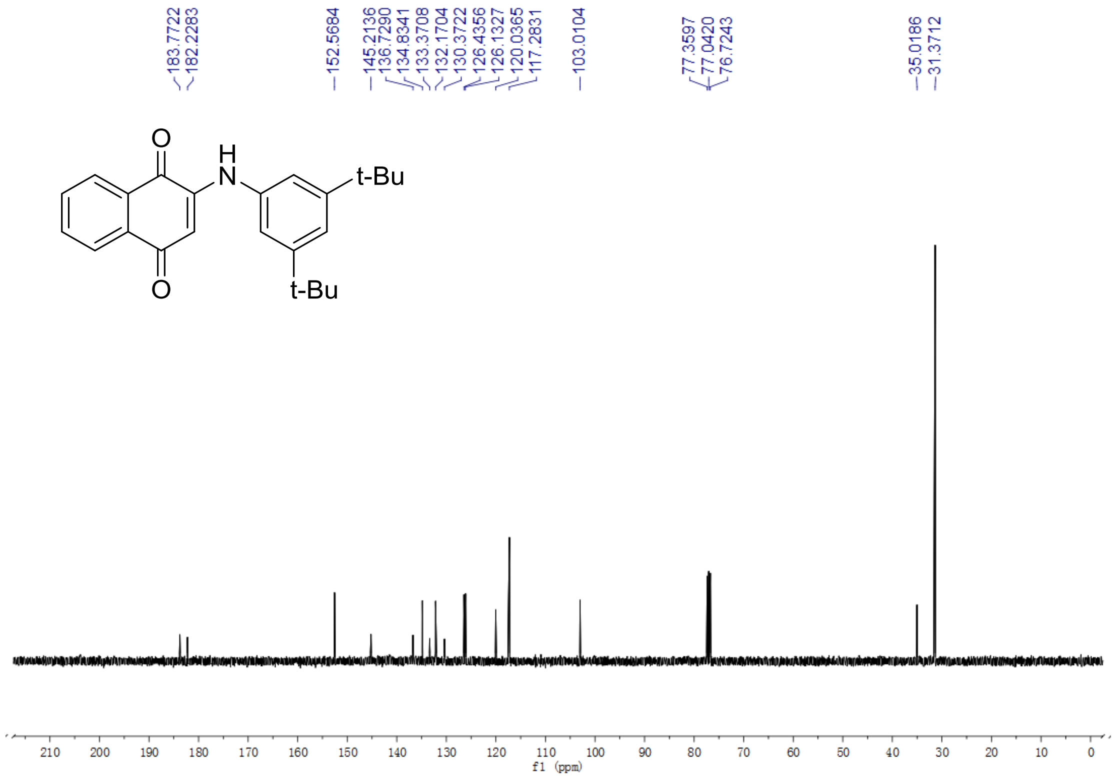

2-(pyridin-2-ylamino)naphthalene-1,4-dione (5k)

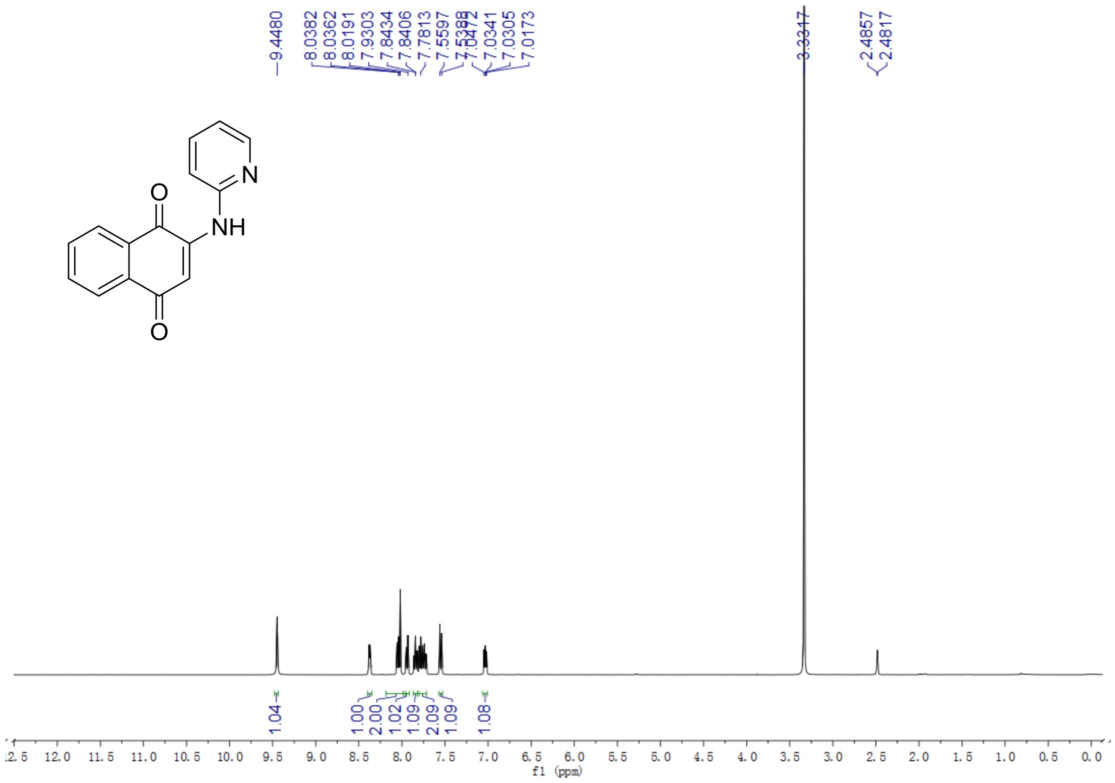

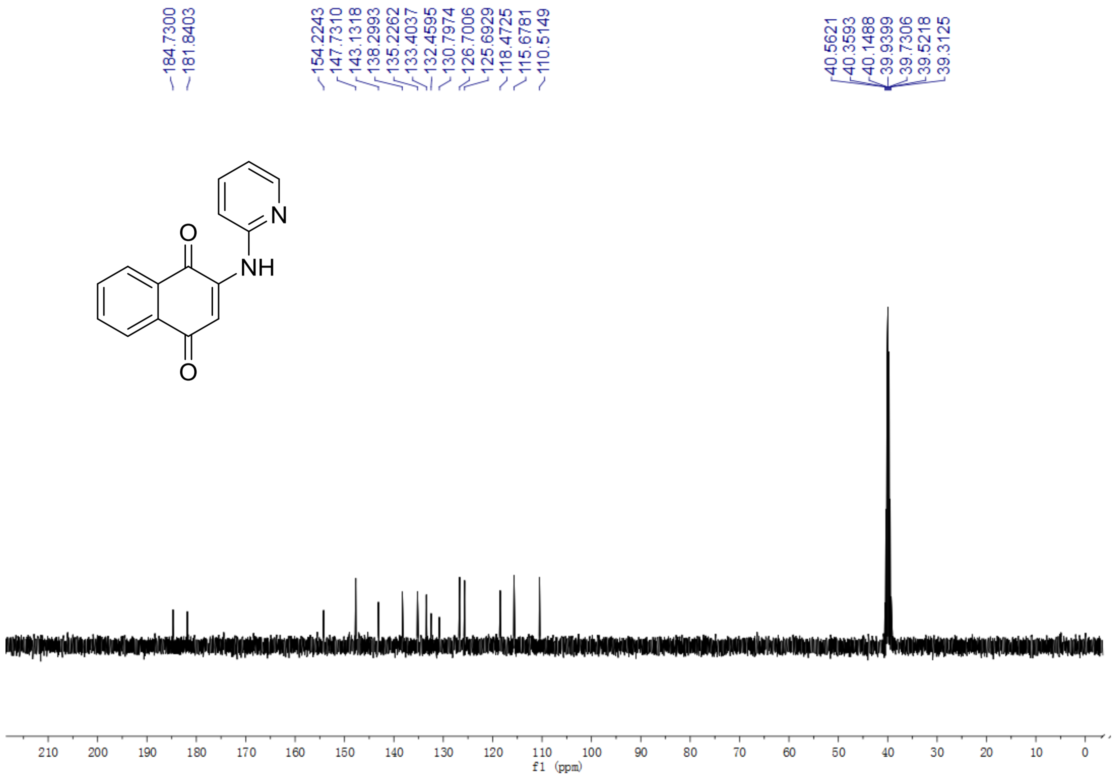

2-(propylamino)naphthalene-1,4-dione (5l)

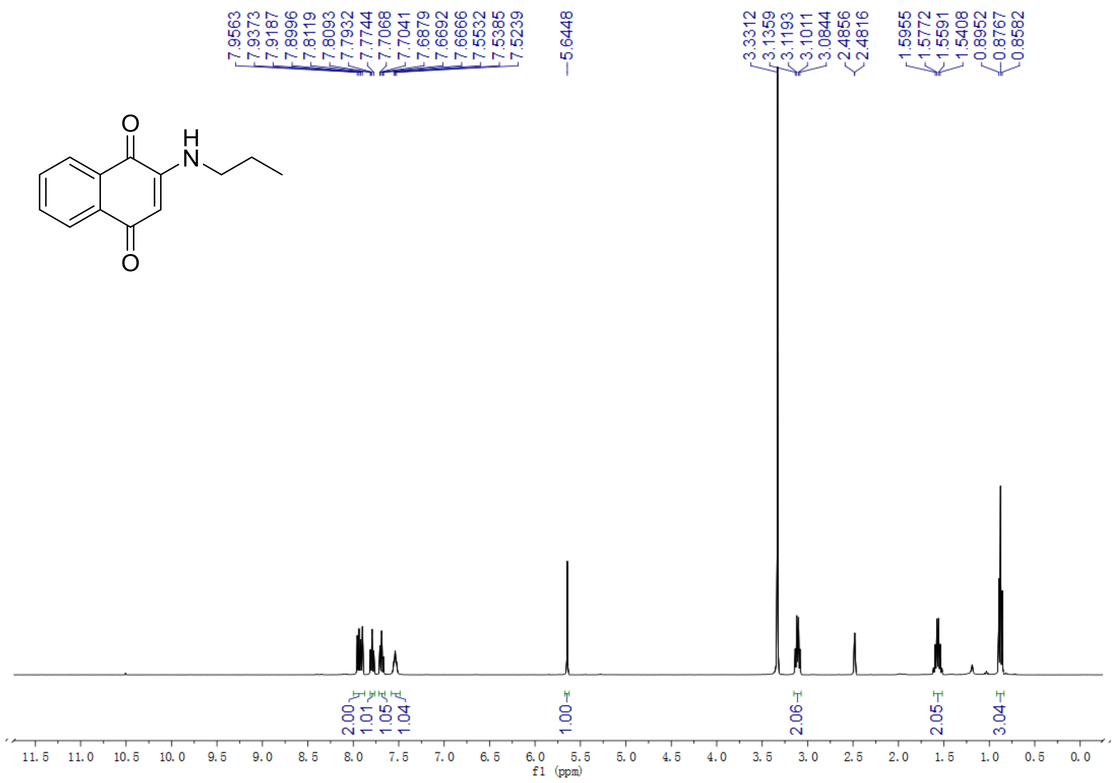

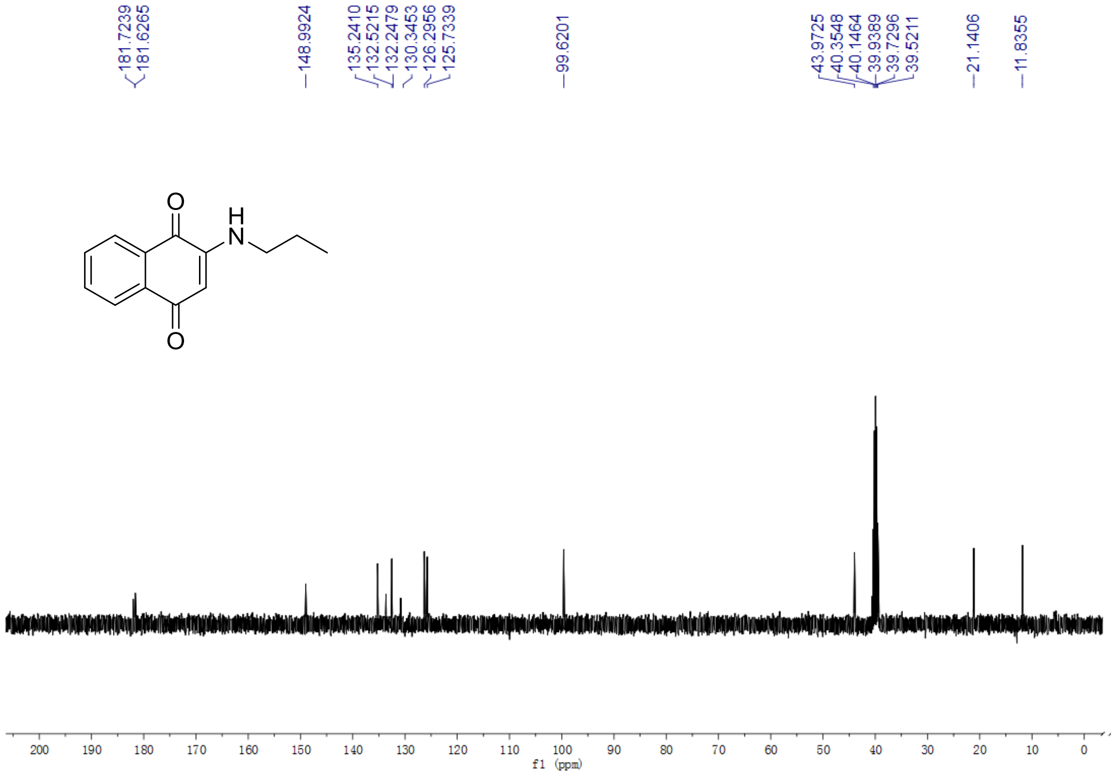

5-methyl-6-phenyl-5,6-dihydrobenzo[f]indolo[2,3-b]indole-7,12-dione (6a)

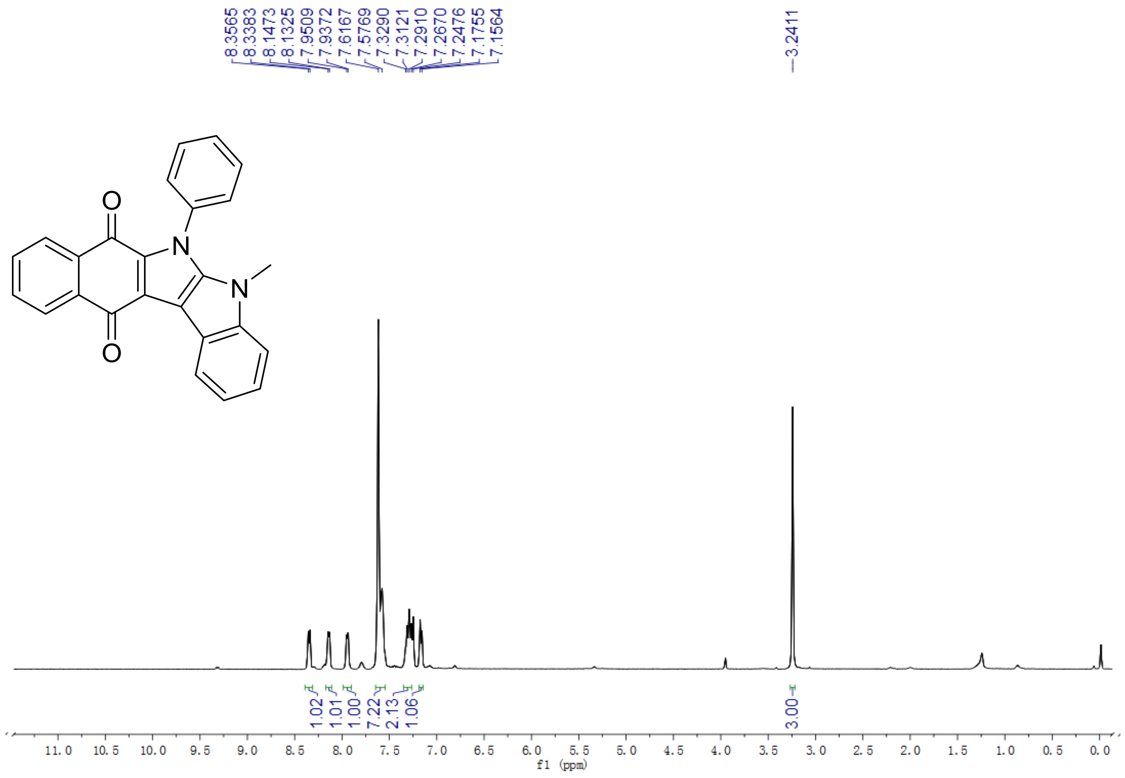

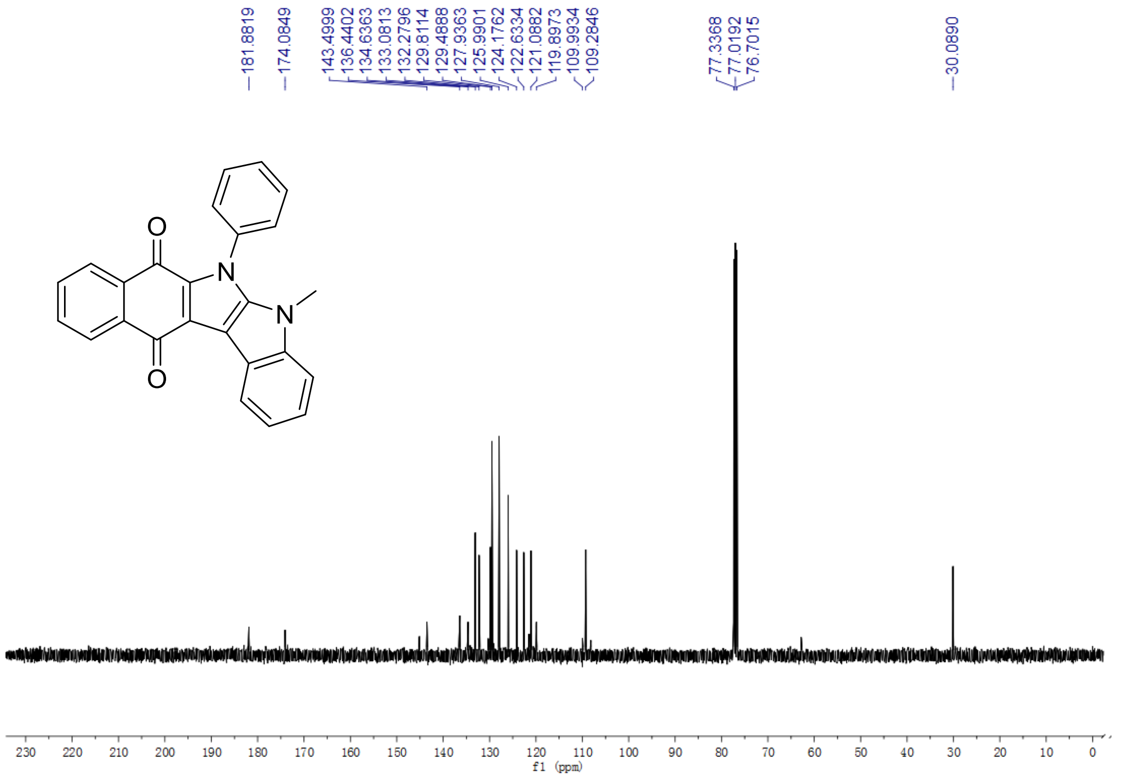

2-chloro-5-methyl-6-phenyl-5,6-dihydrobenzo[f]indolo[2,3-b]indole-7,12-dione (6b)

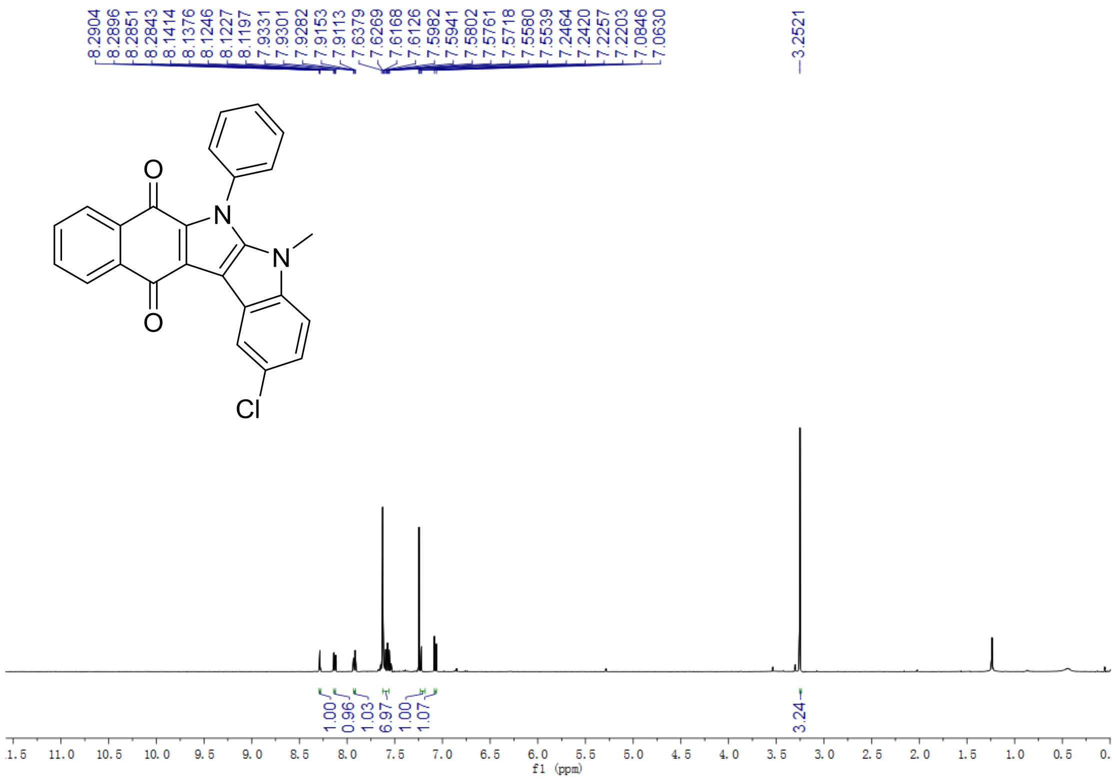

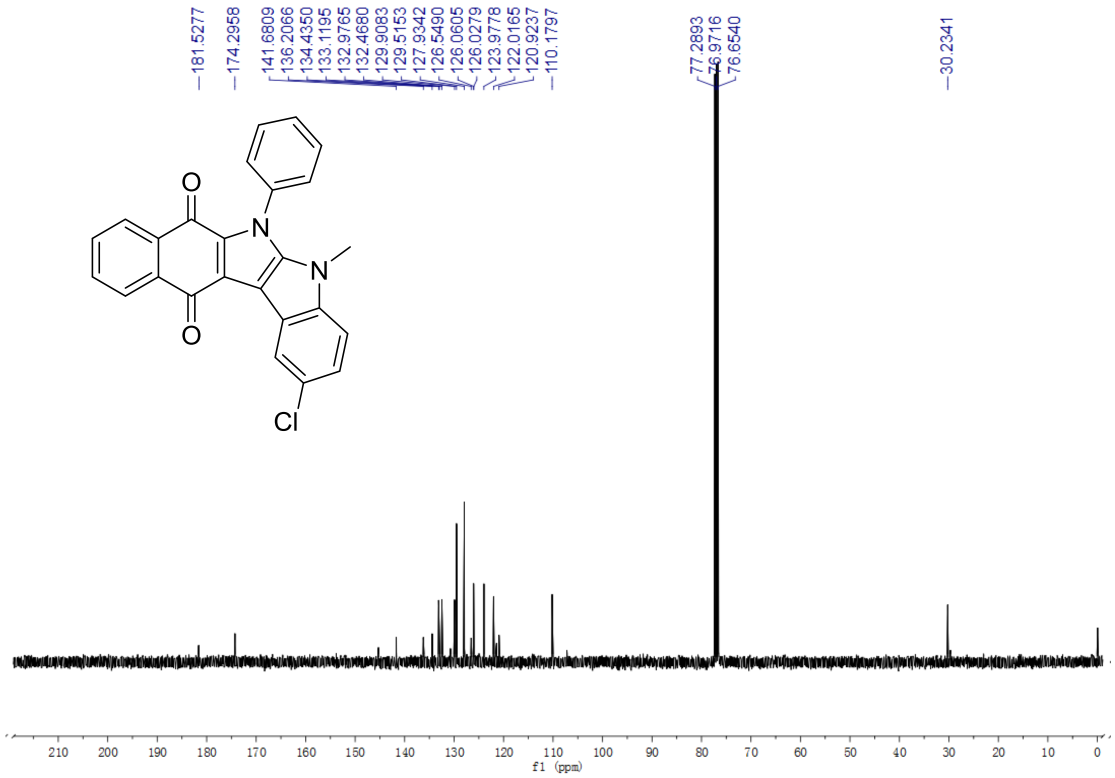

3-fluoro-5-methyl-6-phenyl-5,6-dihydrobenzo[f]indolo[2,3-b]indole-7,12-dione (6c)

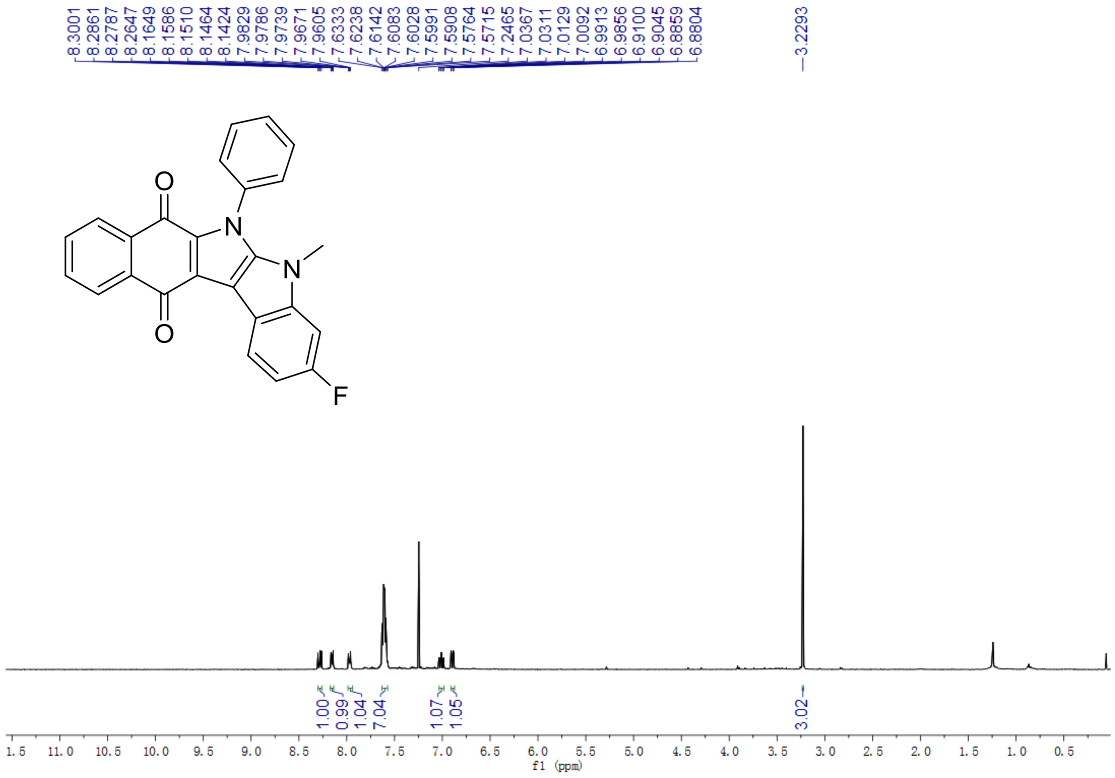

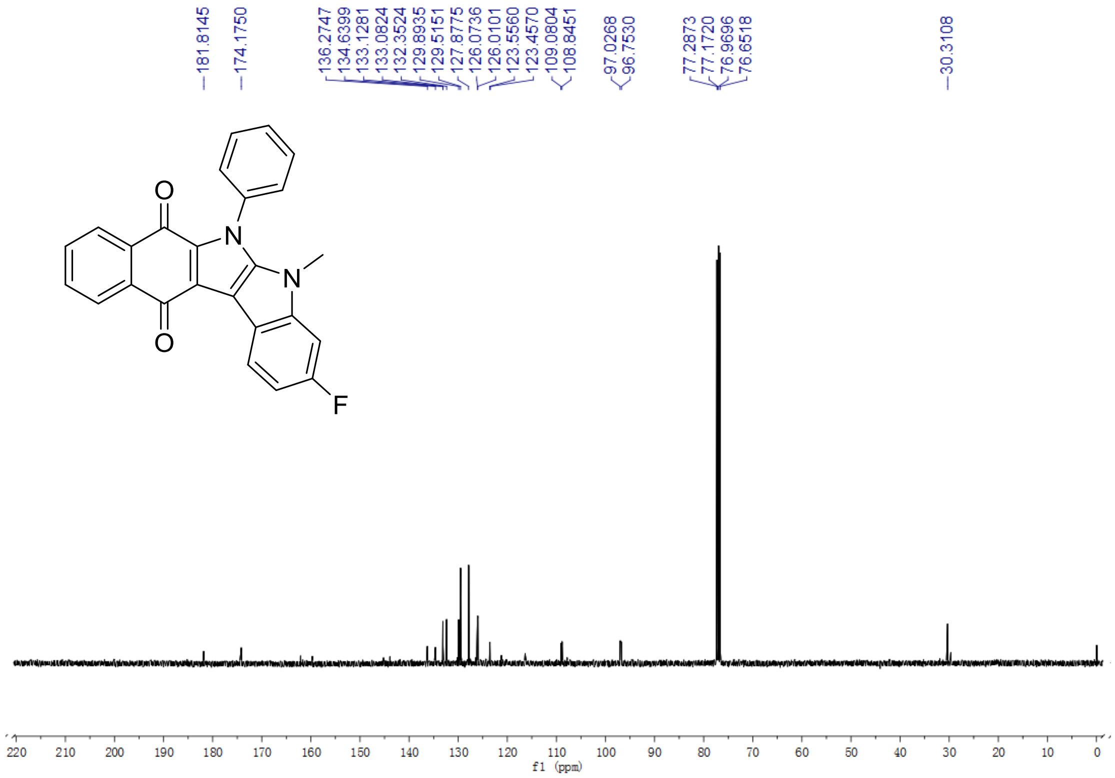

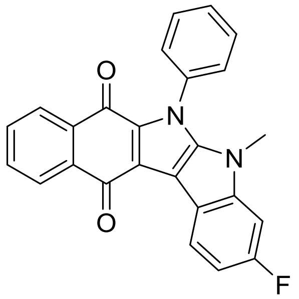
O
N
N
O
F

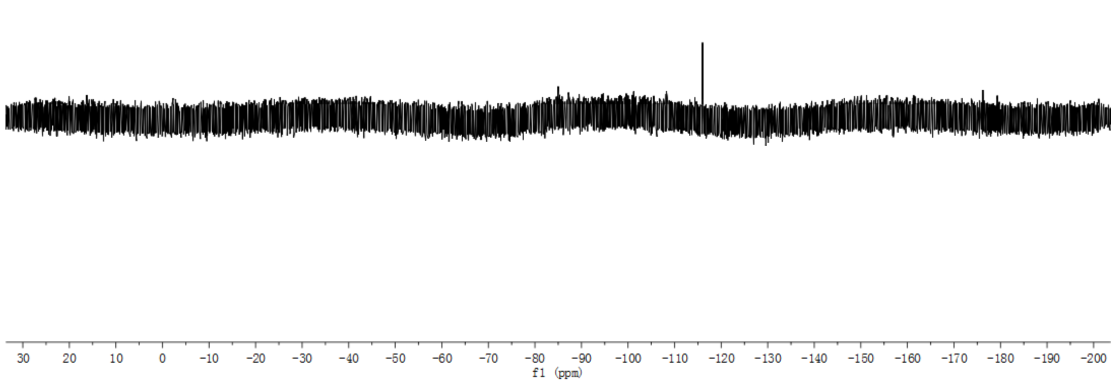
-115.9542
30 20 10 0 -10 -20 -30 -40 -50 -60 -70 -80 -90 -100 -110 -120 -130 -140 -150 -160 -170 -180 -190 -200
f1 (ppm)
